# Characterization of Genes Encoding Poly(A) Polymerases in Plants: Evidence for Duplication and Functional Specialization

**DOI:** 10.1371/journal.pone.0008082

**Published:** 2009-11-26

**Authors:** Lisa R. Meeks, Balasubrahmanyam Addepalli, Arthur G. Hunt

**Affiliations:** Department of Plant and Soil Sciences, University of Kentucky, Lexington, Kentucky, United States of America; Ecole Normale Superieure, France

## Abstract

**Background:**

Poly(A) polymerase is a key enzyme in the machinery that mediates mRNA 3′ end formation in eukaryotes. In plants, poly(A) polymerases are encoded by modest gene families. To better understand this multiplicity of genes, poly(A) polymerase-encoding genes from several other plants, as well as from *Selaginella*, *Physcomitrella*, and *Chlamydomonas*, were studied.

**Methodology/Principal Findings:**

Using bioinformatics tools, poly(A) polymerase-encoding genes were identified in the genomes of eight species in the plant lineage. Whereas *Chlamydomonas reinhardtii* was found to possess a single poly(A) polymerase gene, other species possessed between two and six possible poly(A) polymerase genes. With the exception of four intron-lacking genes, all of the plant poly(A) polymerase genes (but not the *C. reinhardtii* gene) possessed almost identical intron positions within the poly(A) polymerase coding sequences, suggesting that all plant poly(A) polymerase genes derive from a single ancestral gene. The four *Arabidopsis* poly(A) polymerase genes were found to be essential, based on genetic analysis of T-DNA insertion mutants. GFP fusion proteins containing three of the four *Arabidopsis* poly(A) polymerases localized to the nucleus, while one such fusion protein was localized in the cytoplasm. The fact that this latter protein is largely pollen-specific suggests that it has important roles in male gametogenesis.

**Conclusions/Significance:**

Our results indicate that poly(A) polymerase genes have expanded from a single ancestral gene by a series of duplication events during the evolution of higher plants, and that individual members have undergone sorts of functional specialization so as to render them essential for plant growth and development. Perhaps the most interesting of the plant poly(A) polymerases is a novel cytoplasmic poly(A) polymerase that is expressed in pollen in *Arabidopsis*; this is reminiscent of spermatocyte-specific cytoplasmic poly(A) polymerases in mammals.

## Introduction

Eukaryotic messenger RNAs possess characteristic 5′- and 3′- modifications that promote the overall functionality of the molecule. The 3′ modification is an extended poly(A) tract, and serves to promote RNA stability and translatability through interactions with poly(A) binding proteins and translation initiation factors [1]. The poly(A) tract is added posttranscriptionally to mRNAs in the nucleus in a two-step RNA processing reaction; a precursor RNA (or pre-mRNA) is processed at a specific site, and the processed RNA subsequently polyadenylated by a specialized nucleotidyltransferase, poly(A) polymerase. Processing and polyadenylation is mediated by a sizeable complex of factors [2]; this complex recognizes specific sequence elements in the pre-mRNA, cleaves the pre-mRNA at a particular site, and facilitates the addition, by poly(A) polymerase, of the poly(A) tract to the cleaved pre-mRNA. Poly(A) length is controlled by interactions of the poly(A) polymerase and poly(A) itself with a distinctive poly(A) binding protein.

Poly(A) polymerases are broadly conserved enzymes and members of the larger class of nucleotidyltransferases [3]. The canonical nuclear poly(A) polymerase that participates in mRNA 3′ end formation is present in all eukaryotic organisms. In structural terms, all canonical poly(A) polymerases share a conserved N-terminal 450–500 amino acids that includes crucial RNA-binding domains, Mg-coordinating amino acid side chains, the ATP-binding active site of the enzyme, and nuclear localization signals [4], [5]. The C-termini of eukaryotic poly(A) polymerases are more divergent. For example, mammalian poly(A) polymerases have a 200–300 amino acid C-terminus that contains numerous phosphorylation sites [6]. The C-terminus of the yeast enzyme is smaller (ca. 100 amino acids) and consists (in part) of additional RNA-binding domains that are important for overall function of the enzyme (e.g., [7]).

There are a number of other poly(A) polymerases, closely related in sequence, that have functions apart from that of the canonical nuclear poly(A) polymerase. For example, testis-specific poly(A) polymerases have been identified in the mouse [8], [9], [10]. This enzyme is encoded by a gene that is distinct from other poly(A) polymerase genes, since the testis-specific gene lacks introns. The testis-specific poly(A) polymerase is present in the cytoplasm [9], [10] and nucleus [10] of mouse testis cells. Interestingly, it possesses only one (N-terminal) of the two nuclear localization signals that are seen in other mammalian poly(A) polymerases, and lacks the C-terminal domain that is involved in various regulatory modifications and interactions.

In the model plant *Arabidopsis thaliana*, poly(A) polymerases related to the canonical nuclear poly(A) polymerase are encoded by a small four-member gene family [11]. Three of these genes encode polypeptides that are similar in sequence and size, while the fourth encodes a more distantly-related polypeptide that consists almost entirely of the conserved N-terminal portion of the enzyme. The predicted products of all four genes possess poly(A) polymerase activity, and all four are expressed in the plant [11], [12], indicating that none of the four are pseudogenes. Transcripts from all four genes are alternatively-spliced in tissue-specific manners, such that each gene has the potential to encode very small (ca. 200–300 amino acid) polypeptides as well as the full-sized gene products [11].

To better understand the nature of poly(A) polymerase genes in plants, we have undertaken a combined evolutionary, molecular, and genetic analysis of the higher plant poly(A) polymerase gene families. The results reported here reveal that higher plants possess a set of conserved poly(A) polymerase genes that likely arose from a single ancestral gene via a series of gene duplications. They also indicate that all but one of the six rice poly(A) polymerase gene is expressed, albeit to different extents. Furthermore, the results of this study show that each of the four *Arabidopsis* poly(A) polymerase genes is essential, that the promoters of these four genes have distinctive expression properties, and that one of the four poly(A) polymerase proteins is cytoplasmic. Together, these studies reveal a remarkable evolutionary history of duplication and suggest a degree of functional specialization of poly(A) polymerases in plants.

## Results

### Duplication and Diversification of Poly(A) Polymerase Genes in the Plant Lineage

Previous reports have described some properties of the *Arabidopsis* poly(A) polymerase gene family. To determine how wide-spread in plants are the interesting characteristics of this gene family, poly(A) polymerase genes in a number of other plant genomes were identified. For this, the database at Phytozome (http://www.phytozome.net/) was searched using the TBLASTN algorithm [13] and the so-called PAPS4 or PAPS3 proteins (corresponding to the *Arabidopsis* At4g32850.1 and At3g06560.1 proteins, respectively) as queries. This exercise yielded the results shown in Table 1, a collection of genes whose amino acid sequences were derived from full-length cDNAs as well as those whose sequences were deduced by conceptual translation of genomic DNA. From these data, it is apparent that *Chlamydomonas* possesses a single poly(A) polymerase gene, *Physcomitrella patens* and *Selaginella moellendorffii* each possess two possible poly(A) polymerase genes, and the various angiosperms possess between four and six putative poly(A) polymerase genes.

**Table 1:** Putative poly(A) polymerase genes in the plant lineage.

Organism^1^	gene designation or database reference^2^	support for expression (if any)^3^
*Chlamydomonas reinhardtii*	Cre: 126151	
*Physcomitrella patens*	Phypa1_1|30787	
	Phypa1_1|111923	
*Selaginella moellendorffii*	Selmo1|148399	EST
	Selmo1|440295 171521	EST
*Oryza sativa*	Os06g21470 (2 transcripts)	EST, RT/PCR, fl cDNA
	Os06g36360 (4 transcripts)	EST, RT/PCR, fl cDNA
	Os02g13400 (2 transcripts)	EST, RT/PCR, fl cDNA
	Os04g49870	
	Os03g19920	EST, RT/PCR, fl cDNA
	Os07g48890	RT/PCR
*Sorghum bicolor*	Sb01g012650 (complete)	EST
	Sb10g022090 (complete)	EST
	Sb04g008100	
	Sb06g026810	
	Sb01g037200 (complete)	EST
	Sb02g043400 (complete)	EST
*Vitis vinifera*	GSVIVT0001665400 (complete)	fl cDNA
	GSVIVT00033174001 (complete)	EST
	GSVIVT00034292001 (complete)	EST
	GSVIVT00030424001	
	GSVIVT00017746001 (complete)	EST
*Populus trichocarpa*	429736 (LGVIII)	EST
	251405 (LGXV)	
	260254 (LGXVIII)	
	561724 (LGVI)	
*Arabidopsis thaliana*	At1g17980 (2 transcripts)	fl cDNA EST, RT/PCR, array
	At2g25850 (3 transcripts)	fl cDNA EST, RT/PCR, array
	At3g06560	fl cDNA EST, RT/PCR, array
	At4g32850 (10 transcripts)	fl cDNA EST, RT/PCR, array

1 Organism the genome of which was searched using the Phytozome database.

2 Gene designations were obtained from the respective organism database web sites. Where appropriate, parenthetical notes of the reporting of complete sequences (including confirmed C-termini; see the text) and multiple alternatively-processed mRNA isoforms are included.

3 Support for expression was in the form of EST sequences (EST), full-length cDNAs (fl cDNA), microarray data (array), and RT/PCR results (this study).

Amino acid sequence alignments revealed that the greatest conservation in the various predicted proteins listed in Table 1 was within a ca. 500 amino acid portion that encompasses the catalytic core and the RNA-binding domain of the mammalian and yeast poly(A) polymerases (Figure S1). These alignments also revealed a significant divergence in the C-termini). This divergence indicates that poly(A) polymerase sequences from other plant species, if derived by conceptual translations of genomic DNA, must be considered as incomplete, and many of these probably possess unidentified C-terminal extensions. For this reason, more detailed sequence analyses focused on just the conserved core of these proteins.

**Figure 1:**
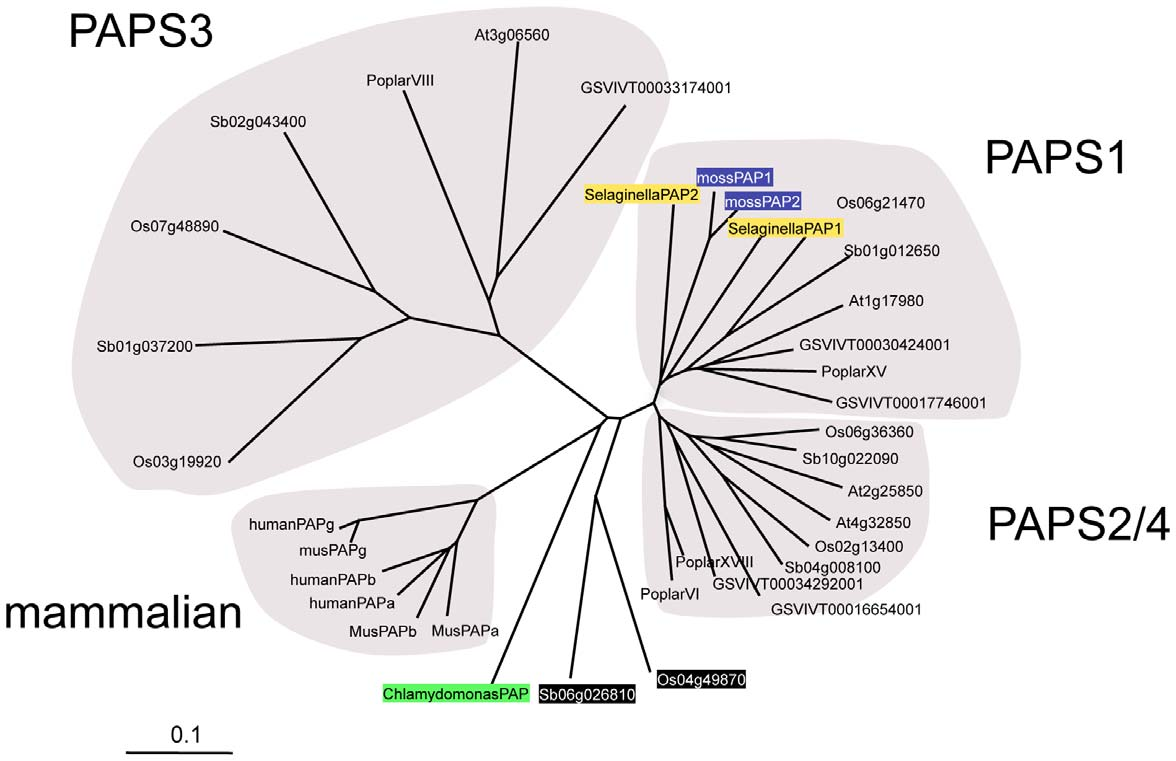
Alignment of the poly(A) polymerase core. The conserved core of the plant poly(A) polymerases, along with the corresponding core of a small set of mammalian poly(A) polymerases, were aligned using EXPRESSO [19]. Sequences used in this alignment are given in File S2. The alignment was displayed as an unrooted tree using Treeview. The four poly(A) polymerase sequence families (the plant families named according to the *Arabidopsis* representatives [12]) are set apart by light gray shading, and individual members of each family noted. For ease of viewing, the *Chlamydomonas* poly(A) polymerase is highlighted with green shading, the two *Selaginella* sequences with yellow shading, the two *Physcomitrella* sequences with deep purple shading, and the two putative grass pseudogenes with black shading and white lettering.

Amino acid sequence comparisons of the conserved cores of the 30 putative poly(A) polymerases revealed that most could be grouped into three classes, typified by the *Arabidopsis* PAPS1, PAPS2/PAPS4, and PAPS3 proteins, respectively (Figure 1; it should be noted that this terminology for the *Arabidopsis* poly(A) polymerases follows that suggested previously [12] and is in accord with conventions for naming *Arabidopsis* genes). Interestingly, *Chlamydomonas*, *Physcomitrella*, and *Selaginella* lacked obvious counterparts for PAPS3. Also, the *Physcomitrella* and *Selaginella* poly(A) polymerases were more similar to PAPS1 than to PAPS2/4. The *Chlamydomonas* poly(A) polymerase was distinct from the other plant poly(A) polymerases, as were the mammalian poly(A) polymerases included in the analysis. Finally, two of the plant poly(A) polymerases (Os04g49870 and Sb06g026810) were distinctly different from all of the other poly(A) polymerases in the study.

To further analyze the plant poly(A) polymerase genes, the intron-exon organizations of these 30 genes were compared with each other, and to poly(A) polymerase genes present in mammals. This analysis (Figure S2) revealed that all but two angiosperm poly(A) polymerase genes share a common intron-exon organization, the exceptions being the rice and sorghum genes (Os04g49870 and Sb06g026810) that are also distinctive in terms of amino acid sequence and their lack of introns. These latter genes lacked intervening sequences. This conserved intron/exon organization was also seen in the two *Selaginella* poly(A) polymerase genes. The two *Physcomitrella* genes, in contrast, possessed no intervening sequences. The *Chlamydomonas* poly(A) polymerase gene possessed intervening sequences, but the intron locations differed from those seen in *Selaginella* and the angiosperm poly(A) polymerase genes. Similarly, the animal poly(A) polymerase genes possessed a conserved intron-exon organization, but one that was different from those seen in poly(A) polymerase genes in the photosynthetic organisms.

### Expression Characteristics of the Rice Poly(A) Polymerase Genes

The presence of what would appear to be duplicated genes for poly(A) polymerases in plants, and particularly the existence of genes lacking introns, raises the possibility that some of the plant genes might be pseudogenes. The four *Arabidopsis* genes have previously been reported to be expressed [11], [12], an observation that argues against this possibility. EST sequences corresponding to many of the other plant genes may be found in databases (as summarized in Table 1); however, the EST collections are likely incomplete, so conclusions about genes for which no EST evidence exists may not be drawn. Thus, to explore this matter further, the expression of the rice poly(A) polymerase gene family was studied by RT/PCR and RNA blotting. Rice was chosen because its poly(A) polymerase genes are representative of the entire range of poly(A) polymerase genes seen in the eight species examined; in particular, it has members of all three poly(A) polymerase classes, and has an intron-lacking gene (Figure S2) encoding a protein that seems distantly related, at best, to other plant poly(A) polymerases (Figure 1). In this experiment, no discernible expression of the intron-lacking poly(A) polymerase gene (Os4g49870) would be seen, even when using the sensitive RT/PCR method (not shown). The expression of one gene (Os07g48890) was exceedingly low, such that partial cDNA clones (that spanned one or more introns, and thus were derived from spliced mRNAs) could be obtained by RT/PCR, but full-length cDNAs could not be generated. The other rice poly(A) polymerase genes were expressed, such that full-length cDNAs could be amplified by RT/PCR, cloned, and sequenced (see File S1).

**Figure 2:**
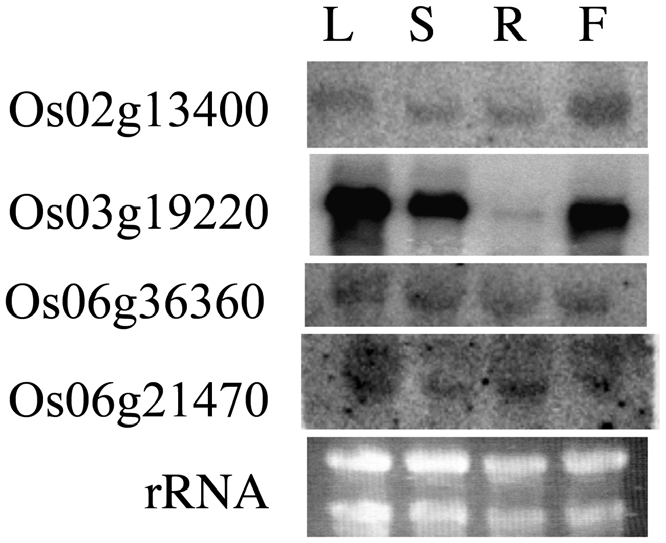
Expression profile of four of the rice poly(A) polymerase genes. 10 µg of total RNA isolated from the indicated tissue (denoted on the top: L – leaf, S – stem, R – root, F - flower) was separated on agarose gels, transferred to nylon membranes, and probed with labeled probes specific for each poly(A) polymerase gene (these were derived from the 3′ ends of each mRNA). In addition, in one case, the separated RNAs were stained with ethidium bromide to indicate the quantity and quality of the RNA preparation. The source of the probe is indicated on the left.

The expression of four of the rice poly(A) polymerase genes could be detected by RNA blotting (Figure 2). These experiments revealed a modest bias in the expression of these genes in different tissues. Os2g13400 was expressed somewhat uniformly in the four tissues sampled (leaves, stems, roots, and flowers), although expression in flowers was somewhat higher. Os6g21470 and Os6g36360 were also expressed throughout the plant, at levels near the limits of detection using the RNA blotting assay. In contrast to the somewhat uniform expression of these three poly(A) polymerase genes throughout the plant, Os3g19220 was expressed in leaves, stems, and flowers, but not in roots. The levels of expression of one of the rice poly(A) polymerase genes (Os07g48890) was beneath the detection limits of the RNA blotting assay. From these results, it may be concluded that at least four of the six rice poly(A) polymerase genes are expressed, one has very low expression and the other, if expressed, is at levels that are beneath the limits of detection of the assays used in this study.

### Each of the Four Arabidopsis Poly(A) Polymerase Genes Is Essential

**Figure 3:**
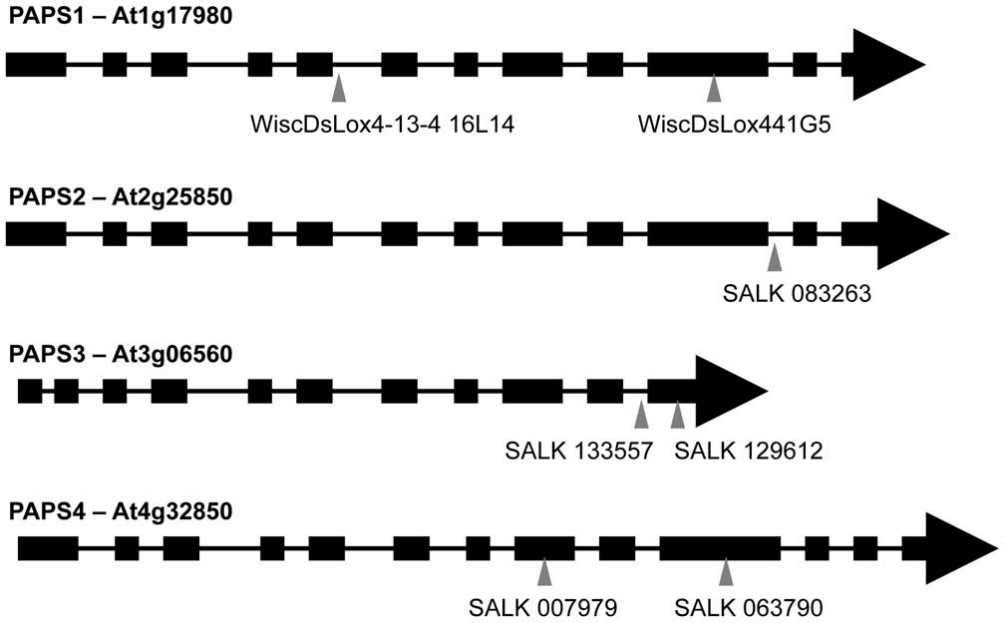
Position of insertions in the four *Arabidopsis* poly(A) polymerase genes. Each of the four *Arabidopsis* poly(A) polymerase genes is represented as a series of exons (large black boxes) interrupted by intervening sequences (thin lines). The exons are drawn roughly to scale, but the introns are not. Beneath each representation is shown the approximate location of the insertion elements for the seven mutants analyzed in this study. The *Arabidopsis* gene designations and insertion mutant identifiers are as in Tables 1 and 2.

Two basic alternatives exist regarding the possible functionality of expressed members of gene families – they may be functionally redundant, providing the same activity in many or most cells, or they may be specialized, either in activity or expression. One means to distinguish between these possibilities is to test whether individual gene family members are essential. To this end, a selection of mutants with T-DNA insertions within each of the fours *Arabidopsis* poly(A) polymerase genes was studied. At least one line for each poly(A) polymerase gene family member was identified in the SIGnAL T-DNA express database [14] or the WiscDsLox T-DNA collection [15]; the relative positions of each insertion in these genes is shown in Figure 3. A PCR genotyping assay was used to analyze at least 35 individual T2 plants from each line. Results from this analysis showed no plants that were homozygous for any of the insertions (Table 2); the deviation from the ratios of progeny expected if the T-DNA insertions were segregating as typical Mendelian characters was significant below a significance level of 1×10^−4^. The lack of any progeny homozygous for any of the T-DNA insertions suggests that the inactivation of each gene is lethal. Further analysis of the progeny of insertion lines corresponding to the PAPS1, PAPS2, and PAPS4 genes all showed ratios of heterozygous to wild-type close to 2∶1 but significantly different from 1∶1 (Table 2), indicative of a typical Mendelian character homozygous mutants of which are not viable. On the other hand, lines with T-DNA insertions in the PAPS3 gene showed ratios different from 2∶1 at significance levels of 0.06 and 0.17 for the two insertions. In contrast, the results for these two lines were not statistically different from an expected ratio of 1∶1. This ratio has been shown to be indicative of a gametophyte lethal mutation [16]. Together, these results indicate that all four *Arabidopsis* poly(A) polymerase genes are essential for some aspect of growth and development, and implicate one poly(A) polymerase gene (encoded by At3g06560) in gametogenesis.

**Table 2:** Results of genotyping of progeny of insertion mutants.

Gene designation^1^	Insertion designation	ht^2^	wt^3^	Ratio^4^	χ^2^ (2∶1)^5^	χ^2^ (1∶1)^6^
At1g17980	WiscDsLox4-13-4 16L14	36	22	1.6	0.704^*^	0.401^*^
	WiscDsLox441G5	36	19	1.9	0.008^*^	0.774^*^
						
At2g25850	SALK_083263	42	25	1.7	0.069^*^	0.435^*^
						
At3g06560	SALK_105192	19	17	1.1	0.111^**^	0.739^**^
	SALK_133557	22	17	1.3	0.641^**^	0.423^**^
						
At4g32850	SALK_063790	32	17	1.9	0.093^*^	0.761^*^
	SALK_007979	43	25	1.7	0.263^*^	0.608^*^

1 *Arabidopsis* gene designation corresponding to the PAP gene of interest.

2 numbers of heterozygous individuals in the tested population.

3 numbers of wild-type individuals in the tested population.

4 ratio of heterozygous: wild-type plants in the tested population.

5 results of χ^2^ tests for the fit of the ratio to a predicted ratio of 2.

6 results of χ^2^ tests for the fit of the ratio to a predicted ratio of 1.

value calculated for the segregation ratio 2∶1^*^ or 1∶1^**^; (c): Calculated P value based on X^2^. P = 0.05 was chosen as a critical limit, such that the predicted ratio was not rejected for P values >0.05. * indicates a significant ratio of heterozygous to wild-type plants in a 2∶1 (^*^) or 1∶1 (^**^) ratio.

### Expression Characteristics of the Arabidopsis Poly(A) Polymerase Gene Promoters

**Figure 4:**
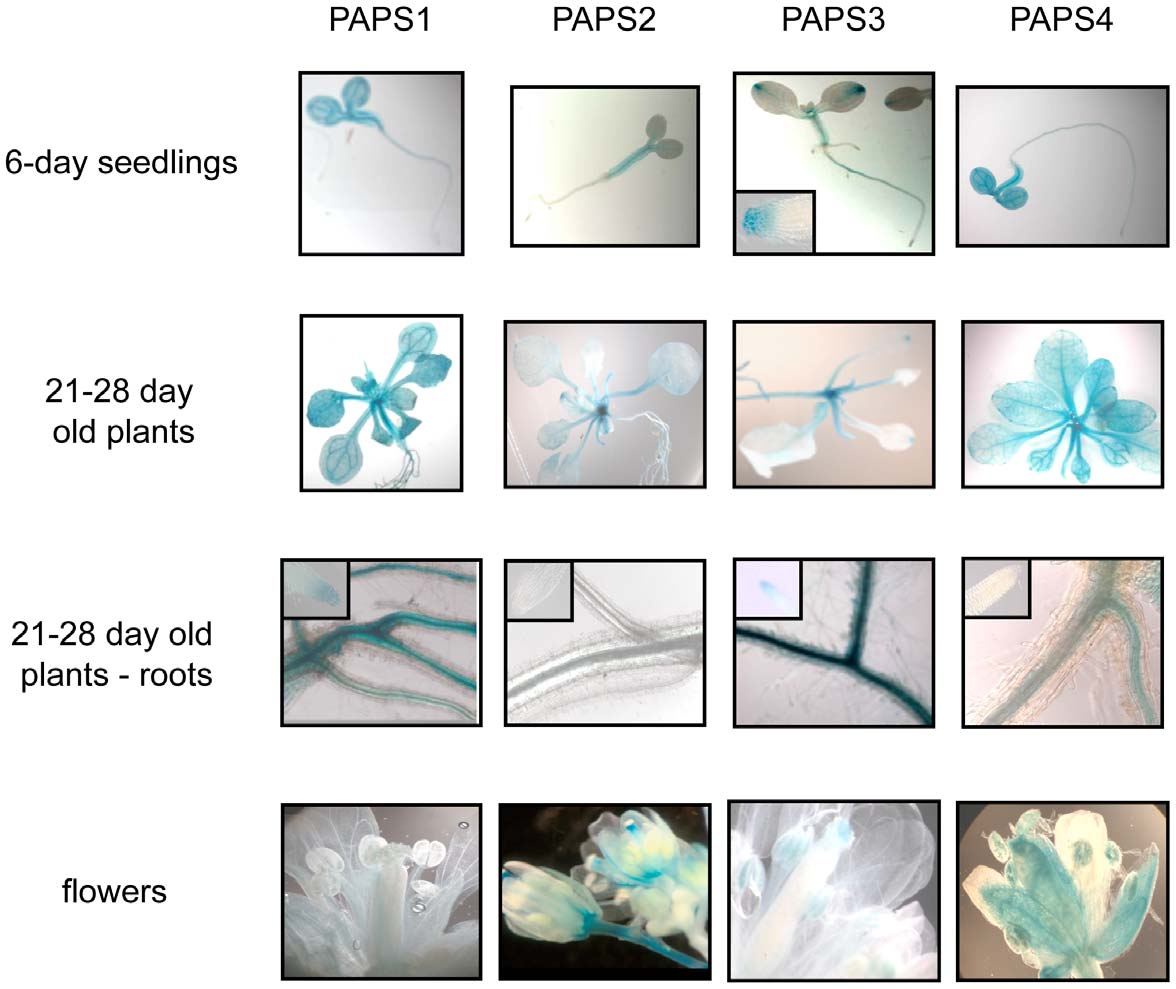
Profiles of *Arabidopsis* poly(A) polymerase promoter activity. The promoters from each of the four *Arabidopsis* poly(A) polymerase genes were fused to the GUS coding region of pCAMBIA1303 and the resulting transgenes introduced into *Arabidopsis*, all as described in Methods. After the times indicated on the left, plants were sampled and GUS activity determined using histochemical staining. Each column shows representative results from plants containing the promoter construct indicated at the top of the column.

One possible explanation for the essential nature of the four *Arabidopsis* poly(A) polymerase genes is that these encoded proteins all possess similar activities, but that they are expressed in a mutually-exclusive fashion, such that only one isoform is present at any time during growth and development. To explore this possibility, the activities of the four *Arabidopsis* poly(A) polymerase promoters were examined, using promoter-GUS fusions. Expression was monitored at different stages of growth using a standard histochemical stain. Representative results are presented in Figure 4 and are summarized in the following.

In seedlings six days after germination, the expression patterns of PAPS1 and PAPS4 promoters were almost identical with GUS being expressed throughout the cotyledons and hypocotyls but confined to the vascular tissue in the radicle. The activity of the PAPS2 promoter was confined largely to the hypocotyls. The PAPS3 promoter showed the most unique pattern, being most active in the cotyledon tips and vascular tissue of the radicle. Interestingly, the PAPS3 promoter was the only one active in the radicle tip (inlay of the PAPS3 panel showing the 6-day seedling).

All four poly(A) polymerase promoters were active in the rosette leaves of 3/4-week-old plants (Figure 4, second row). The PAPS1 and PAPS4 promoters were active throughout the leaves, especially in the vascular tissue and leaf petioles. The PAPS2 promoter was primarily active in the leaf petioles, but showed weak activity in the leaf vascular system. The PAPS3 promoter was most active in the petioles of the young leaves and at the leaf tips.

The PAPS1, PAPS3, and PAPS4 promoters were active in the primary and secondary root systems of 3–4 week-old plants (Figure 4, third row). The promoters from the PAPS1 and PAPS4 genes showed very similar patterns, appearing to be confined to the vascular system. The PAPS1 promoter also showed weak activity in the root tips (Figure 4, third row, PAPS1 inlay). The PAPS3 promoter was active throughout the root tissue excluding the elongation zone, and was quite strong in the root tip itself (Figure 4, third row, PAPS3 inlay). The PAPS2 promoter was not active to a noticeable degree in the roots or root tips.

The PAPS1 promoter showed very low activity in flowers (Figure 4, fourth row). A quite diverse expression pattern was observed among the various promoters in flowers (Figure 4, fourth row). The PAPS2 promoter was highly active in the style, receptacle and pedicel, and weakly active in the vasculature of sepals (Figure 4, fourth row). The activity of the PAPS3 promoter was restricted to the stigma and the pollen in mature anthers (Figure 4, fourth row). The PAPS4 promoter was very active in pollen, sepals, styles, and stigmas (Figure 4, fourth row).

To summarize these results, the PAPS1 and PAPS4 promoters possessed very similar activity profiles apart from the flower (that was largely devoid of PAPS1 promoter activity). The PAPS2 and PAPS3 promoters were more restricted in their activities, but these two promoters were active in tissues that also possessed active PAPS1 and PAPS4 promoters. The only obvious organ or tissue that showed any sort of poly(A) polymerase gene promoter exclusivity was the sepal, in which only the PAPS4 promoter was active. Thus, these results do not support the hypothesis that the essential nature of the four *Arabidopsis* poly(A) polymerase genes is due to mutually-exclusive patterns of gene expression.

### One of the Four Arabidopsis Poly(A) Polymerases Is Cytoplasmic

**Figure 5:**
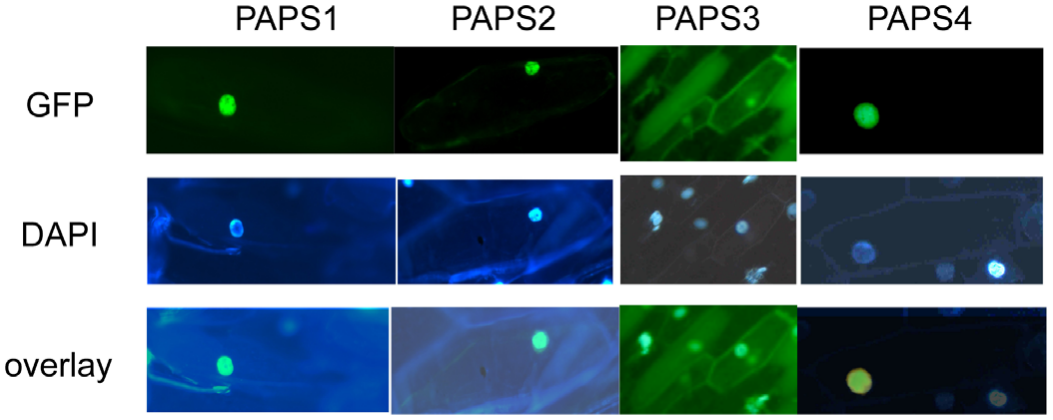
Subcellular localization of the four *Arabidopsis* poly(A) polymerases. Onion cells were bombarded with constructs encoding GFP-poly(A) polymerase fusion proteins and the distribution of the proteins recorded after 24–48 hrs. The top row shows the distributions of the GFP fusion proteins, the middle row the distributions of the DAPI stain, and the bottom row the merge of the GFP and DAPI images. The PAPS1, PAPS2, and PAPS4 images are about 50× magnifications, and the PAPS3 images 25× magnification.

Members of the PAPS3 family of plant poly(A) polymerases are smaller than the other poly(A) polymerases, lacking the extended C-termini that include putative nuclear localization sequences (Figure S1). This observation suggests that members of the PAPS3 protein family are cytoplasmic. To test this hypothesis, the subcellular distribution of the four *Arabidopsis* poly(A) polymerases was studied. For this, each protein was fused to GFP and the distribution of the fusion proteins in transiently transfected onion cells was studied. Representative results of such studies are given in Figure 5. As expected, the PAPS1, PAPS2, and PAPS4 fusion proteins were localized exclusively in the nuclei of transfected cells. However, the PAPS3-GFP fusion protein was found outside of the nucleus, either evenly-distributed throughout the cell or in diffuse extra-nuclear foci; an example of each pattern is shown in Figure 5. These results confirm the prediction arising from amino acid sequence analysis, and indicate that one of the four *Arabidopsis* poly(A) polymerases is cytoplasmic.

## Discussion

**Figure 6:**
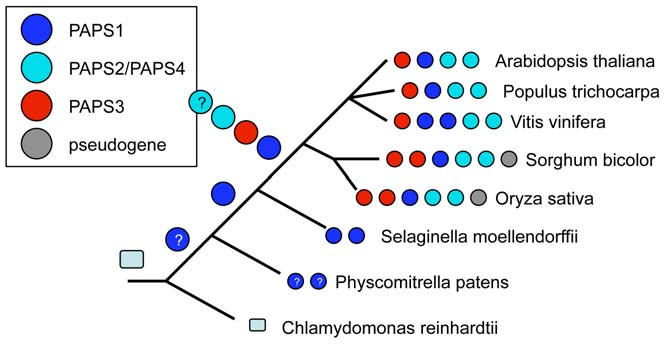
A model for the evolutionary history of plant poly(A) polymerases. The hypothetical poly(A) polymerase gene in the common ancestor of the lineages shown here and other eukaryotes is depicted with a light blue rectangular box; that the *Chlamydomonas* gene may be similar to this is indicated as the *Chlamydomonas* lineage retaining this gene. Distinctly plant poly(A) polymerases are represented with circles, with the relationships with the four *Arabidopsis* poly(A) polymerases indicated according to the colors shown at the upper left. The properties of the various poly(A) polymerase gene families are shown beneath the timeline, and the times of occurrences of putatiuve duplications above the timeline. The question marks in the *P. patens* genes indicates some uncertainty as to the relation ships of these genes to the hypothetical common ancestor of the genes in the rest of the plant lineage; this uncertainty owes to the absence of introns in these genes, and raises similar questions about the ancestral gene. Pseudogenes are represented as gray circles.

The nature of the plant poly(A) polymerase genes described in this report permits the construction of an interesting evolutionary history for poly(A) polymerases in the plant lineage (Figure 6). With the exception of two putative pseudogenes (Os04g49870 and Sb06g026810) in the grasses, all of the plant poly(A) polymerases appear to be derived from a single ancestral gene; this conclusion follows from the highly-conserved intron/exon organization of all of these genes, an organization that is shared by the poly(A) polymerase genes in *Selaginella*. The relationships between the *Physcomitrella* poly(A) polymerase genes and other plant poly(A) polymerase genes is not entirely clear, since the *Physcomitrella* genes lack intervening sequences and thus cannot be compared as can the other plant genes. However, the sequence analysis summarized in Figure 1 suggests that the *Physcomitrella* poly(A) polymerases are close relatives of one of the two *Selaginalla* poly(A) polymerases, and that all four are members of the PAPS1 family of poly(A) polymerases. Thus, it is likely that the ancestral plant poly(A) polymerase gene arose before the divergence of the *Physcomitrella* lineage from the other higher plant lines.

At some point in time after the divergence of the *Selaginella* and higher plant lineages, a series of further duplications gave rise to the three families of poly(A) polymerases seen in the angiosperms. The three basic families seem to have been established before the divergence of the angiosperm lineages studied here, but a number of subsequent duplications occurred subsequent to these various divergences. Thus, the PAPS3 family of the grasses expanded by duplication, apparently before the divergence of the sorghum and rice lineages. This is suggested by the closer similarity of the two rice poly(A) polymerases to their putative sorghum relatives than to each other. A duplication specific for the *Vitis* lineage seems to have given rise to an additional PAPS1 gene in this species.

The evolution of the PAPS2/PAPS4 family of plant poly(A) polymerases is more interesting, and harder to specify with certainty. Based on the topography of the tree shown in Figure 1, there appears to have been in the rosids a series of duplications of the putative ancestral PAPS2/PAPS4 gene that occurred after the divergence of the rosid species (*Arabidopsis thaliana*, *Populus trichocarpa*, and *Vitis vinifera*) studied in this report. An analogous duplication occurred prior to the divergence of the rice and sorghum lineages. Thus, it would appear as if the hypothetical ancestral PAPS2/PAPS4 gene was duplicated independently in most of the rosid lineages analyzed here. This is remarkable, since the PAPS2 and PAPS4 genes are both essential in *Arabidopsis*. The implication is that similar duplications occurred repeatedly, consistently, and independently in the course of plant evolution, and that these events have yielded poly(A) polymerases with different but essential functions.

However, the topography of the PAPS2/PAPS4 branch of the tree shown in Figure 1 may be a result, not of multiple independent duplications of the hypothetical ancestral gene in the various lineages, but rather of evolutionary trajectories that are constrained by the interactions of poly(A) polymerases with other proteins in the cell. One such constraint may be the interactions of these two proteins with Fip1 orthologs. Both PAPS2 and PAPS4 interact with one such ortholog, FIPS5, in *Arabidopsis* [12], [17]. Moreover, the PAPS4-FIPS5 interaction involves a part of FIPS5 that is highly divergent in plants [17]. Since FIPS5 is encoded by a single gene in *Arabidopsis* [12] and the other plants studied here (D. Xing et al., in preparation), any co-evolution of interacting FIPS5 and poly(A) polymerase domains could act to limit the diversification of different poly(A) polymerases. Should this be the case, then the ancestral PAPS2 and PAPS4 genes likely arose prior to the divergence of the different rosid lineages. This is rather different from the possibility suggested in the preceding paragraph. A clarification of these two models awaits further study.

The plant PAPS3 isoforms differ from the other poly(A) polymerase variants in that they lack the extended C-terminal domains seen in the latter proteins, along with the predicted nuclear localization information. Moreover, they are cytoplasmic in location, judging from the subcellular distribution of the *Arabidopsis* PAPS3-GFP fusion protein (Figure 5). In many ways, these smaller plant poly(A) polymerases resemble the mouse testis-specific poly(A) polymerase, TPAP[9]. TPAP is a cytoplasmic enzyme that is essential for spermatogenesis in mammals; TPAP-deficient mice display an arrest in spermatogenesis [8], a phenotype that is reversed by expression of TPAP as a transgene in deficient animals [18]. The expression studies performed in this work and earlier [11] do not provide an adequate resolution of the expression of the plant poly(A) polymerases during gametogenesis. However, public-domain microarray experiments (see, e.g., http://www.genevestigator.ethz.ch) indicate that the *Arabidopsis* PAPS3 gene is expressed preferentially during microgamete development and in mature pollen [12]. This possible parallel between TPAP and the plant PAPS3 family of proteins is interesting, as it suggests cytoplasmic poly(A) polymerases specific for sperm or pollen development evolved independently in the plant and animal lineages.

To summarize, the results presented here reveal a striking evolutionary history of poly(A) polymerase genes in plants. The plant poly(A) polymerase gene family expanded via a series of duplications, and the products of these duplications subsequently appear to have acquired specialized functions.

## Methods

### Plant Material

*Oryza sativa* sub-species *indica* var Lemont seed was a gift from Anna McClung, 93 Foundation, USDA-Texas A&M. Seed were germinated and plants cultivated in the greenhouse for 4–5 months till they set the seed. Plants were harvested before as well as during and after the flowering stage. Leaves, roots, stems, and flowers were used for genomic DNA and total RNA isolation. *Arabidopsis thaliana* ecotype Columbia was obtained from Lehle Seeds (Round Rock, TX) and used throughout this study. Seeds were germinated and plants cultivated in the greenhouse until maturity with a 16-h-light and 8-h-dark regime at 22°C.

### Identification, Isolation, and Analysis of Poly(A) Polymerase-Related cDNAs

Potential plant poly(A) polymerase-encoding genes were identified by searching the database available at Phytozome (http://www.phytozome.net/) using the TBLASTN algorithm [13] and the so-called PAPS4 or PAPS3 proteins (corresponding to the *Arabidopsis* At4g32850.1 and At3g06560.1 proteins, respectively) as queries; this search was performed January 2009 and was limited to the species listed in Table 1. Additional BLAST searches were performed to identify hypothetical proteins and to determine intron-exon organizations, where appropriate. The amino acid sequences of the proteins resulting from this search are provided in File S1. Initial amino acid sequence alignments were performed using ClustalX. More refined comparisons of the conserved core (Figure 1) were performed using the EXPRESSO analysis tool (http://www.tcoffee.org/Projects_home_page/expresso_home_page.html; [19]).

Rice cDNAs encoding putative poly(A) polymerases were isolated from total RNA by RT/PCR. Total RNA was isolated from *O. sativa* using Trizol (Invitrogen) per manufacturer's instructions. First strand cDNA was made with the ProSTAR™ Ultra HF RT-PCR system (Stratagene) using oligo-dT as a primer and otherwise following the manufacturer's specifications. For PCR amplification, 1.5 µl of the first strand reaction, 200 ng of primers (see Table S1 for the list of primers used in this study), 0.8 mM dNTPs, 5.0 µl of Ultra HF PCR buffer (Stratagene) and 2.5 units of PfuTurbo DNA polymerase (Stratagene) were used in 50 µl PCR reactions. Minus RT control reactions were done by synthesizing the first strand in the absence of StrataScript enzyme. PCR amplifications were run for 35 cycles of 92°C for 1 min, 55°C for 1 min and 72°C for 2 min.

PCR products were cloned into pBluescript or pGEM and the inserts sequenced; sequencing reactions were carried out with the BigDye terminator kit and analyzed on an ABI 310 Genetic Analyzer (Applied Biosystems). Sequence data were compiled using Vector NTI software (Informax).

### Northern Blot Analysis

Ten micrograms of total RNA was separated on 1.25% agarose-formaldehyde gels, transferred onto Immobilon N (Millipore) membranes by capillary transfer and hybridized overnight with ^32^P labeled probes (∼6.2×10^8^ cpm/ml) using sodium phosphate hybridization solution (0.12 M sodium phosphate pH 7.2, 0.25 M NaCl, 7% SDS, 1 mM EDTA) at 65°C. The probes used were specific to each poly A polymerase (see Table S1 for a list of primers used to make these probes). The filters were washed once with 2X SSC for 10 min at room temp, and twice with 0.1X SSC, 0.1% SDS at 65°C for 20 min. The washed filters were exposed to a phosphorimager screen and developed after 7–8 days.

### Genotyping of Insertion Mutants

Seed pools of T-DNA-mutagenized *Arabidopsis thaliana* were acquired from the Arabidopsis Biological Resource Center (Columbus, OH) or from the Sussman and Amasino laboratories at the University of Wisconsin-Madison. The mutant lines were allowed to self-pollinate and T2 seeds were harvested and germinated in soil, in the greenhouse, under normal long-day growth conditions. Genotyping for T-DNA mutants was performed on at least 35 T2 plants from each transgenic T-DNA line using a PCR based method. Gene-specific and T-DNA specific oligonucleotide primer sets (see Table S1) were designed to determine if plants were homozygous wild type, homozygous mutant or heterozygous. DNA was extracted from leaves taken from 3–4 week old, soil grown plants using a rapid homogenization plant DNA extraction kit (Caragen) with the following modified protocol. 200 µl DNA lysis buffer (100 mM Tris-HCL, pH 8.0; 50 mM EDTA, pH 8.0; 500 mM NaCl) was added to 100 mg leaf tissue and homogenized in the provided homogenizer or with mortar and pestle then centrifuged 30 seconds at <10,000 RPM. An additional 280 µl DNA lysis buffer was added along with 37.5 µl 20% SDS. The sample was placed in a 65°C water bath for 10 minutes. 94 µl 5M KAc was added and the sample was placed on ice for 5 minutes. The samples were then centrifuged at >13,000 RPM for 5 minutes after which the supernatant was transferred to a clean 1.5 ml Eppendorf tube. 600 µl phenol/chloroform (1∶1) was added and the samples were centrifuged 5 minutes at 12,000 RPM. The supernatant was removed and 360 µl isopropyl alcohol was added. The samples were centrifuged 10 minutes at >13,000 RPM and the pellet was washed with 70% EtOH and allowed to air dry. Finally, the pellet was resuspended in 30 µl of water. For PCR amplification, 25–50 ng of genomic DNA, 100 ng of each primer, 2.5 µl of 50 mM MgCl_2_, 5 µl of 2.5 mM dNTPs, 5 µl of 10X PCR buffer (Gibco/BRL) and 0.2 units of Taq DNA polymerase (Gibco/BRL) were used in 50 µl PCR reactions. PCR amplifications were run for 35 cycles of 92°C for 1 minute, 55°C for 1 minute and 72°C for 2 minutes.

### Analysis of PAPS Promoter Activity Using GUS Fusions

To analyze promoter activity the nucleotide sequence between the ATG start codon and the coding regions of the adjacent upstream gene for each of the four *Arabidopsis* poly(A) polymerase genes were amplified by PCR using the primers indicated Table S1 and *Arabidopsis* genomic DNA as a template. PCR products were subcloned sequentially into pGEM and then pCAMBIA1303. pGEM clones were sequenced before moving the promoter fragments into pCAMBIA1303. The promoter fragments were excised from pGEM with *Sal*I and *Nco*I and cloned into *Sal*I and *Nco*I digested pCAMBIA1303 vector. The sizes of the promoters were: PAPS1 - 734 bp; PAPS2 -781 bp; PAPS3 - 2111 bp; and PAPS4 -1041 bp.

Expression constructs were transferred to *Agrobacterium tumefaciens* strain GV-3850 and the helper plasmid PRK-2013 by tri-parental mating as described [20]. *Arabidopsis thaliana* plants, ecotype Columbia (COL), were transformed using the floral dip method [21]. Putative transformants were identified by plating T0 seed on germination medium containing 25-mg/l hygromyocin. At least five independent homozygous T2 lines for each construct were examined for GUS expression. Homozygous lines were identified by determining ratios of selective marker inheritance in T3 plants on hygromyocin-containing media.

Histochemical analysis of GUS activity in transgenic plants was performed essentially as described by Stomp [22]. Plant tissues were incubated at 37°C for 24 h in a 100 mM sodium phosphate buffer (pH 7.2, 0.5 mM potassium ferrocyanide, 0.5 mM potassium ferricyanide, 0.1% Triton X-100) containing 0.1–1 mM 5-bromo-4-chloro-3-indoyl glucuronide. Subsequently, the samples were then transferred to 70% ethanol to remove the chlorophyll.

Zeiss Stemi SV11 and Zeiss Axioplan 2 microscopes were used for visualization. Photographs were taken using a Zeiss Axiocam MRc5 and visualized using AxioVision 4.1 software (Zeiss, Jena, Germany). Images were processed using Adobe ImageReady software (version 2.0; Adobe Systems, San Jose, CA).

### Subcellular Distributions of Arabidopsis Poly(A) Polymerases

To determine the subcellular localization of poly(A) polymerases, the full-length proteins were fused to the GFP coding region in the pGDG plasmid [23]. For this, the coding regions of the *Arabidopsis* genes encoding PAPS1, PAPS2, and PAPS3 were amplified by PCR using first –strand cDNA as a template and the primers listed in Table S1. The PAPS4-GFP clone was kindly donated by Kevin Forbes. PCR products were subcloned into pGEM-T Easy (Promega) per the manufacturer's instructions, and resulting clones were sequenced as described above. pGEM-PAP clones were then digested with Sal I and Apa I for PAPS1, Bgl II for PAPS2, and Sal I and Bam HI for PAPS3, and the resulting fragments were ligated appropriately-digested digested pGDG. Recombinants were sequenced before use.

Plasmids encoding GFP fusion proteins were introduced into onion epidermal skin cells by particle bombardment using a PDS1000 DuPont Bio-Rad Microprojectile delivery system (Bio-Rad Laboratories). Briefly, for each sampole, 0.5 mg of gold microcarriers (1 µm) were vortexed vigorously in 1 ml 70% ethanol (V/V) for 3–5 minutes and then allowed to soak for 15 minutes. Microparticles were pelleted, ethanol removed, the particles washed three times in 1 ml sterile water, and then resuspended in 15 µl sterile water. To this, 2 µg of DNA, 50 µl 2.5 M CaCl_2_ and 20 µl 0.1 M spermidine were added with constant vortexing. Vortexing was continued for 3 minutes. Microparticles were pelleted in a microfuge for 2 seconds, the supernatant removed, and the pellet washed with 140 µl of 70% ethanol, then 140 µl of 100% ethanol and finally resuspended in 12 µl of 100% ethanol.

For macrocarrier preparation, suspended microcarriers were spread in the center of macrocarrier (Biorad Labs, USA) and installed in the particle gun assembly per the manufacturer's instructions. For all experiments, a helium pressure of 1100 psi was selected. The distance between rupture disk and macrocarrier was adjusted to 8–10 cm from the onion tissue. Following bombardment, the tissue was transferred to T- agar media, incubated at 25°C and then analyzed 24–48 hours after bombardment.

To locate DNA, transfected cells were stained with 2.5 µg/ml 4′,6-diamidino-2-phenylindole (DAPI) and 0.5% Triton X-100 in phosphate buffer saline (PBS) for 30 min at room temperature.

Localization of GFP and DsRed expression in onion cells was determined using a Zeiss Stemi SV11 microscope with a Zeisss AttoArc 2 light source. Excitation and emission wavelengths for GFP, were 470 nm and 500 nm, respectively, and for DAPI, 358 nm and 461 nm, respectively. Photographs were taken using a Zeiss Axiocam MRc5 and visualized using AxioVision 4.1 software (Zeiss, Jena, Germany). Images were processed using Adobe ImageReady software (version 2.0; Adobe Systems, San Jose, CA).

## Supporting Information

Figure S1 Global alignment of poly(A) polymerases. Sequences used for this alignment are given in File S1. Alignments were executed using the current version of the CLC Workbench suite of sequence analysis tools. In the display, deeper shades of red indicate more dissimilarity, and deeper shades of blue greater sequence similarity. A graphical depiction of sequence conservation is shown on the last line of the alignments.
(4.72 MB PDF)

Figure S2 Intron-exon organization of plant and mammalian poly(A) polymerase genes. The conserved “core” PAP sequences (File S2) were aligned and the alignment saved in the CLUSTAL format. Individual amino acid sequences were aligned to genome nucleotide databases and the output used to determine the positions of introns. These were added to the CLUSTAL alignment in the form of shading of the two amino acids that bound the intron positions. Green shading denotes introns in plant genes and blue shading the positions of introns in mammalian genes.
(0.10 MB DOC)

Table S1 Primers and plasmids used in this study
(0.10 MB DOC)

File S1 FASTA file of poly(A) polymerase sequences used in this study.
(0.03 MB TXT)

File S2 FASTA file of the sequences of the poly(A) polymerase “core” (see the text) used in Figures 1 and 2.
(0.02 MB TXT)
