# Supplementary material for: Characterization of Genes Encoding Poly(A) Polymerases in Plants: Evidence for Duplication and Functional Specialization
Source: PLoS One. 2009 Nov 26;4(11):e8082. doi: 10.1371/journal.pone.0008082 (PMC2778134; doi:10.1371/journal.pone.0008082)

```
                                              20
                                               |
At2g25850              M--------- ---------- -------VST  4
At4g32850              M--------- ---------- ------MVGT  5
PoplarXVIII            M--------- ---------- -------VGS  4
PoplarVI               M--------- ---------- -------VGS  4
GSVIVT00034292001      M--------- ---------- -------VSS  4
Os06g36360             M--------- ---------- ------AACN  5
Sb10g022090            M--------- ---------- ------AS--  3
Os02g13400             M--------- ---------- ------AGSF  5
Sb04g008100            M--------- ---------- ------AGSV  5
SelaginellaPAP1        M--------- ---------- ------AQSN  5
SelaginellaPAP2        M--------- ---------- ----------  1
GSVIVT00017746001      M--------- ---------- ------SNLG  5
PoplarXV               ---------- ---------- ----------  –
At1g17980              M--------- ---------- ------ASVQ  5
GSVIVT00030424001      M--------- ---------- ------ASVS  5
Os06g21470             M--------- ---------- ------AKS-  4
Sb01g012650            M--------- ---------- ------SKA-  4
GSVIVT00016654001      M--------- ---------- ----------  1
mossPAP1               M--------- ---------- ------EGS-  4
mossPAP2               M--------- ---------- ------DGD-  4
Os04g49870             M--------- ---------- ------A--S  3
Sb06g026810            M--------- ---------- ------AMAS  5
Dictyostelium          MNKNGGPPVA NITTSSTTIT STTTTQAKSQ  30
mouse                  M-PFPVTTQG SQQTQ----- --PPQ-----  17
human                  M-PFPVTTQG SQQTQ----- --PPQ-----  17
mouse                  MMPFAVTTQG AQQPA----- --PAP-----  18
human                  M-PFPVTTQG PPQPA----- --PPP-----  17
mouse                  MKEMSANTML DSQRQ----- ----Q-----  16
human                  MKEMSANTVL DSQRQ----- ----Q-----  16
Drosophila             MWNSEPTHRQ HHQHNGNSTS GGPPA-----  25
Caenorhabditis         M----SATEK DKTPL----- ----------  11
Chlamydomonas          MAVADPNDVY ---------- ----------  10
Schizosaccharomyces    MTT------- ---------- ----------  3
Saccharomyces          MSSQ------ ---------- ----------  4
Os03g19920             MSRSSRG--- ---------- --GRQSASSA  15
Sb01g037200            MALDLAV--- ---------- --G---GSAA  12
Os07g48890             MA-------- ---------- ----------  2
Sb02g043400            MAYMAAVAPV PWWPPPPELA PVGFPDASSP  30
At3g06560              MKKG------ ---------- ----------  4
GSVIVT00033174001      MAHAHRSQNS VALVHSQPLI HPQPPVVNAP  30
```

Conservation

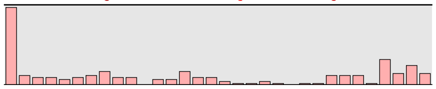

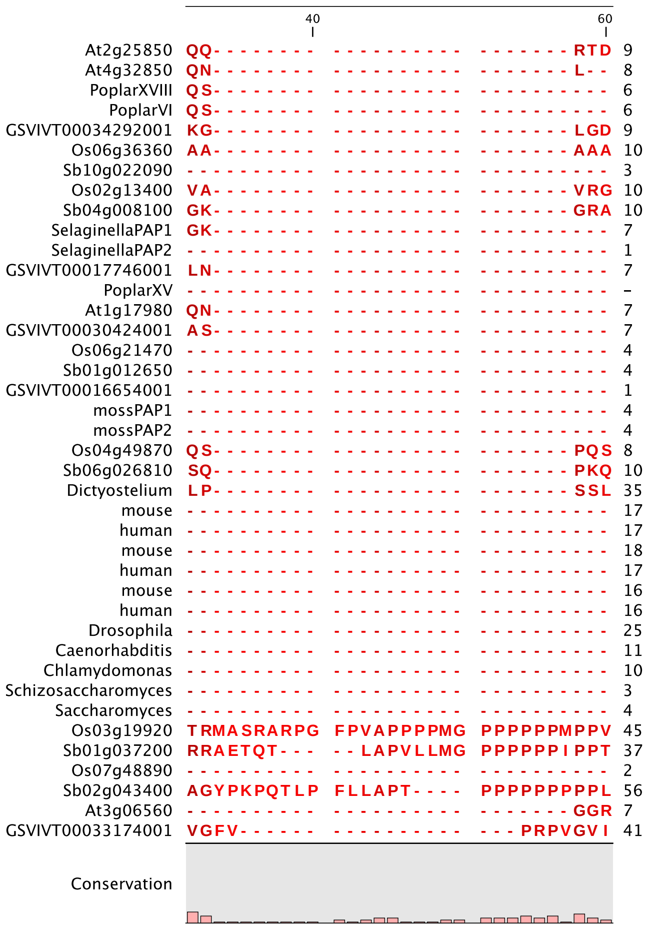
40
60
At2g25850 QQ- RTD 9
At4g32850 QN- L- - 8
PoplarXVIII QS- 6
PoplarVI QS- 6
GSVIVT00034292001 KG- LGD 9
Os06g36360 AA- AAA 10
Sb10g022090 - 3
Os02g13400 VA- VRG 10
Sb04g008100 GK- GRA 10
SelaginellaPAP1 GK- 7
SelaginellaPAP2 - 1
GSVIVT00017746001 LN- 7
PoplarXV -
At1g17980 QN- 7
GSVIVT00030424001 AS- 7
Os06g21470 - 4
Sb01g012650 - 4
GSVIVT00016654001 - 1
mossPAP1 - 4
mossPAP2 - 4
Os04g49870 QS- PQS 8
Sb06g026810 SQ- PKQ 10
Dictyostelium LP- SSL 35
mouse - 17
human - 17
mouse - 18
human - 17
mouse - 16
human - 16
Drosophila - 25
Caenorhabditis - 11
Chlamydomonas - 10
Schizosaccharomyces - 3
Saccharomyces - 4
Os03g19920 TRMASRARPG FPVAPPPPMG PPPPPPMPPV 45
Sb01g037200 RRAETQT- - - - - LAPVLLMG PPPPPPIPPT 37
Os07g48890 - 2
Sb02g043400 AGYPKPQTLP FLLAPT- - - - PPPPPPPPPL 56
At3g06560 - GGR 7
GSVIVT00033174001 VGFV- PRPVGVI 41
Conservation

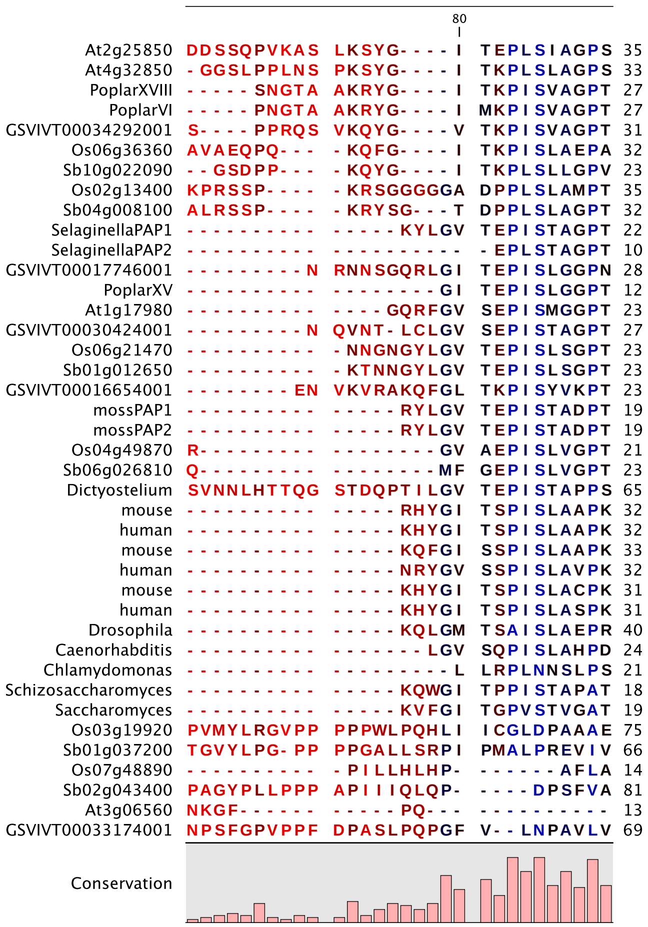

80
At2g25850 DDSSQPVKAS LKSYG----I TEPLSIAGPS 35
At4g32850 -GGSLPPLNS PKSYG----I TKPLSLAGPS 33
PoplarXVIII -----SNGTA AKRYG----I TKPISVAGPT 27
PoplarVI -----PNGTA AKRYG----I MKPISVAGPT 27
GSVIVT00034292001 S----PPRQS VKQYG----V TKPISVAGPT 31
Os06g36360 AVAEQPQ--- -KQFG----I TKPISLAEPA 32
Sb10g022090 --GSDPP--- -KQYG----I TKPLSLLGPV 23
Os02g13400 KPRSSP---- -KRSGGGGGA DPPLSLAMPT 35
Sb04g008100 ALRSSP---- -KRYSG---T DPPLSLAGPT 32
SelaginellaPAP1 ---------- -----KYLGV TEPISTAGPT 22
SelaginellaPAP2 ---------- ---------- -EPLSTAGPT 10
GSVIVT00017746001 ---------N RNNSGQRLGI TEPISLGGPN 28
PoplarXV ---------- --------GI TEPISLGGPT 12
At1g17980 ---------- ----GQRFGV SEPISMGGPT 23
GSVIVT00030424001 ---------N QVNT-LCLGV SEPISTAGPT 27
Os06g21470 ---------- -NNGNGYLGV TEPISLSGPT 23
Sb01g012650 ---------- -KTNNGYLGV TEPISLSGPT 23
GSVIVT00016654001 --------EN VKVRAKQFGL TKPISYVKPT 23
mossPAP1 ---------- -----RYLGV TEPISTADPT 19
mossPAP2 ---------- -----RYLGV TEPISTADPT 19
Os04g49870 R--------- --------GV AEPISLVGPT 21
Sb06g026810 Q--------- --------MF GEPISLVGPT 23
Dictyostelium SVNNLHTTQG STDQPTILGV TEPISTAPPS 65
mouse ---------- -----RHYGI TSPISLAAPK 32
human ---------- -----KHYGI TSPISLAAPK 32
mouse ---------- -----KQFGI SSPISLAAPK 33
human ---------- -----NRYGV SSPISLAVPK 32
mouse ---------- -----KHYGI TSPISLACPK 31
human ---------- -----KHYGI TSPISLASPK 31
Drosophila ---------- -----KQLGM TSAISLAEPR 40
Caenorhabditis ---------- -------LGV SQPISLAHPD 24
Chlamydomonas ---------- ---------L LRPLNNSLPS 21
Schizosaccharomyces ---------- -----KQWGI TPPISTAPAT 18
Saccharomyces ---------- -----KVFGI TGPVSTVGAT 19
Os03g19920 PVMYLRGVPP PPPWLPQHLI ICGLDPAAAE 75
Sb01g037200 TGVYLPG-PP PPGALLSRPI PMALPREVIV 66
Os07g48890 ---------- -PILLHLHP- ------AFLA 14
Sb02g043400 PAGYPLLPPP APIIIQLQP- ----DPSFVA 81
At3g06560 NKGF------ -----PQ--- ---------- 13
GSVIVT00033174001 NPSFGPVPPF DPASLPQPGF V--LNPAVLV 69
Conservation

	100		120	
At2g25850	AADVKRNLEL	EKFLVDEGLY	ESKEETMRRE	65
At4g32850	SADIKRNVEL	EKYLVDEGLY	ESKDDTMRRE	63
PoplarXVIII	EPDLHRNAEL	EKFLVDSGLN	ESKDETIKRE	57
PoplarVI	EPDLHRNAEL	EK--QRRGYE	ERRCSGPYRS	55
GSVIVT00034292001	EVDIQRSLEL	EKFLVDAGLY	ESKEEAIKRA	61
Os06g36360	EVDLQKTAEL	EKFLVEAGLY	ESPEESARRE	62
Sb10g022090	EADLQRTAEL	EKFLVEAGLY	ESPDESARRE	53
Os02g13400	VADLHKTAEL	EKFLVEAGLY	EGEEESAKRE	65
Sb04g008100	LADLQRTAEL	EKFLVEAGLY	EGKEESAKRE	62
SelaginellaPAP1	EADFARTRDL	EKVLTEAGLY	ESPEEAVQRE	52
SelaginellaPAP2	PADLGRTRDL	EKLLSNAGLN	ESREEAMKRE	40
GSVIVT00017746001	ELDVTKTQEL	EKFLAAAGLY	ESQEEAVSRE	58
PoplarXV	EYDVTKTREL	EKFLQDAGLY	ESQEEAVSRE	42
At1g17980	EFDVIKTREL	EKHLQDVGLY	ESKEEAVRRE	53
GSVIVT00030424001	EFDLIKTREL	EKFLADSGLY	ETREEAIRRE	57
Os06g21470	EKDVVRTQEV	EKCLADAGLY	ESQEEAVSRE	53
Sb01g012650	DKDLMQTTEV	EKYLSDAGLY	ESQDEAVLRE	53
GSVIVT00016654001	DFHIRRSFEL	EKVLWDGGVY	QVEEEARKRE	53
mossPAP1	EVDYVNTKQL	EECVKGLALQ	GSREEEVRRE	49
mossPAP2	EVDYANTKQL	EEFVKGLALQ	GSREEEVRRE	49
Os04g49870	PADLESTARL	ERLLREEGLY	ESAEETAARE	51
Sb06g026810	PADLEATAEL	EKVLREAGMY	ESPQESAVRA	53
Dictyostelium	SIDFKLSTEL	ENTLISFNLF	ESPEESRKRE	95
mouse	ETDCLLTQKL	IETLKPFGVF	EEEEELQRRI	62
human	ETDCVLTQKL	IETLKPFGVF	EEEEELQRRI	62
mouse	DTDRELTQKL	IETLQPFGVF	EEEEELQRRI	63
human	ETDCLLTQRL	IETLRPFGVF	EEEEELQRRI	62
mouse	EIDHIYTQKL	IDAMKPFGVF	EDEEELNHRL	61
human	EIDHIYTQKL	IDAMKPFGVF	EDEEELNHRL	61
Drosophila	PEDLQRTDEL	RGSLEPYNVF	ESQDELNHRM	70
Caenorhabditis	SKDIAQTTLL	IETLKKFGSY	EPKEETEQRM	54
Chlamydomonas	AEDKRHSAEL	EQFLRDAGLY	EPDEDAYLRQ	51
Schizosaccharomyces	EQENALNTAL	INELKNQNLF	ESPAESEKRV	48
Saccharomyces	AAENKLNDSL	IQELKKEGSF	ETEQETANRV	49
Os03g19920	RTDAFRSKSL	LNFISRTGVL	PSPEEELKRQ	105
Sb01g037200	YMDECRSRSL	LKFISDAGIV	PSLEDERRRE	96
Os07g48890	QMDSRRTTSL	LQ---DEGGI	PSPEADKKRE	41
Sb02g043400	EVDQRRSSSL	VQFLKDEGAV	PSPEDEKKRE	111
At3g06560	--DDESSISL	RQLMVNEGLI	PSLEDEVKRR	41
GSVIVT00033174001	RMEHRRSISL	LQFMSNEGLI	PSPEEELKRK	99

Conservation

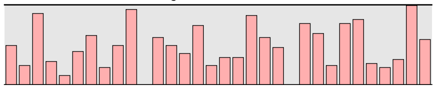

		140		
At2g25850	EVVVRIDQIV	KHWVKQLTRQ	RGYTDQMVED	95
At4g32850	EVLGRIDQIV	KHWVKQLTQQ	RGYTDQMVED	93
PoplarXVIII	EVLGRIDQIV	KDWVKQLTRQ	RGYTDQMVEE	87
PoplarVI	----------	KDWVKRLTRQ	RGYTDQMVEE	75
GSVIVT00034292001	EVLDRLGQIV	KDWVKQLTRL	RGYTDQMVED	91
Os06g36360	EVLGELDKIV	KDWVKQLTSQ	RGYTDQMVEE	92
Sb10g022090	EVLGKLDQIV	KDWVKQLTSQ	RGYTDQMVEE	83
Os02g13400	EVLREIDQIV	KEWVKKVTIQ	KGYSEQMVKE	95
Sb04g008100	DVLCEIGQIV	KEWVKQLTSK	KGYADQLVEQ	92
SelaginellaPAP1	EVLGRLDQLV	KEWVKNICLR	KGYSEQLTQE	82
SelaginellaPAP2	GVLGRLDQIV	KSWVRQICVN	KGFSNEVVQE	70
GSVIVT00017746001	EVLGRLDQIV	KIWVKAISRA	KGLNEQLVQE	88
PoplarXV	EVLGRLDQIV	KNWVKVISRA	KRLNEQLVQE	72
At1g17980	EVLGILDQIV	KTWIKTISRA	KGLNDQLLHE	83
GSVIVT00030424001	EVLGRVDQIV	KVWVKTVSRA	KGFNEQLVHE	87
Os06g21470	EVLGKLDQIV	KAWIKKATRA	SGFGDQFVQE	83
Sb01g012650	EVLGKLDQTV	KAWIKKATRI	SGYGEQFVHE	83
GSVIVT00016654001	EIIEKLRVVV	KSWVKQVTRW	KGYTDKMVEN	83
mossPAP1	EVLGRLDELV	NVWVKSMSRK	KGLNDEYVRE	79
mossPAP2	EVLGLLDELV	NVWVKMVSRM	KGLNDEYVRE	79
Os04g49870	EVLRGLRGVV	DRWVKRLTRQ	RGYPDGMADR	81
Sb06g026810	EVLRDLQGIV	DRWVKQLTLK	HGYPDAMVDE	83
Dictyostelium	EILGKLNQIV	REWAKQVSLK	KGYPEQTASE	125
mouse	LILGKLNNLV	KEWIREISES	KNLPQSVIEN	92
human	LILGKLNNLV	KEWIREISES	KNLPQSVIEN	92
mouse	LILQKLNNLV	KEWIREISES	RNLPQAVIEN	93
human	LVLEKLNNLV	KEWIREISES	KSLPQSVIEN	92
mouse	VVLGKLNNLV	KEWISDISES	KNLPPSVVAT	91
human	VVLGKLNNLV	KEWISDVSES	KNLPPSVVAT	91
Drosophila	EILAKLNTLV	KQWVKEISVS	KNMPESAAEK	100
Caenorhabditis	EVLRNLNRLV	KEWVKNVTAM	K-IPNGEGVN	83
Chlamydomonas	EVLGLFYELT	QTWVKGVCRK	KNLN---VED	78
Schizosaccharomyces	KVLDELQQIT	TEFVKKVSLA	KHMNEKMANE	78
Saccharomyces	QVLKILQELA	QRFVYEVSKK	KNMSDGMARD	79
Os03g19920	VVVRELDKIV	MGWAKRVAYD	QREQYW---N	132
Sb01g037200	RVVRELGKIV	MEWAKRVAYE	QGKQHW---I	123
Os07g48890	QVIRKLNKIV	MDWAKVVAYE	QRVPPR---R	68
Sb02g043400	KVIRELKKIV	MHWANAVAYE	QSVPQG---L	138
At3g06560	GVINQLRKIV	VRWVKNVAWQ	HRLPQNQIDA	71
GSVIVT00033174001	NVIEKLKEIV	LTWVKRVAWQ	RQRPKQQIAV	129

Conservation

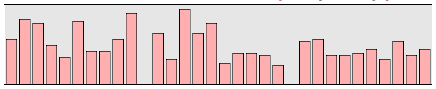

	160		180	
At2g25850	ANAVIFTFGS	YRLGVHGPMA	DIDTLCVGPS	125
At4g32850	ANAVIFTFGS	YRLGVHGPGA	DIDTLCVGPS	123
PoplarXVIII	ANAVIFTFGS	YRLGVHGPGA	DIDTLCVGPS	117
PoplarVI	ANAVIFTFGS	YRLGVHGPGA	DIDTLCVGPS	105
GSVIVT00034292001	ANAVLFTFGS	YRLGVHGPGT	DIDTLCIGPS	121
Os06g36360	ANAVLFTFGS	YRLGVHGPGA	DIDTLCVGPS	122
Sb10g022090	ANAVLFTFGS	YRLGVHGPGA	DIDTLCVGPS	113
Os02g13400	ANAVLFTFGS	YRLGVHGPGA	DIDALCIGPS	125
Sb04g008100	ANAVLFTFGS	YRLGVHGPEA	DIDTLCVGPS	122
SelaginellaPAP1	ANAKIFTFGS	YRLGVHGPGT	DIDTLCVGPR	112
SelaginellaPAP2	ANAKIFTFGS	YRLGVHGPGS	DIDTLCVGPS	100
GSVIVT00017746001	ANAKIFTFGS	YRLGVHGPGA	DIDTLCVGPR	118
PoplarXV	ANAKIFTFGS	YRLGVHGPGA	DIDTLCVGPR	102
At1g17980	ANAKIFTFGS	YRLGVHGPGA	DIDTLCVGPR	113
GSVIVT00030424001	ANAKIFTFGS	YRLGVHGPGA	DIDTLCVGPR	117
Os06g21470	ANAKIFTFGS	YRLGVHGPGA	DIDTLCVGPR	113
Sb01g012650	ANAKIFTFGS	YRLGVHGPGA	DIDTLCVGPR	113
GSVIVT00016654001	ANALIVTFGS	YRLGVHGPGS	DIDTLCIGPS	113
mossPAP1	ARCKIFTFGS	YRLGVHGPGA	DIDTLCVGPC	109
mossPAP2	ARCKIFTFGS	YRLGVHGPGA	DIDTLCVGPC	109
Os04g49870	ATALVLPFGS	YRLGVHGRGS	DIDALVVGPS	111
Sb06g026810	ATALLLPFGS	YRLGVHGGGS	DIDALVVGPS	113
Dictyostelium	VVAKIFTFGS	YRLGVHGPGS	DIDTLCVGPK	155
mouse	VGGKIFTFGS	YRLGVHTKGA	DIDALCVAPR	122
human	VGGKIFTFGS	YRLGVHTKGA	DIDALCVAPR	122
mouse	VGGKIFTFGS	YRLGVHTKGA	DIDALCVAPR	123
human	VGGKIFTFGS	YRLGVHTKGA	DIDALCVAPS	122
mouse	VGGKIFTFGS	YRLGVHTKGA	DIDALCVAPR	121
human	VGGKIFTFGS	YRLGVHTKGA	DIDALCVAPR	121
Drosophila	LGGKIYTFGS	YRLGVHHKGA	DIDALCVAPR	130
Caenorhabditis	AGGKLFTFGS	YRLGVHSSGA	DIDTLAVVPR	113
Chlamydomonas	ARAHVYTFGS	YRLGVHGPGA	DMDTLVVGPR	108
Schizosaccharomyces	AGGKIFTYGS	YRLGVYGPGS	DIDTLVVVPK	108
Saccharomyces	AGGKIFTYGS	YRLGVHGPGS	DIDTLVVVPK	109
Os03g19920	TTATVLTFGS	YALGAYGPES	DIDAVCVGPC	162
Sb01g037200	TSATVLTFGS	YALGAYGPES	DIDVLCIGPY	153
Os07g48890	ATATVLTYGS	YTLGAHGPES	DIDALCVGPC	98
Sb02g043400	ATATVLTYGS	YTLGAHGPES	DIDVLCVGPC	168
At3g06560	TNATILPYGS	YGLGVYGSES	DIDALCIGPF	101
GSVIVT00033174001	TSATILTYGS	YGLGVHGPES	DIDALCVGPF	159

Conservation

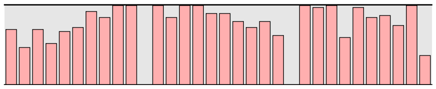

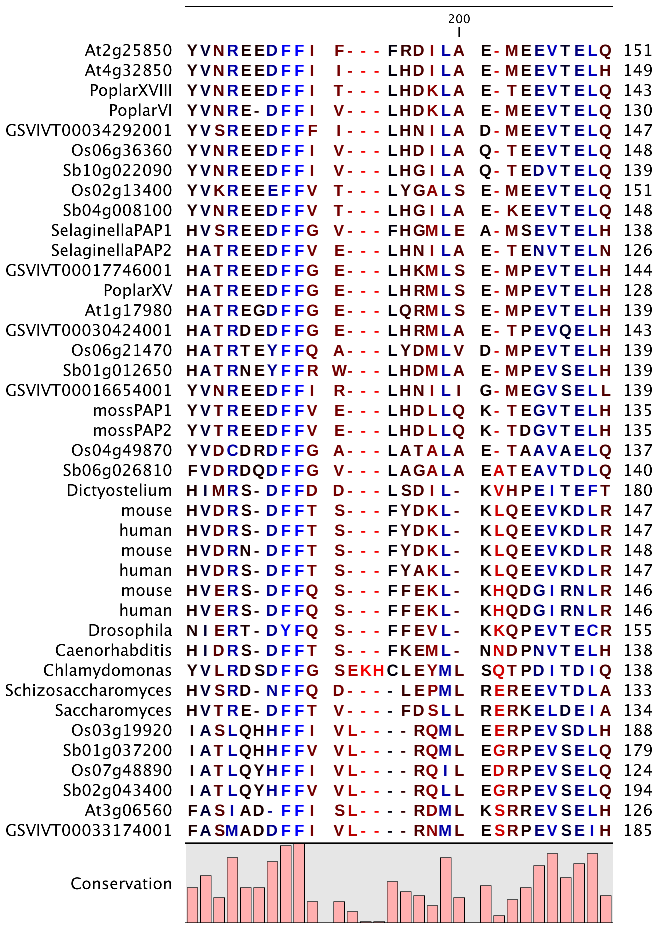
200
At2g25850 YVNREEDFFI F---FRDILA E-MEEVTELQ 151
At4g32850 YVNREEDFFI I---LHDILA E-MEEVTELH 149
PoplarXVIII YVNREEDFFI T---LHDKLA E-TEEVTELQ 143
PoplarVI YVNRE-DFFI V---LHDKLA E-MEEVTELQ 130
GSVIVT00034292001 YVSREEDFFF I---LHNILA D-MEEVTELQ 147
Os06g36360 YVNREEDFFI V---LHDILA Q-TEEVTELQ 148
Sb10g022090 YVNREEDFFI V---LHGILA Q-TEDVTELQ 139
Os02g13400 YVKREEEFFV T---LYGALS E-MEEVTELQ 151
Sb04g008100 YVNREEDFFV T---LHGILA E-KEEVTELQ 148
SelaginellaPAP1 HVSREEDFFG V---FHGMLE A-MSEVTELH 138
SelaginellaPAP2 HATREEDFFV E---LHNILA E-TENVTELN 126
GSVIVT00017746001 HATREEDFFG E---LHKMLS E-MPEVTELH 144
PoplarXV HATREEDFFG E---LHRMLS E-MPEVTELH 128
At1g17980 HATREGDFFG E---LQRMLS E-MPEVTELH 139
GSVIVT00030424001 HATRDEDFFG E---LHRMLA E-TPEVQELH 143
Os06g21470 HATRTEYFFQ A---LYDMLV D-MPEVTELH 139
Sb01g012650 HATRNEYFFR W---LHDMLA E-MPEVSELH 139
GSVIVT00016654001 YVNREEDFFI R---LHNILI G-MEGVSELL 139
mossPAP1 YVTREEDFFV E---LHDLLQ K-TEGVTELH 135
mossPAP2 YVTREEDFFV E---LHDLLQ K-TDGVTELH 135
Os04g49870 YVDCDRDFFG A---LATALA E-TAAVAELQ 137
Sb06g026810 FVDRDQDFFG V---LAGALA EATEAVTDLQ 140
Dictyostelium HIMRS-DFFD D---LSDIL- KVHPEITEFT 180
mouse HVDRS-DFFT S---FYDKL- KLQEEVKDLR 147
human HVDRS-DFFT S---FYDKL- KLQEEVKDLR 147
mouse HVDRN-DFFT S---FYDKL- KLQEEVKDLR 148
human HVDRS-DFFT S---FYAKL- KLQEEVKDLR 147
mouse HVERS-DFFQ S---FFEKL- KHQDGIRNLR 146
human HVERS-DFFQ S---FFEKL- KHQDGIRNLR 146
Drosophila NIERT-DYFQ S---FFEVL- KKQPEVTECR 155
Caenorhabditis HIDRS-DFFT S---FKEML- NNDPNVTELH 138
Chlamydomonas YVLRDSDFFG SEKHCLEYML SQTPDITDIQ 138
Schizosaccharomyces HVSRD-NFFQ D----LEPML REREEVTDLA 133
Saccharomyces HVTRE-DFFT V----FDSLL RERKELDEIA 134
Os03g19920 IASLQHHFFI VL----RQML EERPEVSDLH 188
Sb01g037200 IATLQHHFFV VL----RQML EGRPEVSELQ 179
Os07g48890 IATLQYHFFI VL----RQIL EDRPEVSELQ 124
Sb02g043400 IATLQYHFFV VL----RQLL EGRPEVSELQ 194
At3g06560 FASIAD-FFI SL----RDML KSRREVSELH 126
GSVIVT00033174001 FASMADDFFI VL----RNML ESRPEVSEIH 185
Conservation

Sequence	211–220	221–230	231–240	
At2g25850	PVTDAHVPVM	KFKFQGISID	LLYASISLLV	181
At4g32850	PVPDAHVPVM	KFKFQGIPID	LLYASISLLV	179
PoplarXVIII	PVPDAHVPVM	KFKFQGISID	LLYASISLLV	173
PoplarVI	PVPDAHVPVM	KFKFQGISID	LLYASISLLV	160
GSVIVT00034292001	PVPDAHVPVM	KFKFDGISID	LLYASISLLV	177
Os06g36360	PVPDAHVPVM	KFKFHGISID	LLYASVSLLV	178
Sb10g022090	PVPDAHVPVM	KFKFHGISID	LLYASVSLLV	169
Os02g13400	PVPDAHVPVM	KFKFRGLPID	LLYASVSLPV	181
Sb04g008100	PVPDAHVPVL	KFKFRGISID	LLYASLSLSV	178
SelaginellaPAP1	PVPDAHVPVM	RFKFSGISID	LLYAPLAVWT	168
SelaginellaPAP2	PVPEAHVPVM	KFKFDGISID	LLYARLSSWT	156
GSVIVT00017746001	PVPDAHVPVM	RFKFSGVSID	LLYAKLSLWV	174
PoplarXV	PVPDAHVPVM	RFKFKGVSID	LLYAKLSLWV	158
At1g17980	PVPDAHVPLM	GFKLNGVSID	LLYAQLPLWV	169
GSVIVT00030424001	PVPDAHVPVM	KFKFNGVSID	LLYARLSLWV	173
Os06g21470	PVPDAHVPVL	KFKLNGVSID	LLYANLTHVV	169
Sb01g012650	PVPDAHVPVL	GFKINGVSID	LLYANLAHAV	169
GSVIVT00016654001	PIPHAHVPVM	KFKFEGVSID	LLYASVSHLV	169
mossPAP1	TVPDAHVPVM	SFEFNGIPID	LLYARLPLWV	165
mossPAP2	TVPDAHVPVM	SFEFNGIPID	LLYARLPLWV	165
Os04g49870	PVPGAHVPVI	KMRFHGVQVD	LVYAGVCLPV	167
Sb06g026810	PVPGAHVPVM	KLRFRGVQVD	LVYASVNLPV	170
Dictyostelium	TVKDAFVPVI	TMVFSGIPID	LIYAKLALTA	210
mouse	AVEEAFVPVI	KLCFDGIEID	ILFARLALQT	177
human	AVEEAFVPVI	KLCFDGIEID	ILFARLALQT	177
mouse	AVEEAFVPVI	KLCFDGIEID	ILFARLALQT	178
human	AVEEAFVPVI	KLCFDGIEID	ILFARLALQT	177
mouse	AVEDAFVPVI	KFEFDGIEID	LVFARLAIQT	176
human	AVEDAFVPVI	KFEFDGIEID	LVFARLAIQT	176
Drosophila	SVEEAFVPVI	KMNFDGIEID	LLFARLSLKE	185
Caenorhabditis	GVEEAFVPVM	KLKYSGVELD	ILFARLALKE	168
Chlamydomonas	PVPDAFVPMI	GIKYKGVQID	ILYASLAMQT	168
Schizosaccharomyces	AVPDAYVPII	KFKFLGISID	LIFARLSVPR	163
Saccharomyces	PVPDAFVPII	KIKFSGISID	LICARLDQPQ	164
Os03g19920	SIENAKVPLM	RFKFNGMLVD	FPYVQLPVIN	218
Sb01g037200	SIEGAKVPLM	RFKFNGILVD	FPYVQLPFIN	209
Os07g48890	TVESAKVPLM	RFRFSGISVD	FTYAQLPAII	154
Sb02g043400	TIEKAKVPLM	RFRFTGIAVD	FTYAQLPVI-	223
At3g06560	CVKDAKVPLI	RFKFDGILVD	LPYAQLRVLS	156
GSVIVT00033174001	CVKDAKVPLM	RFKFDGISVD	LPYAQLKLLY	215

Conservation

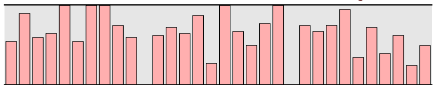

	260			
At2g25850	IPQDL-DISN	SSVLCDVDEQ	TVRSLNGCRV	210
At4g32850	VPQDL-DISS	SSVLCEVDEP	TVRSLNGCRV	208
PoplarXVIII	VPQDL-DISN	GSVLYEVDEQ	TVRSLNGCRV	202
PoplarVI	PEQDL-DISN	GSVLYEVDEQ	TVRSLNGCRV	189
GSVIVT00034292001	VPEDL-DISD	LSVLYNIDEP	TVRSLNGCRV	206
Os06g36360	VPPDL-DISQ	GSVLYDVDEV	TVRSLNGCRV	207
Sb10g022090	VPADL-DISQ	GSVLYDVDEA	TVRSLNGCRV	198
Os02g13400	IPPDF-DISQ	GSVLCDVDEA	TVRSLNGCRV	210
Sb04g008100	IPADF-DISQ	GSVLCDVDEA	TVRSLNGCRV	207
SelaginellaPAP1	IPEDL-DISQ	ESILRNLDEP	SVRSLNGCRV	197
SelaginellaPAP2	IPEDL-DISD	ESIMQNLDEQ	SVLSLNGCRV	185
GSVIVT00017746001	IPEDL-DVSQ	DSILQNADEQ	TVRSLNGCRV	203
PoplarXV	IPQDL-DVSQ	DSMLHNADEQ	TVRSLNGCRV	187
At1g17980	IPEDL-DLSQ	DSILQNADEQ	TVRSLNGCRV	198
GSVIVT00030424001	IPEDL-DISQ	ETILQNVDEQ	TVRSLNGCRV	202
Os06g21470	IPEDL-DLSH	DSILHNVDEQ	TVRSLNGCRV	198
Sb01g012650	IPEDL-DLSQ	DSILNNVDEQ	TVRSLNGCRV	198
GSVIVT00016654001	VPDDL-DISN	ESILYEADEP	TVRSLSGCRV	198
mossPAP1	IPEEL-DILQ	DTILQNVDEQ	SVRSLNGCRV	194
mossPAP2	IPEEL-DILQ	DSILQNVDEQ	SVRSLNGCRV	194
Os04g49870	VPGDL-DLSG	RSVLRGLDLA	TARSLNGVRV	196
Sb06g026810	VPRDL-DLSD	RAVLRGLDHV	TARSMNGVRV	199
Dictyostelium	IPEELNDLID	ESFLKNIDEK	SILSLNGCRV	240
mouse	IPEDL-DLRD	DSLLKNLDIR	CIRSLNGCRV	206
human	IPEDL-DLRD	DSLLKNLDIR	CIRSLNGCRV	206
mouse	IPEDL-DLRD	DSLLKNLDIR	CIRSLNGCRV	207
human	IPEDL-DLRD	DSLLKNLDIR	CIRSLNGCRV	206
mouse	ISDNL-DLRD	DSRLRSLDIR	CIRSLNGCRV	205
human	ISDNL-DLRD	DSRLRSLDIR	CIRSLNGCRV	205
Drosophila	IPDDF-DLRD	DNLLRNLDHR	SVRSLNGCRV	214
Caenorhabditis	VPDTQ-ELSD	DNLLRNLDQE	SVRSLNGCRV	197
Chlamydomonas	LPEQL-DLSN	HAVLRGCDEP	TVRALNGCRV	197
Schizosaccharomyces	VPRDL-ELSD	NNLLKGVEER	CVLSLNGTRV	192
Saccharomyces	VPLSL-TLSD	KNLLRNLDEK	DLRALNGTRV	193
Os03g19920	AAEAI-HAFD	PRLLAVVNEP	SWRCLSGVRV	247
Sb01g037200	AAEAM-HAFD	PHVLENVDGP	SWRCLSGVRA	238
Os07g48890	TS-------N	PHLLQKLDSL	SWRSLSGVRV	177
Sb02g043400	----------	-------DAL	----------	226
At3g06560	IPNNV-DVLN	PFFLRDIDET	SWKILSGVRA	185
GSVIVT00033174001	VPENL-NVLN	PYFLRNIDET	SWKSLSGVRA	244

Conservation

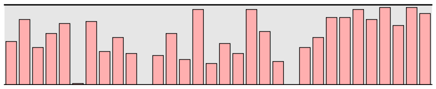

		280	300	
At2g25850	ADQILKLVPN	SEHFRTTLRC	LKYWAKKRGV	240
At4g32850	ADQILKLVPN	FEHFRTTLRC	LKYWAKKRGV	238
PoplarXVIII	ADQILKLVPN	VEHFRTTLRC	LKFWAKRRGV	232
PoplarVI	ADQILKLVPN	VEHFRATLRC	LKFWAKRRGV	219
GSVIVT00034292001	ADQILKLVPN	VEHFCTTLRC	LKFWAKRRGV	236
Os06g36360	ADQILRLVPN	VENFRTTLRC	LKYWAKKRGV	237
Sb10g022090	ADQIIRLVPN	IENFRTTLRC	LKYWAKRRGV	228
Os02g13400	ADQILRLVPN	AEIFRKTLRC	LKYWAQRRGV	240
Sb04g008100	ADQILRLVPN	VENFRTTLRC	LKYWAKRRGV	237
SelaginellaPAP1	TDQILRLVPN	IEHFRTTLRC	MKLWARKRGV	227
SelaginellaPAP2	TDQILRLVPN	IQHFRTTLRC	MKYWAKRRGV	215
GSVIVT00017746001	TDQILRLVPN	IQNFRTTLRF	MRFWAKRRGV	233
PoplarXV	TDQILRLVPN	IQNFRTTLRC	MRFWAKRRGV	217
At1g17980	TDQILRLVPN	IQNFRTTLRC	MRFWAKRRGV	228
GSVIVT00030424001	TDQILRLVPN	IQNFRTTLRC	MRFWAKRRGV	232
Os06g21470	TDKILRLVPN	ILTFRTTLRS	LRFWAKRRGV	228
Sb01g012650	TDQILRLVPN	ILSFRTTLRF	IRYWGKRRGV	228
GSVIVT00016654001	ADQILRLVPN	VEHFRTTLRC	LKFWAKRRGV	228
mossPAP1	TDRILRLVPN	MEHFRTTLRY	VKLWAKRRGV	224
mossPAP2	TDRILRLVPN	IEHFRTTLRY	VKLWAKRRGV	224
Os04g49870	ADEILRLVPD	ATAFRTTLRC	VKHWAKARGV	226
Sb06g026810	ADEILRLVPD	AAAFRTALRC	VKLWAKARGV	229
Dictyostelium	TDQILKLVPN	IPNFRMALRC	IKLWAIRRGI	270
mouse	TDEILHLVPN	IDNFRLTLRA	IKLWAKRHNI	236
human	TDEILHLVPN	IDNFRLTLRA	IKLWAKRHNI	236
mouse	TDEILHLVPN	IDSFRLTLRA	IKLWAKCHNI	237
human	TDEILHLVPN	IDNFRLTLRA	IKLWAKCHNI	236
mouse	TDEILHLVPN	KETFRLTLRA	VKLWAKRRGI	235
human	TDEILHLVPN	KETFRLTLRA	VKLWAKRRGI	235
Drosophila	TDEILALVPN	IENFRLALRT	IKLWAKKHGI	244
Caenorhabditis	AEQLLKLVPR	QKEFCVTLRA	IKLWAKNHGI	227
Chlamydomonas	TDTMLKLVPR	QEVFRTALRA	VKHWASLRGI	227
Schizosaccharomyces	TDQILQLVPN	RAVFKHALRA	IKFWAQRRAI	222
Saccharomyces	TDEILELVPK	PNVFRIALRA	IKLWAQRRAV	223
Os03g19920	NRQIMQLLPN	IKKFQILLRC	LKLWARKRGL	277
Sb01g037200	NRQIIQLVPN	MK--------	----------	250
Os07g48890	NEQIVQLVPN	AQKFQILLRC	IKLWAKRRGI	207
Sb02g043400	----------	-KKFQALLRC	IKLWARKRGL	245
At3g06560	NKCILQLVPS	LELFQSLLRC	VKLWAKRRGV	215
GSVIVT00033174001	NECILQLVPN	VENFQSILRC	IKLWAKRRGV	274

Conservation

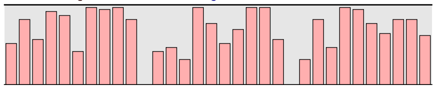

320

At2g25850	YSNVTGFLGG	VNWALLVARL	CQFYPNAIPS	270
At4g32850	YSNVTGFLGG	VNWALLVARV	CQLYPNAIPS	268
PoplarXVIII	YSNVTGFLGG	VNWALLVARV	CQLYPNAIPS	262
PoplarVI	YSNVTGFLGG	VNWALLVARV	CQLYPNAIPS	249
GSVIVT00034292001	YSNVTGFLGG	VNWALLVARV	CQLYPNAVPS	266
Os06g36360	YSNVTGFLGG	VNWALLVARV	CQLYPNAVPS	267
Sb10g022090	YSNVTGFLGG	VNWALLVARV	CQLYPNAVPS	258
Os02g13400	YSNVTGLLGG	VSWALLVARV	CQLYPNAVPS	270
Sb04g008100	YSNITGFLGG	VNWALLVARV	CQLYPNAVPS	267
SelaginellaPAP1	YSNVTGFLGG	VNWALLVARI	CQLYPNALPS	257
SelaginellaPAP2	YSNVTGFLGG	INWALLVARI	CQLYPNAVPS	245
GSVIVT00017746001	YSNVAGFLGG	INWALLVARI	CQLYPNALPS	263
PoplarXV	YSNVSGFLGG	INWALLVARI	CQLFPNALPN	247
At1g17980	YSNVSGFLGG	INWALLVARI	CQLYPNALPN	258
GSVIVT00030424001	YSNVSGFLGG	INWALLVARI	CQLYPNAVPS	262
Os06g21470	YSNVIGFLGG	INWALLVARI	CQLYPNASPS	258
Sb01g012650	YSNVMGFLGG	INWAILVGRI	CQLYPNASPS	258
GSVIVT00016654001	YSNVTGFLGG	VNLALLVARV	CQLYPNANPS	258
mossPAP1	YSNVIGFLGG	VNWALLVARI	CQLYPNAVPS	254
mossPAP2	YSNVIGFLGG	VNWALLVARI	CQLYPNAVPS	254
Os04g49870	YSNVAGFLGG	VGWAILVACV	CLLYPNASPS	256
Sb06g026810	YSNVSGFLGG	VAWAILVARV	CQLYPNAAPS	259
Dictyostelium	YSNILGFLGG	VSWALLTARI	CQLYPNSAPS	300
mouse	YSNILGFLGG	VSWAMLVART	CQLYPNAIAS	266
human	YSNILGFLGG	VSWAMLVART	CQLYPNAIAS	266
mouse	YSNILGFLGG	VSWAMLVART	CQLYPNAIAS	267
human	YSNILGFLGG	VSWAMLVART	CQLYPNAVAS	266
mouse	YSNMLGFLGG	VSWAMLVART	CQLYPNAAAS	265
human	YSNMLGFLGG	VSWAMLVART	CQLYPNAAAS	265
Drosophila	YSNSLGYFGG	VTWAMLVART	CQLYPNAAAA	274
Caenorhabditis	YSNSMGFFGG	ITWAILVARA	CQLYPNASPS	257
Chlamydomonas	SSNVTGYLGG	VNLAIMVAKI	CQLYPRAEAS	257
Schizosaccharomyces	YANVVGFPGG	VAWAMMVARI	CQLYPNAVSS	252
Saccharomyces	YANIFGFPGG	VAWAMLVARI	CQLYPNACSA	253
Os03g19920	HCHLLGFFAG	IHLAILAAFV	CIMHPHATLS	307
Sb01g037200	---LLGFFAG	IHLAILAAYV	CRRHPNASIN	277
Os07g48890	HCHLLGFFAG	IHLAILAAYV	CQRYPYGTIN	237
Sb02g043400	HCHYLGFFAG	IHLAILAAYV	CRKFPDASVN	275
At3g06560	YGNLNGFLGG	VHMAILAAFV	CGYQPNATLS	245
GSVIVT00033174001	YGNLFGYFGG	VHLAILAAFV	CQKNPHANLN	304

Conservation

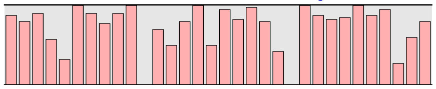

	331–340	341–350	351–360	
At2g25850	MLVSRFFRVY	TQWRWPNPVM	LCAIE-EDD-	298
At4g32850	MLVSRFFRVY	TQWRWPNPVM	LCAIE-EDE-	296
PoplarXVIII	MLVSRFFRVY	TQWRWPNPVM	LCSIE-EDD-	290
PoplarVI	MLVSRFFRVY	TQWRWPNPVM	LCSIE-EDA-	277
GSVIVT00034292001	MLVSRFFRVY	TQWRWPNPVM	LCAIE-EDE-	294
Os06g36360	MLVSRFFRVF	TQWQWPNPVM	LCAIE-EDE-	295
Sb10g022090	MLVSRFFRVF	TQWQWPNPVM	LCSIE-EDE-	286
Os02g13400	MLVSRFFRVF	TQWQWPNPVM	LCAIE-NDDN	299
Sb04g008100	MLVSRFFRVF	TQWQWPNPVM	LCAIE-NND-	295
SelaginellaPAP1	MLVSRFFRVY	TQWRWPNPVM	LCEIE-E-GS	285
SelaginellaPAP2	TLVSRFFRVY	TQWRWPNPVM	LCPIE-ERSS	274
GSVIVT00017746001	MLVSRFFRVY	TQWRWPNPVM	LCAIE-E-GT	291
PoplarXV	MLVSRFFRVY	TQWRWPNPVM	LCAIE-E-GS	275
At1g17980	ILVSRFFRVF	YQWNWPNAIF	LCSPD-E-GS	286
GSVIVT00030424001	TLVSRFFRVY	TQWRWPNPVM	LCPIE-E-KC	290
Os06g21470	MLISRFFKVY	SKWKWPNPVM	LCHIE-E-GS	286
Sb01g012650	MLISRFFRVY	SKWKWPNPVM	LCHIE-E-GY	286
GSVIVT00016654001	MLVSRFFRVY	TQWHWPNPVM	LCPIE-DKE-	286
mossPAP1	VLLSRFFRVY	KQWRWPNPVM	LCAIE-E-GP	282
mossPAP2	VLLSRFFRVY	KQWRWPNPVM	LCAIE-E-GS	282
Os04g49870	MLLPRFFRVF	ARWKWPSPVM	LRAIEHDDGE	286
Sb06g026810	MLVSRFFKVL	SQWKWPTPVM	LCDIEHDD-E	288
Dictyostelium	TIIHRFFKVY	EIWKWPAPIL	LCHIQ-EGGI	329
mouse	TLVHKFFLVF	SKWEWPNPVL	LKQPEECNLN	296
human	TLVHKFFLVF	SKWEWPNPVL	LKQPEECNLN	296
mouse	TLVRKFFLVF	SEWEWPNPVL	LKEPEERNLN	297
human	TLVRKFFLVF	SEWEWPNPVL	LKEPEERNLN	296
mouse	TLVHKFFLVF	SKWEWPNPVL	LKQPEESNLN	295
human	TLVHKFFLVF	SKWEWPNPVL	LKQPEESNLN	295
Drosophila	TLVHKFFLVF	SRWKWPNPVL	LKHPDNVNLR	304
Caenorhabditis	RLVHRMFFIF	STWTWPHPVV	LNEMNNDRND	287
Chlamydomonas	TVLLKFFILL	KAWPWPRAIH	LRIPEEHSLG	287
Schizosaccharomyces	VIVAKFFRIL	HQWNWPQPIL	LKPIEDGPLQ	282
Saccharomyces	VILNRFFIIL	SEWNWPQPVI	LKPIEDGPLQ	283
Os03g19920	SLFNSFFDIF	SHWHWPLPVS	LLDQPT----	333
Sb01g037200	TLLSLFFDIF	AHWPWPLPVS	LLDPPV----	303
Os07g48890	GLFTIFFDIF	AHWNWQIPVS	LHGQPT----	263
Sb02g043400	GLFAVFFQTF	AHWPWQVPDS	L---------	296
At3g06560	SLLANFFYTF	AHWQWPTPVV	LLEDTY----	271
GSVIVT00033174001	VLMSSFFKTF	SGWPWPTPVA	LEDGRL----	330

(Position ruler above the alignment: 340, 360)

Conservation

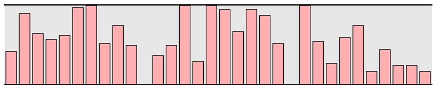

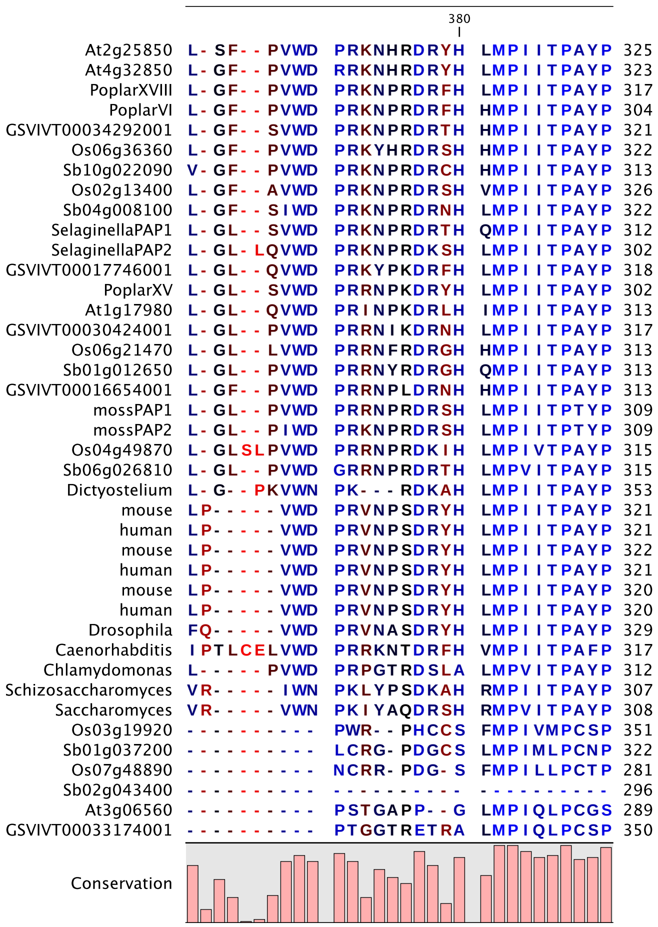
380
At2g25850 L-SF--PVWD PRKNHRDRYH LMPIITPAYP 325
At4g32850 L-GF--PVWD RRKNHRDRYH LMPIITPAYP 323
PoplarXVIII L-GF--PVWD PRKNPRDRFH LMPIITPAYP 317
PoplarVI L-GF--PVWD PRKNPRDRFH HMPIITPAYP 304
GSVIVT00034292001 L-GF--SVWD PRKNPRDRTH HMPIITPAYP 321
Os06g36360 L-GF--PVWD PRKYHRDRSH HMPIITPAYP 322
Sb10g022090 V-GF--PVWD PRKNPRDRCH HMPIITPAYP 313
Os02g13400 L-GF--AVWD PRKNPRDRSH VMPIITPAYP 326
Sb04g008100 L-GF--SIWD PRKNPRDRNH LMPIITPAYP 322
SelaginellaPAP1 L-GL--SVWD PRKNPRDRTH QMPIITPAYP 312
SelaginellaPAP2 L-GL-LQVWD PRKNPRDKSH LMPIITPAYP 302
GSVIVT00017746001 L-GL--QVWD PRKYPKDRFH LMPIITPAYP 318
PoplarXV L-GL--SVWD PRRNPKDRYH LMPIITPAYP 302
At1g17980 L-GL--QVWD PRINPKDRLH IMPIITPAYP 313
GSVIVT00030424001 L-GL--PVWD PRRNIKDRNH LMPIITPAYP 317
Os06g21470 L-GL--LVWD PRRNFRDRGH HMPIITPAYP 313
Sb01g012650 L-GL--PVWD PRRNYRDRGH QMPIITPAYP 313
GSVIVT00016654001 L-GF--PVWD PRRNPLDRNH HMPIITPAYP 313
mossPAP1 L-GL--PVWD PRRNPRDRSH LMPIITPTYP 309
mossPAP2 L-GL--PIWD PRKNPRDRSH LMPIITPTYP 309
Os04g49870 L-GLSLPVWD PRRNPRDKIH LMPIVTPAYP 315
Sb06g026810 L-GL--PVWD GRRNPRDRTH LMPVITPAYP 315
Dictyostelium L-G--PKVWN PK---RDKAH LMPIITPAYP 353
mouse LP----VWD PRVNPSDRYH LMPIITPAYP 321
human LP----VWD PRVNPSDRYH LMPIITPAYP 321
mouse LP----VWD PRVNPSDRYH LMPIITPAYP 322
human LP----VWD PRVNPSDRYH LMPIITPAYP 321
mouse LP----VWD PRVNPSDRYH LMPIITPAYP 320
human LP----VWD PRVNPSDRYH LMPIITPAYP 320
Drosophila FQ----VWD PRVNASDRYH LMPIITPAYP 329
Caenorhabditis IPTLCELVWD PRRKNTDRFH VMPIITPAFP 317
Chlamydomonas L-----PVWD PRPGTRDSLA LMPVITPAYP 312
Schizosaccharomyces VR-----IWN PKLYPSDKAH RMPIITPAYP 307
Saccharomyces VR-----VWN PKIYAQDRSH RMPVITPAYP 308
Os03g19920 ---------- PWR--PHCCS FMPIVMPCSP 351
Sb01g037200 ---------- LCRG-PDGCS LMPIMLPCNP 322
Os07g48890 ---------- NCRR-PDG-S FMPILLPCTP 281
Sb02g043400 ---------- ---------- ---------- 296
At3g06560 ---------- PSTGAPP--G LMPIQLPCGS 289
GSVIVT00033174001 ---------- PTGGTRETRA LMPIQLPCSP 350
Conservation

	400		420	
At2g25850	CMNSSYNVSQ	STLRVMTEQF	QFGNTICQEI	355
At4g32850	CMNSSYNVSQ	STLRVMTEQF	QFGNNILQEI	353
PoplarXVIII	CMNSSYNVST	STLRVMTEQF	QSGNRILQEI	347
PoplarVI	CMNSSYNVST	STLRVMTEQF	QSGNRILQKI	334
GSVIVT00034292001	CMNSSYNVSI	STLRVMMEQF	QYGNKICEGI	351
Os06g36360	CMNSSYNVST	STLRVMMEQF	QFGNKICQEI	352
Sb10g022090	CMNSSYNVST	STLRVMVEQF	QFGNKICQEI	343
Os02g13400	CMNSSYNVST	STLRVIMEQF	QFGNKICQEI	356
Sb04g008100	CMNSSYNVSS	STLRVIMEQF	QFGNKICQEI	352
SelaginellaPAP1	CMNSSYNVSS	STLRVMVEEF	SRANGICEVI	342
SelaginellaPAP2	CMNSSYNVST	STLRIMTQEF	NRGNEVCEQL	332
GSVIVT00017746001	CMNSSYNVSS	STLRIMSEEF	KRGNEISEVM	348
PoplarXV	SMNSSYNVSS	STLRIMTEEF	QRGNEILQAM	332
At1g17980	CMNSSYNVSE	STLRIMKGEF	QRGNEICEAM	343
GSVIVT00030424001	SMNSSYNVSW	STLRIMEEEL	QRGNEIVKEM	347
Os06g21470	SMNSSYNVSI	STRHVMVQEF	TRASDICQAI	343
Sb01g012650	CMNSSYNVSV	STRYVMTQEF	TRAFEICQAI	343
GSVIVT00016654001	NMNSSYSVST	STLEAMMKQF	HTANKICNDI	343
mossPAP1	CQNSSFNVSN	STLRVMTEEF	KRGDSICDSL	339
mossPAP2	CQNSSFNVSN	STLRVMTEEF	KRGDGVCDSL	339
Os04g49870	CMNSGYNVSH	ATLRVITEQL	AVGDAVCQEI	345
Sb06g026810	CMNCTYNVSQ	ATQRIIKEQI	QAGHVACQEI	345
Dictyostelium	SMNSTYNVSK	STLQLMKSEF	VRGAEITRKI	383
mouse	QQNSTYNVSV	STRMVMVEEF	KQGLAITDEI	351
human	QQNSTYNVSV	STRMVMVEEF	KQGLAITDEI	351
mouse	QQNSTYNVSV	STRMVMIEEF	KQGLAITHEI	352
human	QQNSTYNVSI	STRMVMIEEF	KQGLAITHEI	351
mouse	QQNSTYNVST	STRTVMVEEF	KQGLAVTDEI	350
human	QQNSTYNVST	STRTVMVEEF	KQGLAVTDEI	350
Drosophila	QQNSTFNVSE	STKKVILTEF	NRGMNITDEI	359
Caenorhabditis	EQNSTHNVTR	STATVIKNEI	CEALEICRDI	347
Chlamydomonas	AMNSLYNVQR	STLEVMTEEF	AAAADVCTSF	342
Schizosaccharomyces	SMCATHNITL	STQTIILREM	VRAGEIADQI	337
Saccharomyces	SMCATHNITE	STKKVILQEF	VRGVQITNDI	338
Os03g19920	PEFCASSITR	STFNKIKEEL	QRGFALTKGD	381
Sb01g037200	PEFCSSSTTE	STFSKIKEEL	RRGYALTKDT	352
Os07g48890	PEFCTSNMTK	GTFKKIREEL	MRGYALTKEP	311
Sb02g043400	----------	----------	----------	296
At3g06560	HQYCNSTITR	STFYKIVAEF	LLGHNLTKDY	319
GSVIVT00033174001	YGYCHSNITK	STFYRITTEL	TLGHALTRDL	380

Conservation

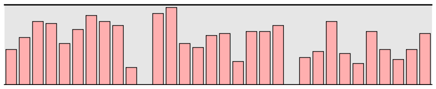

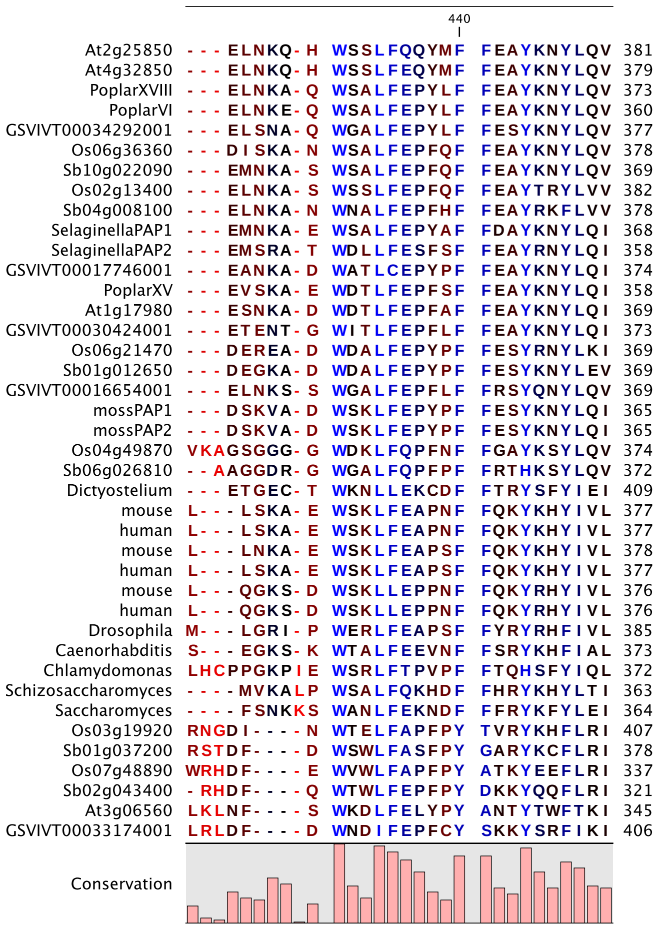
440
At2g25850 ---ELNKQ-H WSSLFQQYMF FEAYKNYLQV 381
At4g32850 ---ELNKQ-H WSSLFEQYMF FEAYKNYLQV 379
PoplarXVIII ---ELNKA-Q WSALFEPYLF FEAYKNYLQV 373
PoplarVI ---ELNKE-Q WSALFEPYLF FEAYKNYLQV 360
GSVIVT00034292001 ---ELSNA-Q WGALFEPYLF FESYKNYLQV 377
Os06g36360 ---DISKA-N WSALFEPFQF FEAYKNYLQV 378
Sb10g022090 ---EMNKA-S WSALFEPFQF FEAYKNYLQV 369
Os02g13400 ---ELNKA-S WSSLFEPFQF FEAYTRYLVV 382
Sb04g008100 ---ELNKA-N WNALFEPFHF FEAYRKFLVV 378
SelaginellaPAP1 ---EMNKA-E WSALFEPYAF FDAYKNYLQI 368
SelaginellaPAP2 ---EMSRA-T WDLLFESFSF FEAYRNYLQI 358
GSVIVT00017746001 ---EANKA-D WATLCEPYPF FEAYKNYLQI 374
PoplarXV ---EVSKA-E WDTLFEPFSF FEAYKNYLQI 358
At1g17980 ---ESNKA-D WDTLFEPFAF FEAYKNYLQI 369
GSVIVT00030424001 ---ETENT-G WITLFEPFLF FEAYKNYLQI 373
Os06g21470 ---DEREA-D WDALFEPYPF FESYRNYLKI 369
Sb01g012650 ---DEGKA-D WDALFEPYPF FESYKNYLEV 369
GSVIVT00016654001 ---ELNKS-S WGALFEPFLF FRSYQNYLQV 369
mossPAP1 ---DSKVA-D WSKLFEPYPF FESYKNYLQI 365
mossPAP2 ---DSKVA-D WSKLFEPYPF FESYKNYLQI 365
Os04g49870 VKAGSGGG-G WDKLFQPFNF FGAYKSYLQV 374
Sb06g026810 --AAGGDR-G WGALFQPFPF FRTHKSYLQV 372
Dictyostelium ---ETGEC-T WKNLLEKCDF FTRYSFYIEI 409
mouse L---LSKA-E WSKLFEAPNF FQKYKHYIVL 377
human L---LSKA-E WSKLFEAPNF FQKYKHYIVL 377
mouse L---LNKA-E WSKLFEAPSF FQKYKHYIVL 378
human L---LSKA-E WSKLFEAPSF FQKYKHYIVL 377
mouse L---QGKS-D WSKLLEPPNF FQKYRHYIVL 376
human L---QGKS-D WSKLLEPPNF FQKYRHYIVL 376
Drosophila M---LGRI-P WERLFEAPSF FYRYRHFIVL 385
Caenorhabditis S---EGKS-K WTALFEEVNF FSRYKHFIAL 373
Chlamydomonas LHCPPGKPIE WSRLFTPVPF FTQHSFYIQL 372
Schizosaccharomyces ----MVKALP WSALFQKHDF FHRYKHYLTI 363
Saccharomyces ----FSNKKS WANLFEKNDF FFRYKFYLEI 364
Os03g19920 RNGDI----N WTELFAPFPY TVRYKHFLRI 407
Sb01g037200 RSTDF----D WSWLFASFPY GARYKCFLRI 378
Os07g48890 WRHDF----E WVWLFAPFPY ATKYEEFLRI 337
Sb02g043400 -RHDF----Q WTWLFEPFPY DKKYQQFLRI 321
At3g06560 LKLNF----S WKDLFELYPY ANTYTWFTKI 345
GSVIVT00033174001 LRLDF----D WNDIFEPFCY SKKYSRFIKI 406
Conservation

	460		480	
At2g25850	D--VLAADAE	DLLAWKGWVE	SRFRQLTLKI	409
At4g32850	D--IVAADAE	DLLAWKGWVE	SRFRQLTLKI	407
PoplarXVIII	D--IVAAVAA	DLLVWKGWVE	SRLRQLTLKI	401
PoplarVI	D--IVAADAV	DLLAWKGWVE	SRLRQLTLKI	388
GSVIVT00034292001	D--IVAVDID	DLRAWKGWVE	SRLRQLTLMI	405
Os06g36360	D--IIAEDGE	DLRLWKGWVE	SRLRQLTLKI	406
Sb10g022090	D--IIAEDDE	DLRLWKGWVE	SRLRQLTLKI	397
Os02g13400	D--IVADDDD	DLRLWKGWIE	SRLRQLTLKI	410
Sb04g008100	D--IVAENDD	DLRLWKGWIE	SRLRQLTLKI	406
SelaginellaPAP1	D--VFAADND	DLRRWKGWVE	SRLRQLTLKI	396
SelaginellaPAP2	D--VVAIDDC	DHRCWKGWVE	SRLRQLTLKV	386
GSVIVT00017746001	E--IAAENAD	DLRKWKGWVE	SRLRQLTLKI	402
PoplarXV	D--ISAENED	DLRQWKGWVE	SRLRQLTLKV	386
At1g17980	D--ISAANVD	DLRKWKGWVE	SRLRQLTLKI	397
GSVIVT00030424001	D--ITAENDV	DLRNWKGWVE	SRLRLLTRKV	401
Os06g21470	E--ITARNED	DLRNWKGWVE	SRLRTLVLKI	397
Sb01g012650	N--ITARNED	ELRSWKGWVE	SRLRTLVLKI	397
GSVIVT00016654001	D--ITATDAD	DLRAWKGWVE	SRLRQLTLKV	397
mossPAP1	D--ISAGDEE	DLRIWKGWVE	SRLRQLILKV	393
mossPAP2	E--ITAGDEE	DLRIWKGWVE	SRLRQLILKV	393
Os04g49870	DVTVTGGEED	DLREWKGWVE	SRLRLLSARV	404
Sb06g026810	DATVAGGEEE	-LREWKGWVE	SRLRQLVAKV	401
Dictyostelium	DCY--SMNEE	DSRKWEGWIE	SKLRFLISNL	437
mouse	--LASAPTEK	QRLEWVGLVE	SKIRILVGSL	405
human	--LASAPTEK	QRLEWVGLVE	SKIRILVGSL	405
mouse	--LASAPTEK	QHLEWVGLVE	SKIRILVGSL	406
human	--LASASTEK	QHLEWVGLVE	SKIRILVGSL	405
mouse	--TASASTEE	NHLEWVGLVE	SKIRVLVGNL	404
human	--TASASTEE	NHLEWVGLVE	SKIRVLVGNL	404
Drosophila	--LVNSQTAD	DHLEWCGLVE	SKVRLLIGNL	413
Caenorhabditis	--IMAAPNEE	EELNYGGFLE	SRIRLLVQSL	401
Chlamydomonas	E--VSADSEG	DLVLWDGWVS	SRIRRLVRNL	400
Schizosaccharomyces	TAAAKTAEAQ	-LK-WAGLVE	SKLRHLVTRL	391
Saccharomyces	TAYTRGSDEQ	HLK-WSGLVE	SKVRLLVMKL	393
Os03g19920	--VLSAPVAE	ELRDWVGWVK	SRFRNLLLKL	435
Sb01g037200	--VLSAPLDE	ELRDWVGWVK	SRFRNLLLKL	406
Os07g48890	--ALCAPTSE	ELRDWAGWVK	SRFN-LILKL	364
Sb02g043400	--ALCAPTFA	ELRDWAGWVK	SRFRLLILKL	349
At3g06560	--HLSAANQE	DLSDWVGWVK	SRFRCLLIKI	373
GSVIVT00033174001	--YLSSSNQD	ELGDWVGWVK	SRFRFLLAKV	434

Conservation

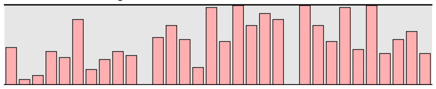

```
                                              500
                                               |
At2g25850          ERDTNGMLMC HPQPNEYV-- ---DTSKQFR 434
At4g32850          ERDTNGMLMC HPQPNEYV-- ---DTARQFL 432
PoplarXVIII        ERDTNGMLQC HPYPNEYI-- ---DASKQCP 426
PoplarVI           ERDTDGMLQC HPYPNEYI-- ---DPSKQCA 413
GSVIVT00034292001  ERDTFGKLQC HPYPHEYV-- ---DTSKQCS 430
Os06g36360         ERDTYGMLQC HPYPHEYA-- ---DPSRQCA 431
Sb10g022090        ERDTYGMLQC HPYPHEYA-- ---DPSRQCA 422
Os02g13400         ERDTKGMLQC HPNPCEYA-- ---DPSIQCA 435
Sb04g008100        DRDTKGILQC HPYPCEYS-- ---DPTIECA 431
SelaginellaPAP1    EKHTYGMLQC HPHPCDFV-- ---DESKEGK 421
SelaginellaPAP2    EKDTYGMLQC HPHPSDFV-- ---DMARDGY 411
GSVIVT00017746001  ERHTYNMLQC HPHPGDFS-- ---DKSRPF- 426
PoplarXV           KHQLLFLFGC ---------- --------L- 397
At1g17980          ERH-FKMLHC HPHPHDFQ-- ---DTSRPL- 420
GSVIVT00030424001  ---------- ---------- ---------- 401
Os06g21470         ERFTREMLLS HPNPRDFI-- ---DSSRPL- 421
Sb01g012650        ERYSHEMILA HPYPKDFS-- ---DKSRPL- 421
GSVIVT00016654001  ERCTIGKLLC VPCPREYV-- ---DTSRQCC 422
mossPAP1           EKDTFGALQC HPHPSAFH-- ---DTSKRVQ 418
mossPAP2           EKDTFGMLQC HPHPNAFH-- ---DTSKKVQ 418
Os04g49870         EADTSGMLLC HLHPQPYAAE PHNEPRRRRR 434
Sb06g026810        ERDTFGELLC HQNPRAYDAE PHG----LRC 427
Dictyostelium      E-STPKMKFA VPYPKGFTNN LHKANNPDQI 466
mouse              EKNEF-ITLA HVNPQSF-PA PK-------E 426
human              EKNEF-ITLA HVNPQSF-PA PK-------E 426
mouse              EKNEF-ITLA HVNPQSF-PA PK-------E 427
human              EKNEF-ITLA HVNPQSF-PA PK-------E 426
mouse              ERNEF-ITLA HVNPQSF-PG NK-------E 425
human              ERNEF-ITLA HVNPQSF-PG NK-------E 425
Drosophila         ERNPH-IALA HVNPKCF-EF KKGQSANNSQ 441
Caenorhabditis     ERNQD-IIIA HNDPNKHKPS PNAKFDVNPE 430
Chlamydomonas      E--------- ---------- ---------- 401
Schizosaccharomyces EL-VDAIALA HPFNKGFDKV YNCSSEEEAQ 420
Saccharomyces      EV-LAGIKIA HPFTKPFESS YCCPTEDDYE 422
Os03g19920         ESIG---VDC DPDPSEQADH SMIEPN---- 458
Sb01g037200        ESLG---VYC DPDSSEQVDH TITEPN---- 429
Os07g48890         ESIG---VEC DPDSTEEVDH TVFEPS---- 387
Sb02g043400        ERAG---IEC DPCPSEEVDH TDNDPN---- 372
At3g06560          EEVY---GIC DPNPTEYVET YTKQPN---- 396
GSVIVT00033174001  EEVQ---GLC DPNPTEFIDP DAGGPN---- 457
```

Conservation

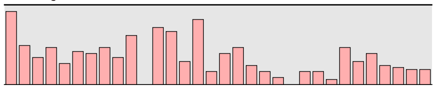

		520	540
At2g25850	H--------- ---------- ----------		435
At4g32850	H--------- ---------- ----------		433
PoplarXVIII	H--------- ---------- ----------		427
PoplarVI	H--------- ---------- ----------		414
GSVIVT00034292001	H--------- ---------- ----------		431
Os06g36360	H--------- ---------- ----------		432
Sb10g022090	H--------- ---------- ----------		423
Os02g13400	H--------- ---------- ----------		436
Sb04g008100	H--------- ---------- ----------		432
SelaginellaPAP1	H--------- ---------- ----------		422
SelaginellaPAP2	H--------- ---------- ----------		412
GSVIVT00017746001	H--------- ---------- ----------		427
PoplarXV	H--------- ---------- ----------		398
At1g17980	H--------- ---------- ----------		421
GSVIVT00030424001	---------- ---------- ----------		401
Os06g21470	H--------- ---------- ----------		422
Sb01g012650	H--------- ---------- ----------		422
GSVIVT00016654001	H--------- ---------- ----------		423
mossPAP1	V--------- ---------- ----------		419
mossPAP2	V--------- ---------- ----------		419
Os04g49870	T--------- ---------- ----------		435
Sb06g026810	A--------- ---------- ----------		428
Dictyostelium	C--------- ---------- ----------		467
mouse	SPDREE---- ---------- ----------		432
human	NPDKEE---- ---------- ----------		432
mouse	TADKEE---- ---------- ----------		433
human	NPDMEE---- ---------- ----------		432
mouse	HHKANN---- ---------- ----------		431
human	HHKDNN---- ---------- ----------		431
Drosophila	NNSGNEDDLK QSQGNQSAVT SAP-------		464
Caenorhabditis	NKR------- ---------- ----------		433
Chlamydomonas	---------- ---------- ----------		401
Schizosaccharomyces	QVASGVTLEV AYESTDHEKL ANDTVNEEKA		450
Saccharomyces	MIQDKYGSHK TETALNALKL VTDENKEEES		452
Os03g19920	---------- ---------- ----------		458
Sb01g037200	---------- ---------- ----------		429
Os07g48890	---------- ---------- ----------		387
Sb02g043400	---------- ---------- ----------		372
At3g06560	---------- ---------- ----------		396
GSVIVT00033174001	---------- ---------- ----------		457

Conservation

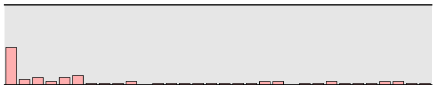

		560		
At2g25850	----------	----------	--CAFFMGLQ	443
At4g32850	----------	----------	--CAFFMGLQ	441
PoplarXVIII	----------	----------	--CAFFMGLQ	435
PoplarVI	----------	----------	--CAFFMGLQ	422
GSVIVT00034292001	----------	----------	--CAFFMGLQ	439
Os06g36360	----------	----------	--CAFFMGLS	440
Sb10g022090	----------	----------	--CAFFMGLS	431
Os02g13400	----------	----------	--CAFYMGLS	444
Sb04g008100	----------	----------	--CAFYMGLS	440
SelaginellaPAP1	----------	----------	--CAFFMGLQ	430
SelaginellaPAP2	----------	----------	--CAYFMGLQ	420
GSVIVT00017746001	----------	----------	--CCYFMGLQ	435
PoplarXV	----------	----------	--CSYFMGLQ	406
At1g17980	----------	----------	--CSYFMGLQ	429
GSVIVT00030424001	----------	----------	----ILLSLL	407
Os06g21470	----------	----------	--CFYFMGLW	430
Sb01g012650	----------	----------	--CFYFMGLW	430
GSVIVT00016654001	----------	----------	--CTYFMGLR	431
mossPAP1	----------	----------	--CSFFVALQ	427
mossPAP2	----------	----------	--CSFFVALQ	427
Os04g49870	----------	----------	--SSFFVGLS	443
Sb06g026810	----------	----------	--SSFFVGLS	436
Dictyostelium	----------	----------	--TSFFMGLS	475
mouse	----------	----------	FRTMWVIGLV	442
human	----------	----------	FRTMWVIGLV	442
mouse	----------	----------	FRTMWVIGLV	443
human	----------	----------	FRTMWVIGLG	442
mouse	----------	----------	YVSMWFLGII	441
human	----------	----------	YVSMWFLGII	441
Drosophila	----------	----------	FCSMWFIGLE	474
Caenorhabditis	----------	----------	-VTVWFIGLE	442
Chlamydomonas	----------	----------	----------	401
Schizosaccharomyces	DNTESKADGS	ENGEKQIFPV	YTTTCYIGLE	480
Saccharomyces	IKDAPKA---	----------	YLSTMYIGLD	469
Os03g19920	----------	----------	--VVFFWGLM	466
Sb01g037200	----------	----------	--VVFFWGLV	437
Os07g48890	----------	----------	--IVCHWGLI	395
Sb02g043400	----------	----------	--VVFYWGLI	380
At3g06560	----------	----------	--IVFYWGLQ	404
GSVIVT00033174001	----------	----------	--VVFFWGVQ	465

Conservation

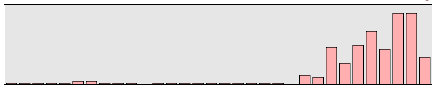

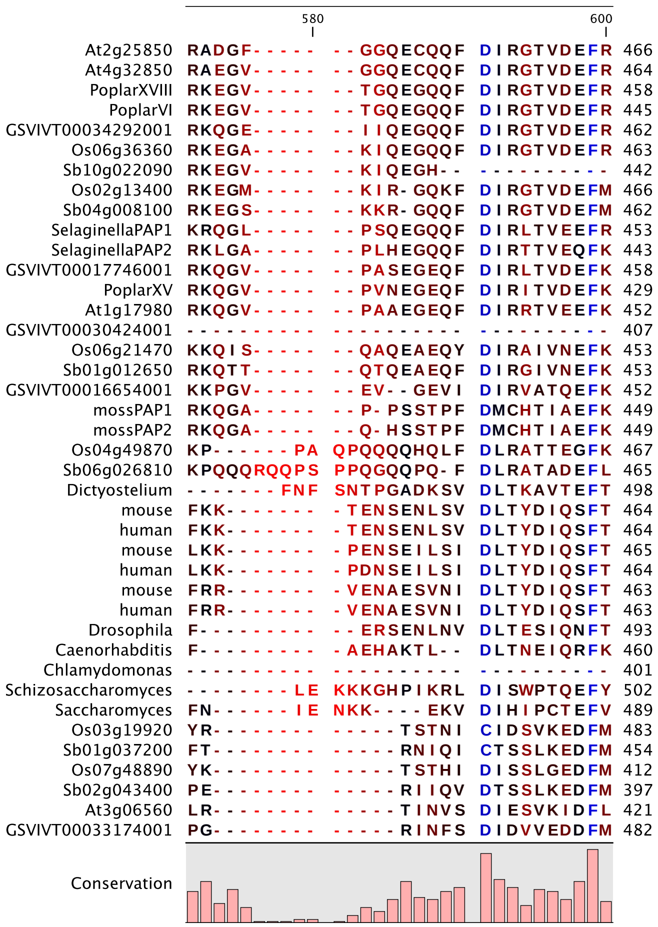
580
600
At2g25850 RADGF----- --GGQECQQF DIRGTVDEFR 466
At4g32850 RAEGV----- --GGQECQQF DIRGTVDEFR 464
PoplarXVIII RKEGV----- --TGQEGQQF DIRGTVDEFR 458
PoplarVI RKEGV----- --TGQEGQQF DIRGTVDEFR 445
GSVIVT00034292001 RKQGE----- --IIQEGQQF DIRGTVDEFR 462
Os06g36360 RKEGA----- --KIQEGQQF DIRGTVDEFR 463
Sb10g022090 RKEGV----- --KIQEGH-- ---------- 442
Os02g13400 RKEGM----- --KIR-GQKF DIRGTVDEFM 466
Sb04g008100 RKEGS----- --KKR-GQQF DIRGTVDEFM 462
SelaginellaPAP1 KRQGL----- --PSQEGQQF DIRLTVEEFR 453
SelaginellaPAP2 RKLGA----- --PLHEGQQF DIRTTVEQFK 443
GSVIVT00017746001 RKQGV----- --PASEGEQF DIRLTVDEFK 458
PoplarXV RKQGV----- --PVNEGEQF DIRITVDEFK 429
At1g17980 RKQGV----- --PAAEGEQF DIRRTVEEFK 452
GSVIVT00030424001 ---------- ---------- ---------- 407
Os06g21470 KKQIS----- --QAQEAEQY DIRAIVNEFK 453
Sb01g012650 RKQTT----- --QTQEAEQF DIRGIVNEFK 453
GSVIVT00016654001 KKPGV----- --EV--GEVI DIRVATQEFK 452
mossPAP1 RKQGA----- --P-PSSTPF DMCHTIAEFK 449
mossPAP2 RKQGA----- --Q-HSSTPF DMCHTIAEFK 449
Os04g49870 KP------PA QPQQQQHQLF DLRATTEGFK 467
Sb06g026810 KPQQQRQQPS PPQGQQPQ-F DLRATADEFL 465
Dictyostelium -------FNF SNTPGADKSV DLTKAVTEFT 498
mouse FKK------- -TENSENLSV DLTYDIQSFT 464
human FKK------- -TENSENLSV DLTYDIQSFT 464
mouse LKK------- -PENSEILSI DLTYDIQSFT 465
human LKK------- -PDNSEILSI DLTYDIQSFT 464
mouse FRR------- -VENAESVNI DLTYDIQSFT 463
human FRR------- -VENAESVNI DLTYDIQSFT 463
Drosophila F--------- --ERSENLNV DLTESIQNFT 493
Caenorhabditis F--------- -AEHAKTL-- DLTNEIQRFK 460
Chlamydomonas ---------- ---------- ---------- 401
Schizosaccharomyces --------LE KKKGHPIKRL DISWPTQEFY 502
Saccharomyces FN------IE NKK----EKV DIHIPCTEFV 489
Os03g19920 YR-------- -----TSTNI CIDSVKEDFM 483
Sb01g037200 FT-------- -----RNIQI CTSSLKEDFM 454
Os07g48890 YK-------- -----TSTHI DISSLGEDFM 412
Sb02g043400 PE-------- -----RIIQV DTSSLKEDFM 397
At3g06560 LR-------- -----TINVS DIESVKIDFL 421
GSVIVT00033174001 PG-------- -----RINFS DIDVVEDDFM 482
Conservation

620

At2g25850	QEV-NMYMFW	R--PG---MD	VHVSHVRRRQ	490
At4g32850	QEV-NMYMFW	K--PG---MD	VFVSHVRRRQ	488
PoplarXVIII	QEI-NMYMFW	K--PG---ME	IYVSHVRRRQ	482
PoplarVI	QDI-NMYLPW	K--PG---MD	IYVSHVRRRQ	469
GSVIVT00034292001	HSI-NMYMFW	K--PG---ME	IYVSHVRRKQ	486
Os06g36360	HDI-GMYGYW	R--PG---ME	LAVSHVRRKQ	487
Sb10g022090	----------	----------	----------	442
Os02g13400	HEI-GMYTQW	K--SG---MD	LAVTHVRKKE	490
Sb04g008100	REI-GMYSLW	M--PG---MD	LAVTHVQREQ	486
SelaginellaPAP1	QSV-TGYQLW	K--EG---MD	IAVSHVRRRQ	477
SelaginellaPAP2	LNV-AAYTSW	K--PG---ME	IYVSHVRRKQ	467
GSVIVT00017746001	HSV-GMYTLW	K--PG---ME	IHVIHVRRRN	482
PoplarXV	NSV-NMYTLW	K--PG---ME	IRVTHVKKRN	453
At1g17980	HTV-NAYTLW	I--PG---ME	ISVGHIKRRS	476
GSVIVT00030424001	----------	----------	----------	407
Os06g21470	SNI-HAYQHW	R--EG---ME	IEVSHVKRKD	477
Sb01g012650	NTI-CAYQQW	K--EG---MD	IEVSHVKRKE	477
GSVIVT00016654001	EEIVNMFSFW	T--PG---ME	IHVSHVLKNQ	477
mossPAP1	HSV-NQYLLW	K--PT---MK	IHVSHVRPKQ	473
mossPAP2	HSV-NQYLLW	K--PT---MK	IGVSHVRPKQ	473
Os04g49870	EEVY-MYDYW	R--PG---ME	VAVAHVRRKD	491
Sb06g026810	QDVY-TYRFW	R--PG---LE	LAVKHVRRKD	489
Dictyostelium	-GIIKDWLRT	Q--PNPDTMD	IKVQYIKKKQ	525
mouse	DTVYRQAINS	KMF--ELDMK	IAAMHVKRKQ	492
human	DTVYRQAINS	KMF--EVDMK	IAAMHVKRKQ	492
mouse	DTVYRQAINS	KMF--EMDMK	IAAMHLRRKE	493
human	DTVYRQAVNS	KMF--EMGMK	ITAMHLRRKE	492
mouse	DTVYRQANNI	NML--KDGMK	IEATHVKKKQ	491
human	DTVYRQANNI	NML--KEGMK	IEATHVKKKQ	491
Drosophila	EHVMMHGVNI	KML--KEGMT	IDARHVKRKQ	521
Caenorhabditis	TNVELQASNV	KGIGPNCQVQ	IDMFYVKRNS	490
Chlamydomonas	----------	----------	---DHVRVR-	407
Schizosaccharomyces	ELCKKW--DK	YD---DTLMN	VFIKNTKNTA	527
Saccharomyces	NLCRSFNEDY	GD---HKVFN	LALRFVKGYD	516
Os03g19920	KSVTNDIYGK	EKCT---HSD	ITMSIVWPTH	510
Sb01g037200	KSVCNNIYGK	EKCA---HSD	ITMAIVGPPQ	481
Os07g48890	KDVINDVYGK	VKGT---HSK	LTMSIVRSSQ	439
Sb02g043400	ESITNDVYGT	VKCT---HSD	VTISVVGLPQ	424
At3g06560	KNVNS---GS	FRGT---VGR	IQLTLVKASQ	445
GSVIVT00033174001	QNINN---GG	YQGP---PGK	MNLSVIPTSQ	506

Conservation

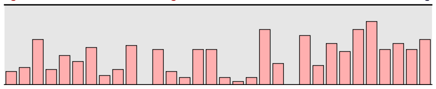

	640		660	
At2g25850	LPSFVFPNGY	KRSRQSR--H	QSQ-------	511
At4g32850	LPPFVFPNGY	RRPRQSR--H	QNL-------	509
PoplarXVIII	LPGFVFPDGY	KRSRSSR--H	INQ-------	503
PoplarVI	LPGFVFPDGY	KRSRPSR--H	VNQ-------	490
GSVIVT00034292001	IPSYVFPEGY	KRSRPQR--P	VNQ-------	507
Os06g36360	IPSYVFPEGY	KRPRPSR--H	INH-------	508
Sb10g022090	--SSIFVELW	M---------	----------	451
Os02g13400	IPLYVFEQGC	QKTRPPTPIC	AEQ-------	513
Sb04g008100	VPSYVFEQGY	KKPCPT--MH	ANQ-------	507
SelaginellaPAP1	IPAYVFPGGT	KPARPPKVTG	TGRLVGTNSL	507
SelaginellaPAP2	IPLFVYPGGV	KPARPA----	----------	483
GSVIVT00017746001	IPNFVFPGGV	RPSRPTKEAG	FRT-------	505
PoplarXV	IPNFVFPSGV	RPSRPSK---	----------	470
At1g17980	LPNFVFPGGV	RPSHTSKGTW	DSNRRSEHRN	506
GSVIVT00030424001	----------	----------	----------	407
Os06g21470	IPSFVFPGRI	RPSRPSRTVG	KEARAVSRSN	507
Sb01g012650	IPLFVFPGGV	RPSRSSRTAH	KNSRTVPTCD	507
GSVIVT00016654001	LPSYVFPDEY	RKRSQS----	----------	493
mossPAP1	IPTYVFPNGI	RPVRPPRPTI	HKPGTNPDNV	503
mossPAP2	IPTYVFPNGI	RPVRPPR---	----------	490
Os04g49870	LPSYVLRQLL	RSPG------	----------	505
Sb06g026810	LPPYVMHKI-	RGPN------	----------	502
Dictyostelium	LPAFVKDEGP	EEPVKT----	----------	541
mouse	LHQLLPSHVL	QKRKKHSTEG	V---------	513
human	LHQLLPNHVL	QKKKKHSTEG	V---------	513
mouse	LHQLLPNHVL	QKKETHLTES	V---------	514
human	LHQLLPHHVL	QDKKAHSTEG	R---------	513
mouse	LHHYLPAEIL	QKKKK-SLSD	VSRSSGGLQS	520
human	LHHYLPAEIL	QKKKKQSLSD	VNRSSGGLQS	521
Drosophila	LSLYLDSDFL	-KRERKSMES	HNNFNNTLLA	550
Caenorhabditis	LIQVISAADL	RRGRRW----	----------	506
Chlamydomonas	----------	----------	----------	407
Schizosaccharomyces	LPDEVFEPGE	ERPKATK---	----------	544
Saccharomyces	LPDEVFDENE	KRPSKKS---	----------	533
Os03g19920	LPKCVYAHS-	----------	----------	519
Sb01g037200	LPKSIFDLS-	----------	----------	490
Os07g48890	LPKSLYSHS-	----------	----------	448
Sb02g043400	LPKSMRSH--	----------	----------	432
At3g06560	LPKNGECGSN	NRSKKVTKTC	WRIREDKQCN	475
GSVIVT00033174001	LPGYAQLDTG	SRNR--TKAC	WRMFNYHQ-P	533

Conservation

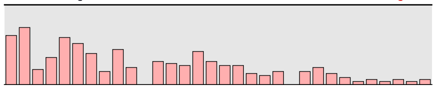

		680		
At2g25850	---------Q	CREPGDEGVG	SLSDSV-ERY	531
At4g32850	---------P	GGKSGEDGSV	SHSGSVVERH	530
PoplarXVIII	---------H	TSKTGEDVAR	SQSGS--ERP	522
PoplarVI	---------Q	TNRTSEDVAR	SQSGS--ERH	509
GSVIVT00034292001	---------Q	---QGDEACR	TGSS---EKH	522
Os06g36360	---------P	QQSNKNDVED	GTANRSPDGQ	529
Sb10g022090	----------	----------	----------	451
Os02g13400	---------Q	DRSGKNDSEV	CTTTVSLVGQ	534
Sb04g008100	---------Q	EQS---DGDV	-TLSPYLDSQ	524
SelaginellaPAP1	PTELDTPPLK	RKLDSETTPP	EGSRPLRRTN	537
SelaginellaPAP2	----------	----------	-------RQH	486
GSVIVT00017746001	----------	----------	----------	505
PoplarXV	----------	----------	----------	470
At1g17980	SSTSSAPAAT	TTTTEMSSES	KAGSNSPVDG	536
GSVIVT00030424001	----------	----------	----------	407
Os06g21470	ISA-------	----------	----------	510
Sb01g012650	VSA-------	----------	----------	510
GSVIVT00016654001	----------	----------	--SKSINQQH	501
mossPAP1	PTSQSPEAST	----------	----------	513
mossPAP2	----------	----------	----------	490
Os04g49870	----------	----------	------RHDQ	509
Sb06g026810	----------	----------	------IHE-	505
Dictyostelium	----------	----------	----------	541
mouse	KLTALNDSSL	DLSMDSD---	----------	530
human	KLTALNDSSL	DLSMDSD---	----------	530
mouse	RLTAVTDSSL	LLSIDSE---	----------	531
human	RLTDLNDSSF	DLSAGCE---	----------	530
mouse	KRSSLDSTCL	DSSRDTD---	----------	537
human	KRLSLDSSCL	DSSRDTD---	----------	538
Drosophila	NRKRL---ST	ELAQSQD---	----------	564
Caenorhabditis	KKVVPIATNT	SVSSSTP---	----------	523
Chlamydomonas	----------	----------	----------	407
Schizosaccharomyces	----------	----------	----------	544
Saccharomyces	----------	----------	----------	533
Os03g19920	---VYSQN--	----------	----------	524
Sb01g037200	---VYSEK--	----------	----------	495
Os07g48890	---VYTPY--	----------	----------	453
Sb02g043400	---VHWQY--	----------	----------	437
At3g06560	NVPVYSKH--	----------	----------	483
GSVIVT00033174001	RVPVFSQH--	----------	----------	541

Conservation

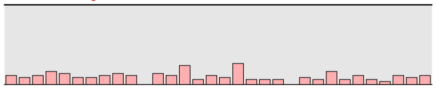

	700		720	
At2g25850	AKRKNDDE-I	MNSRPEKREK	RASCSLHTLD	560
At4g32850	AKRKNDSE-M	MDVRPEKPEK	RASLSPQSLD	559
PoplarXVIII	VKRKNDCE-M	EDLKP---EK	QACYSPVRPQ	548
PoplarVI	VKRKNDCE-M	ADLKPVKPEK	RASTSPQRLQ	538
GSVIVT00034292001	MKRKKDPE-E	VDVEQDKAAK	RLTISPQRQD	551
Os06g36360	PKRKHDTAGV	YDSEPGRSVK	RASISPSISP	559
Sb10g022090	----------	----------	----------	451
Os02g13400	LKRKYDSVGG	ADVDSFKSVR	RASVSP----	560
Sb04g008100	LKRKYDSDGD	GHVELHKSVK	WASVSPPGVG	554
SelaginellaPAP1	TGVSEAGSCQ	APAKPEKQEA	YADATPATLL	567
SelaginellaPAP2	SGSPSSDSKQ	GDSR--KRLT	SAD-------	507
GSVIVT00017746001	----------	----------	----------	505
PoplarXV	----------	----------	----------	470
At1g17980	KKRKWGDSET	LTDQPRNSKH	IAVSVPVENC	566
GSVIVT00030424001	----------	----------	----------	407
Os06g21470	----------	----------	----------	510
Sb01g012650	----------	----------	----------	510
GSVIVT00016654001	QNKRKINSEM	VDGKPSAS--	----------	519
mossPAP1	----------	----------	----------	513
mossPAP2	----------	----------	----------	490
Os04g49870	LKRKRADDDP	S-SSPAASDH	SASSSSSRDA	538
Sb06g026810	LKRKRDDDDS	SPSSPTLCSS	SSASSCDDDS	535
Dictyostelium	TKKRSSTGEP	SATRKKLKSE	NSDNKLNSPK	571
mouse	----------	----------	----------	530
human	----------	----------	----------	530
mouse	----------	----------	----------	531
human	----------	----------	----------	530
mouse	----------	----------	----------	537
human	----------	----------	----------	538
Drosophila	----------	----------	----------	564
Caenorhabditis	----------	----------	----------	523
Chlamydomonas	----------	----------	----------	407
Schizosaccharomyces	----------	----------	----------	544
Saccharomyces	----------	----------	----------	533
Os03g19920	----------	----------	----------	524
Sb01g037200	----------	----------	----------	495
Os07g48890	----------	----------	----------	453
Sb02g043400	----------	----------	----------	437
At3g06560	----------	----------	----------	483
GSVIVT00033174001	----------	----------	----------	541

Conservation

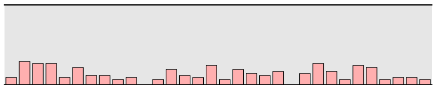

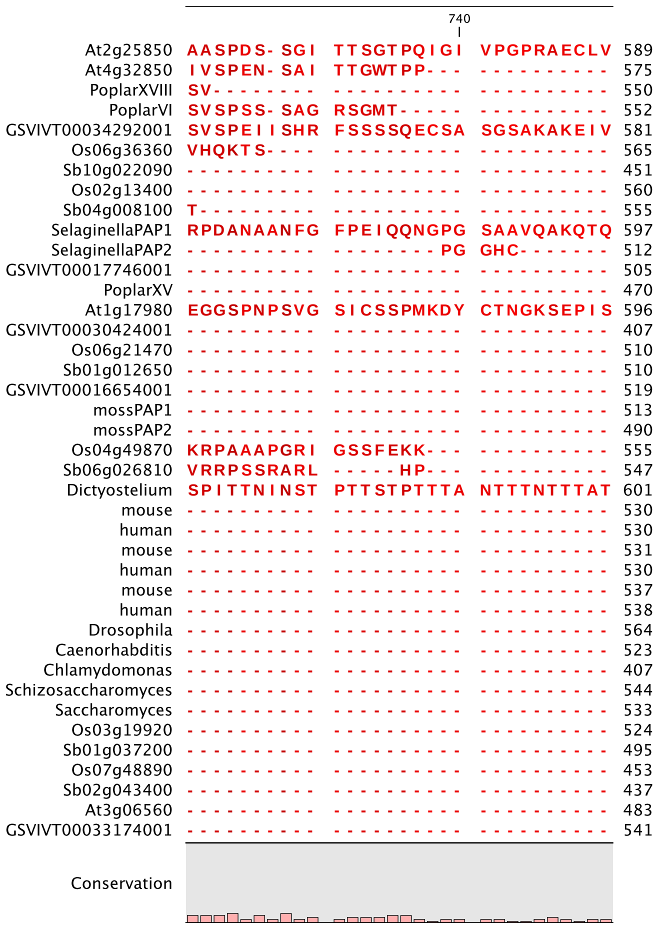
740
At2g25850 AASPDS-SGI TTSGTPQIGI VPGPRAECLV 589
At4g32850 IVSPEN-SAI TTGWTPP--- ---------- 575
PoplarXVIII SV-------- ---------- ---------- 550
PoplarVI SVSPSS-SAG RSGMT----- ---------- 552
GSVIVT00034292001 SVSPEIISHR FSSSSQECSA SGSAKAKEIV 581
Os06g36360 VHQKTS---- ---------- ---------- 565
Sb10g022090 ---------- ---------- ---------- 451
Os02g13400 ---------- ---------- ---------- 560
Sb04g008100 T--------- ---------- ---------- 555
SelaginellaPAP1 RPDANAANFG FPEIQQNGPG SAAVQAKQTQ 597
SelaginellaPAP2 ---------- --------PG GHC------- 512
GSVIVT00017746001 ---------- ---------- ---------- 505
PoplarXV ---------- ---------- ---------- 470
At1g17980 EGGSPNPSVG SICSSPMKDY CTNGKSEPIS 596
GSVIVT00030424001 ---------- ---------- ---------- 407
Os06g21470 ---------- ---------- ---------- 510
Sb01g012650 ---------- ---------- ---------- 510
GSVIVT00016654001 ---------- ---------- ---------- 519
mossPAP1 ---------- ---------- ---------- 513
mossPAP2 ---------- ---------- ---------- 490
Os04g49870 KRPAAAPGRI GSSFEKK--- ---------- 555
Sb06g026810 VRRPSSRARL -----HP--- ---------- 547
Dictyostelium SPITTNINST PTTSTPTTTA NTTTNTTTAT 601
mouse ---------- ---------- ---------- 530
human ---------- ---------- ---------- 530
mouse ---------- ---------- ---------- 531
human ---------- ---------- ---------- 530
mouse ---------- ---------- ---------- 537
human ---------- ---------- ---------- 538
Drosophila ---------- ---------- ---------- 564
Caenorhabditis ---------- ---------- ---------- 523
Chlamydomonas ---------- ---------- ---------- 407
Schizosaccharomyces ---------- ---------- ---------- 544
Saccharomyces ---------- ---------- ---------- 533
Os03g19920 ---------- ---------- ---------- 524
Sb01g037200 ---------- ---------- ---------- 495
Os07g48890 ---------- ---------- ---------- 453
Sb02g043400 ---------- ---------- ---------- 437
At3g06560 ---------- ---------- ---------- 483
GSVIVT00033174001 ---------- ---------- ---------- 541
Conservation

	760		780	
At2g25850	TGDLVCNVTS	LPNVEVEAEK	FISKITELRK	619
At4g32850	----VCNLRR	PPSEEIEADN	LNTECTELTD	601
PoplarXVIII	----------	----------	----------	550
PoplarVI	----------	----------	----------	552
GSVIVT00034292001	EGDRKCQVGM	GKLEDLVSTN	----------	601
Os06g36360	----------	----------	----------	565
Sb10g022090	----------	----------	----------	451
Os02g13400	----------	----------	----------	560
Sb04g008100	----------	----------	----------	555
SelaginellaPAP1	PATFVFQQTV	APLEELEAAG	TSS-------	620
SelaginellaPAP2	------SKKI	A---------	-SS-------	519
GSVIVT00017746001	----------	----------	----------	505
PoplarXV	----------	----------	----------	470
At1g17980	KDPPENVVAF	SKDPPESLPI	EKIATPQAHE	626
GSVIVT00030424001	----------	----------	----------	407
Os06g21470	----------	----------	----------	510
Sb01g012650	----------	----------	----------	510
GSVIVT00016654001	----------	----------	----------	519
mossPAP1	----------	----------	----------	513
mossPAP2	----------	----------	----------	490
Os04g49870	----------	----------	----------	555
Sb06g026810	----------	----------	----------	547
Dictyostelium	TTTTTTTVPI	TSTPTSNISS	PTMNSTELTT	631
mouse	----------	----------	----------	530
human	----------	----------	----------	530
mouse	----------	----------	----------	531
human	----------	----------	----------	530
mouse	----------	----------	----------	537
human	----------	----------	----------	538
Drosophila	----------	----------	----------	564
Caenorhabditis	----------	----------	----------	523
Chlamydomonas	----------	----------	----------	407
Schizosaccharomyces	----------	----------	----------	544
Saccharomyces	----------	----------	----------	533
Os03g19920	----------	----------	----------	524
Sb01g037200	----------	----------	----------	495
Os07g48890	----------	----------	----------	453
Sb02g043400	----------	----------	----------	437
At3g06560	----------	----------	----------	483
GSVIVT00033174001	----------	----------	----------	541

Conservation

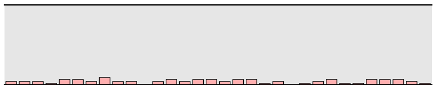

	800			
At2g25850	FSQYEHTSGS	EQILEVDSRA	LVQSYHDLAE	649
At4g32850	LARNECNSGS	EQVLEVDSMA	VVQECSDPAE	631
PoplarXVIII	----------	----------	----------	550
PoplarVI	----------	----------	----------	552
GSVIVT00034292001	----------	--VENIEMGA	IGRGMRWMKA	619
Os06g36360	----------	----------	----------	565
Sb10g022090	----------	----------	----------	451
Os02g13400	----------	----------	----------	560
Sb04g008100	----------	----------	----------	555
SelaginellaPAP1	----------	----------	----------	620
SelaginellaPAP2	----------	----------	----------	519
GSVIVT00017746001	----------	----------	----------	505
PoplarXV	----------	----------	----------	470
At1g17980	TEELEESFDF	GNQVIEQISH	KVAVLSATAT	656
GSVIVT00030424001	----------	----------	----------	407
Os06g21470	----------	----NVQERN	VPSMA-----	521
Sb01g012650	----------	----DDQVGN	LLGVASCSDA	526
GSVIVT00016654001	----------	----------	----------	519
mossPAP1	----------	----------	----------	513
mossPAP2	----------	----------	----------	490
Os04g49870	----------	----------	----------	555
Sb06g026810	----------	----------	----------	547
Dictyostelium	PTSTSTTTSN	DSITTPPTTT	TINSVQPPSA	661
mouse	----------	----------	----------	530
human	----------	----------	----------	530
mouse	----------	----------	----------	531
human	----------	----------	----------	530
mouse	----------	----------	----------	537
human	----------	----------	----------	538
Drosophila	----------	----------	----------	564
Caenorhabditis	----------	----------	----------	523
Chlamydomonas	----------	----------	----------	407
Schizosaccharomyces	----------	----------	----------	544
Saccharomyces	----------	----------	----------	533
Os03g19920	----------	----------	----------	524
Sb01g037200	----------	----------	----------	495
Os07g48890	----------	----------	----------	453
Sb02g043400	----------	----------	----------	437
At3g06560	----------	----------	----------	483
GSVIVT00033174001	----------	----------	----------	541

Conservation

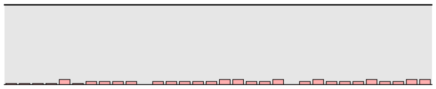

Sequence	820		840	
At2g25850	PVAKHVRPDL	SALLACEGGQ	NKEIGHDMGS	679
At4g32850	PLGKCVTPDS	VDVVACVSGQ	EENLDRNLRS	661
PoplarXVIII	----------	----------	----------	550
PoplarVI	----------	----------	----------	552
GSVIVT00034292001	DEKGNIEPDK	SDKPIPCTGN	AE--AGSVSN	647
Os06g36360	----------	----SPPSGN	IADASGASGG	581
Sb10g022090	----------	----------	------SLGM	455
Os02g13400	----------	----------	----------	560
Sb04g008100	----------	----------	----------	555
SelaginellaPAP1	----------	----------	----------	620
SelaginellaPAP2	----------	----------	----------	519
GSVIVT00017746001	----------	----------	----------	505
PoplarXV	----------	----------	----------	470
At1g17980	IPPFEATSNG	SPFPYEAVEE	LEVLPTRQPD	686
GSVIVT00030424001	----------	----------	----------	407
Os06g21470	QPMPYKSSEV	NKIPSDPHGG	YQSQERNNAV	551
Sb01g012650	QPVSCKGSYM	KQPEPDCAGG	FQLPGSTSVL	556
GSVIVT00016654001	----------	----------	----------	519
mossPAP1	----------	----------	----------	513
mossPAP2	----------	----------	----------	490
Os04g49870	----------	----------	----------	555
Sb06g026810	----------	----------	----------	547
Dictyostelium	QPTENGSSTS	NSPTSTSINN	TALPPNPTTN	691
mouse	----------	----------	---------N	531
human	----------	----------	---------N	531
mouse	----------	----------	---------N	532
human	----------	----------	---------N	531
mouse	----------	----------	---------S	538
human	----------	----------	---------N	539
Drosophila	----------	----------	---------P	565
Caenorhabditis	----------	----------	---------R	524
Chlamydomonas	----------	----------	----------	407
Schizosaccharomyces	----------	----------	----------	544
Saccharomyces	----------	----------	----------	533
Os03g19920	----------	----------	----------	524
Sb01g037200	----------	----------	----------	495
Os07g48890	----------	----------	----------	453
Sb02g043400	----------	----------	----------	437
At3g06560	----------	----------	----------	483
GSVIVT00033174001	----------	----------	----------	541

Conservation

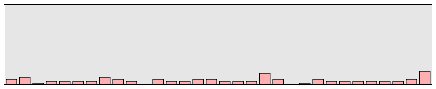

	851	860		
At2g25850	ESINDTDTQH	LPRRLNVNED	VDEVEREAKL	709
At4g32850	VSISGTDSPL	LPSR------	----------	675
PoplarXVIII	----------	----------	----------	550
PoplarVI	----------	----------	----------	552
GSVIVT00034292001	SSVVTSITSE	VSSSGDVGFE	SVGGSSDGNT	677
Os06g36360	SPVS------	----------	----------	585
Sb10g022090	TSIC------	----------	----------	459
Os02g13400	----------	----------	----------	560
Sb04g008100	----------	----------	----------	555
SelaginellaPAP1	----------	----------	----------	620
SelaginellaPAP2	----------	----------	----------	519
GSVIVT00017746001	----------	----------	----------	505
PoplarXV	----------	----------	----------	470
At1g17980	AAHRPSVQQR	KPIIKLSFTS	LGKTNGK---	713
GSVIVT00030424001	----------	----------	------I---	408
Os06g21470	VSSLPCEETG	HMFNGYANLH	TESVELEHLR	581
Sb01g012650	PPSLPNKVA-	--LNGSANFH	AESVEHEHPE	583
GSVIVT00016654001	----------	----------	----------	519
mossPAP1	----------	----------	----------	513
mossPAP2	----------	----------	----------	490
Os04g49870	----------	----------	----------	555
Sb06g026810	----------	----------	----------	547
Dictyostelium	SESTIETTIT	LPTTLESQTS	TLKDSNEIST	721
mouse	SMSVPSPTSA	--MK----TS	PLNSSGSS-Q	554
human	SMSVPSPTSA	--TK----TS	PLNSSGSS-Q	554
mouse	SMTAPSPTGT	--MK----TG	PL--TGNP-Q	553
human	SMSVPSSTST	--MK----TG	PL--ISSS-Q	552
mouse	GTPFNSPVSA	--NKPSNPDS	P--T-GEI-E	562
human	GTPFNSPASK	--S-----DS	P--SVGET-E	559
Drosophila	LPPGQQPSSG	--NRGRDSGA	KIQRLSDSLT	593
Caenorhabditis	SVVRTTSTSS	VPTTPTGLAA	PKTPLSASVS	554
Chlamydomonas	----------	----------	----------	407
Schizosaccharomyces	----------	----------	----------	544
Saccharomyces	----------	----------	----------	533
Os03g19920	----------	----------	----------	524
Sb01g037200	----------	----------	----------	495
Os07g48890	----------	----------	----------	453
Sb02g043400	----------	----------	----------	437
At3g06560	----------	----------	----------	483
GSVIVT00033174001	----------	----------	----------	541

Conservation

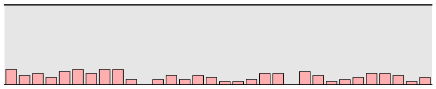

	880		900	
At2g25850	GEIAGGVLWN	GHCGRNLDHE	GFVTPANLDS	739
At4g32850	----------	-SCGQNRDYE	GFGFPAANSD	694
PoplarXVIII	----------	----------	----------	550
PoplarVI	----------	----------	----------	552
GSVIVT00034292001	GSVEGSNILG	ISQGDSCE--	-----ADSEL	700
Os06g36360	----------	----------	----------	585
Sb10g022090	----------	----------	----------	459
Os02g13400	----------	----------	----------	560
Sb04g008100	----------	----------	----------	555
SelaginellaPAP1	----------	----------	----------	620
SelaginellaPAP2	----------	----------	----------	519
GSVIVT00017746001	----------	----------	----------	505
PoplarXV	----------	----------	----------	470
At1g17980	----------	----------	----------	713
GSVIVT00030424001	----------	----------	----------	408
Os06g21470	SYKGSTSVPE	NHVVHDLVKP	PESMPPNSIH	611
Sb01g012650	HYQESKFATV	QNAVRNVVKQ	PNSLLPNSNN	613
GSVIVT00016654001	----------	----------	----------	519
mossPAP1	----------	----------	----------	513
mossPAP2	----------	----------	----------	490
Os04g49870	----------	----------	----------	555
Sb06g026810	----------	----------	----------	547
Dictyostelium	NGTAVATEPT	ITSPSVNINE	SSTSTSTTTT	751
mouse	GRNSPAPAVT	AASVTSIQ-A	SEVSVPQANS	583
human	GRNSPAPAVT	AASVTNIQ-A	TEVSVPQVNS	583
mouse	GRNSPALAVM	AASVTNIQ-F	PDVSLQHVNP	582
human	GRNSPALAVM	TASVANIQ-A	TEFSLQQVNT	581
mouse	RSSAEPVAVV	VEKLPSVPPA	QGLSIPVI--	590
human	RNSAEPAAVI	VEKPLSVPPA	QGLSIPVI--	587
Drosophila	EENSNASSDM	GAGTPTTPTT	AQLSAPSFKS	623
Caenorhabditis	ATNEPDSTTN	GTPLSRKRSM	DEESSTTVTS	584
Chlamydomonas	----------	----------	----------	407
Schizosaccharomyces	----------	----------	----------	544
Saccharomyces	----------	----------	----------	533
Os03g19920	----------	----------	----------	524
Sb01g037200	----------	----------	----------	495
Os07g48890	----------	----------	----------	453
Sb02g043400	----------	----------	----------	437
At3g06560	----------	----------	----------	483
GSVIVT00033174001	----------	----------	----------	541

Conservation

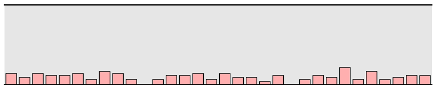

	920			
At2g25850	AVENRNLHSD	----------	----------	749
At4g32850	PMGKKNLYS-	----------	----------	703
PoplarXVIII	----------	----------	----------	550
PoplarVI	----------	----------	----------	552
GSVIVT00034292001	LLENGCVNAK	----------	----------	710
Os06g36360	-LANGNLEQA	NCLNSPLASE	KSLDSVTSGS	614
Sb10g022090	-ICSGSLEWS	----------	----------	468
Os02g13400	----------	----------	----------	560
Sb04g008100	----------	-----PPHGN	SVSNVVCDSP	570
SelaginellaPAP1	----------	----------	----------	620
SelaginellaPAP2	----------	----------	----------	519
GSVIVT00017746001	----------	----------	----------	505
PoplarXV	----------	----------	----------	470
At1g17980	----------	----------	----------	713
GSVIVT00030424001	----------	----------	----------	408
Os06g21470	VYPSPTNGLG	HLSDSSCKKP	ADIIVNKTTN	641
Sb01g012650	AWQ--LYGSD	SSLNNSQREC	AGSAANNLLN	641
GSVIVT00016654001	----------	----------	----------	519
mossPAP1	----------	----------	----------	513
mossPAP2	----------	----------	----------	490
Os04g49870	----------	----------	----------	555
Sb06g026810	----------	----------	----------	547
Dictyostelium	TTVTEQQIQT	APTTATPINK	TIVNTMEVNE	781
mouse	SESPGGPSSE	SIPQTATQPA	ISPPPKPTVS	613
human	SESSGGTSSE	SIPQTATQPA	ISPPPKPTVS	613
mouse	IESSGIALSE	SIPQIPSQPT	ISPPPKPTMT	612
human	NESSGVALNE	SIPHAVSQPA	ISPSPKAMVA	611
mouse	-----GAKVD	PTAKAVSSPA	VCTIPTVVGR	615
human	-----GAKVD	STVKTVSPPT	VCTIPTVVGR	612
Drosophila	S-GKNGSEID	VVEQEPTQP-	----------	641
Caenorhabditis	Q-----ISDE	SVPKKKTRDD	TLEENRVSMV	609
Chlamydomonas	----------	----------	----------	407
Schizosaccharomyces	----------	----------	----------	544
Saccharomyces	----------	----------	----------	533
Os03g19920	----------	----------	----------	524
Sb01g037200	----------	----------	----------	495
Os07g48890	----------	----------	----------	453
Sb02g043400	----------	----------	----------	437
At3g06560	----------	----------	----------	483
GSVIVT00033174001	----------	----------	----------	541

Conservation

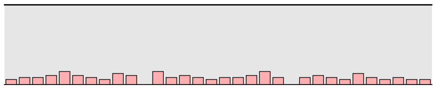

	940		960	
At2g25850	----------	----------	----------	749
At4g32850	----------	----------	----------	703
PoplarXVIII	----------	----------	----------	550
PoplarVI	----------	----------	----------	552
GSVIVT00034292001	----------	----------	----------	710
Os06g36360	KCVGVEAVCP	SDATKEHDNC	GSNMKNCTTT	644
Sb10g022090	----------	----------	----------	468
Os02g13400	-------ACE	EASIQ-LHDD	ANFGLTNCST	582
Sb04g008100	VKFVSSVVCS	RAQTSPSHDD	INLEQAQLTT	600
SelaginellaPAP1	----------	----------	----------	620
SelaginellaPAP2	----------	----------	----------	519
GSVIVT00017746001	----------	----------	----------	505
PoplarXV	----------	----------	----------	470
At1g17980	----------	----------	----------	713
GSVIVT00030424001	----------	----------	----------	408
Os06g21470	FSSAVLAVPD	ELDELDSH-Q	VKVNQKDLTA	670
Sb01g012650	LSPAILATPD	ELDELVSHHQ	VKVNQKDVNA	671
GSVIVT00016654001	----------	----------	----------	519
mossPAP1	----------	----------	----------	513
mossPAP2	----------	----------	----------	490
Os04g49870	----------	----------	----------	555
Sb06g026810	----------	----------	----------	547
Dictyostelium	LSFISSSSET	SQSKPPPKKP	TISIIRG---	808
mouse	RVVSSTRLVN	PSPRPSGNTA	T------KVP	637
human	RVVSSTRLVN	PPPRSSGNAA	TSGNAATKIP	643
mouse	RVVSSTHLVN	HPSRPSGNTA	T------NIP	636
human	RVVSSTCLIS	HPDLQETQQQ	T---------	632
mouse	NVIPRITTPH	NPVQGQPHLN	GISNITKN--	643
human	NVIPRITTPH	NPAQGQPHLN	GMSNITKT--	640
Drosophila	---------H	NNGNASSNTT	TTEVACS---	659
Caenorhabditis	VEVSNVVVEQ	RT--------	-------KVV	624
Chlamydomonas	----------	----------	----------	407
Schizosaccharomyces	----------	----------	----------	544
Saccharomyces	----------	----------	----------	533
Os03g19920	----------	----------	----------	524
Sb01g037200	----------	----------	----------	495
Os07g48890	----------	----------	----------	453
Sb02g043400	----------	----------	----------	437
At3g06560	----------	----------	----------	483
GSVIVT00033174001	----------	----------	----------	541

Conservation

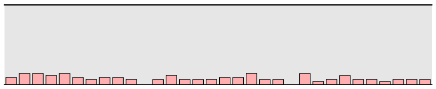

	980	
At2g25850	---------- ---------- ----------	749
At4g32850	---------- ---------- ----------	703
PoplarXVIII	---------- ---------- ----------	550
PoplarVI	---------- ---------- ----------	552
GSVIVT00034292001	---------- ---------- ----------	710
Os06g36360	TVAVSLKRVA EKVVSELVGS ESLGGNKSGE	674
Sb10g022090	---------- ---------- ----------	468
Os02g13400	SPHGSEGSTE SGNSCAAVGT IGLVDETSLP	612
Sb04g008100	SPYGSEDTSA SGTSFAAVGA VVLADESSKL	630
SelaginellaPAP1	---------- ---------- ----------	620
SelaginellaPAP2	---------- ---------- ----------	519
GSVIVT00017746001	---------- ---------- ----------	505
PoplarXV	---------- ---------- ----------	470
At1g17980	---------- ---------- ----------	713
GSVIVT00030424001	---------- ---------- ----------	408
Os06g21470	VDQGLSLEHK VGSNGGKAGT TGSPD-NNHL	699
Sb01g012650	-DRRPSLEIG SENNLEQVSS LRPQDSNNNL	700
GSVIVT00016654001	---------- ---------- ---GCMSGGM	526
mossPAP1	---------- ---------- ----------	513
mossPAP2	---------- ---------- ----------	490
Os04g49870	---------- ---------- ----------	555
Sb06g026810	---------- ---------- ----------	547
Dictyostelium	---------- ---------- ----------	808
mouse	NPIVGVKRTS SPNKEESPKK TKTEEDETSE	667
human	TPIVGVKRTS SPHKEESPKK TKTEEDETSE	673
mouse	NPILGV---- ---------- ----------	642
human	--YLIL---- ---------- ----------	636
mouse	---VTPKRSH SPPTDGTSKR LKDIEKFIRL	670
human	---VTPKRSH SPSIDGTPKR LKDVEKFIRL	667
Drosophila	---------- ---------- ----------	659
Caenorhabditis	QEIVDLQADN GLNT------ ----------	638
Chlamydomonas	---------- ---------- ----------	407
Schizosaccharomyces	---------- ---------- ----------	544
Saccharomyces	---------- ---------- ----------	533
Os03g19920	---------- ---------- ----------	524
Sb01g037200	---------- ---------- ----------	495
Os07g48890	---------- ---------- ----------	453
Sb02g043400	---------- ---------- ----------	437
At3g06560	---------- ---------- ----------	483
GSVIVT00033174001	---------- ---------- ----------	541

Conservation

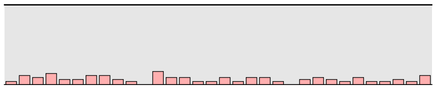

	1000		1020	
At2g25850	----------	----------	-GLFKSGLPE	758
At4g32850	----------	----------	----QSGMSE	709
PoplarXVIII	----------	----------	----------	550
PoplarVI	----------	----------	----------	552
GSVIVT00034292001	----------	----------	-EGFQDGLHE	719
Os06g36360	LLERAEDMGS	ALVENVHFGG	NGVVQTGLPE	704
Sb10g022090	----------	----------	----------	468
Os02g13400	GCLMPNVEND	PIRTILEQTP	SESVVEKDER	642
Sb04g008100	G-----LKSY	AINCLL----	----------	641
SelaginellaPAP1	----------	----------	----------	620
SelaginellaPAP2	----------	----------	----------	519
GSVIVT00017746001	----------	----------	----------	505
PoplarXV	----------	----------	----------	470
At1g17980	----------	----------	----------	713
GSVIVT00030424001	----------	----------	----------	408
Os06g21470	KRKAEEELEP	LELAAPLV-R	PPAPTSMTQR	728
Sb01g012650	KRKANQELEP	LELAAPSTGA	APQSTASAPR	730
GSVIVT00016654001	SNSSSQLKAD	MGSCKTGYKR	RRLTPEEGSC	556
mossPAP1	----------	----------	----------	513
mossPAP2	----------	----------	----------	490
Os04g49870	----------	----------	----------	555
Sb06g026810	----------	----------	----------	547
Dictyostelium	----------	----------	----------	808
mouse	DANCLALSGH	DKTETKEQVD	LETSAVQSET	697
human	DANCLALSGH	DKTEAKEQLD	TETSTTQSET	703
mouse	----------	----------	----------	642
human	----------	----------	----------	636
mouse	ES---AFKES	RAAEDRKRKP	MDSIGGESMP	697
human	ES---TFKDP	RTAEERKRKS	VDAIGGESMP	694
Drosophila	----------	----------	----------	659
Caenorhabditis	----------	----------	----------	638
Chlamydomonas	----------	----------	----------	407
Schizosaccharomyces	----------	----------	----------	544
Saccharomyces	----------	----------	----------	533
Os03g19920	----------	----------	----------	524
Sb01g037200	----------	----------	----------	495
Os07g48890	----------	----------	----------	453
Sb02g043400	----------	----------	----------	437
At3g06560	----------	----------	----------	483
GSVIVT00033174001	----------	----------	----------	541

Conservation

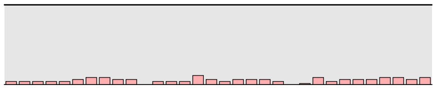

	1040			
At2g25850	ELQSNSLLSG	TGKLDDGARS	ESLQNEMMRH	788
At4g32850	DLQSNSLVSG	MEKSEDRAST	SRLS------	733
PoplarXVIII	----------	----------	----------	550
PoplarVI	----------	----------	----------	552
GSVIVT00034292001	ELEVLDLSS-	----------	--------LS	730
Os06g36360	ELEV------	----------	----------	708
Sb10g022090	----------	----------	----------	468
Os02g13400	KLEGIESLAS	SNCTEFMEVA	EVVAGTILTE	672
Sb04g008100	-LDG--NLVS	--C--LWELV	KIRMVQVLQP	664
SelaginellaPAP1	-IAASSAAAA	AAAEIRRPVI	RLCSASVPPS	649
SelaginellaPAP2	-SSSNQVVAA	EEVEVS----	----------	534
GSVIVT00017746001	----------	----------	----------	505
PoplarXV	----------	----------	----------	470
At1g17980	----------	----------	----------	713
GSVIVT00030424001	----------	----------	----------	408
Os06g21470	R---------	----------	---PLRLRLS	736
Sb01g012650	K---------	----------	---PLRLRLT	738
GSVIVT00016654001	EESKGLSVSE	SESESDGLAK	EKGSSLLQNA	586
mossPAP1	--RSVVTPPS	EEPELPIPGT	KRGAESLGVL	541
mossPAP2	----------	----------	----------	490
Os04g49870	----------	----------	----------	555
Sb06g026810	----------	----------	----------	547
Dictyostelium	----------	----------	----------	808
mouse	VPASASLLAS	QKTSSTDLSD	IPALPANPIP	727
human	IQTAASLLAS	QKTSSTDLSD	IPALPANPIP	733
mouse	----------	----------	----------	642
human	----------	----------	----------	636
mouse	IPTIDTARKK	RLPSKELPDS	SSPVPANNIR	727
human	IPTIDTSRKK	RLPSKELPDS	SSPVPANNIR	724
Drosophila	----------	----------	----------	659
Caenorhabditis	----------	----------	-----SNGLE	643
Chlamydomonas	----------	----------	----------	407
Schizosaccharomyces	-----KRSTA	DTAHSTEQLK	RQKVSTA---	566
Saccharomyces	-----KRKNL	DARHET--VK	RSKSDAASGD	556
Os03g19920	----------	------RQNP	RQFMMGNQLM	538
Sb01g037200	----------	------LP--	-QHMMGHQLM	506
Os07g48890	----------	------IP--	-QYMLGYQTP	464
Sb02g043400	----------	------MQ--	-RCMMAYEGT	448
At3g06560	----------	------LPG-	--YVVGYQKM	494
GSVIVT00033174001	----------	------LPL-	--YFVGYAA-	551

Conservation

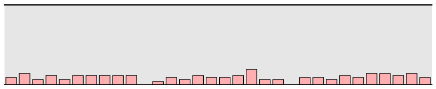

	1060		
At2g25850	VFLQPIIGLC	KS	800
At4g32850	--LKSTV---	--	738
PoplarXVIII	----------	--	550
PoplarVI	----------	--	552
GSVIVT00034292001	LYFFLFCFCF	LP	742
Os06g36360	----------	--	708
Sb10g022090	----------	--	468
Os02g13400	NGDIRLSGHE	VI	684
Sb04g008100	KYRNCCEG--	--	672
SelaginellaPAP1	TSAPPGNHFV	NR	661
SelaginellaPAP2	----------	--	534
GSVIVT00017746001	----------	--	505
PoplarXV	----------	--	470
At1g17980	----------	--	713
GSVIVT00030424001	----------	--	408
Os06g21470	TVVQPKPAEG	TS	748
Sb01g012650	TLGKPKPAEG	TS	750
GSVIVT00016654001	LLKEVEVSEN	FL	598
mossPAP1	GKRVRVELRS	ES	553
mossPAP2	----------	--	490
Os04g49870	----------	-T	556
Sb06g026810	----------	-T	548
Dictyostelium	----------	-N	809
mouse	VIKNSIKLRL	NR	739
human	VIKNSIKLRL	NR	745
mouse	----------	--	642
human	----------	--	636
mouse	VIKNSIRLTL	NR	739
human	VIKNSIRLTL	NR	736
Drosophila	----------	--	659
Caenorhabditis	ASEQKMEVPQ	SV	655
Chlamydomonas	----------	--	407
Schizosaccharomyces	----------	--	566
Saccharomyces	NINGTTAAVD	VN	568
Os03g19920	NQDCNA----	VR	546
Sb01g037200	KQRYNA----	VS	514
Os07g48890	TDYSGAA---	-G	472
Sb02g043400	DEGQSAGWLG	LG	460
At3g06560	VNREADGMEV	KC	506
GSVIVT00033174001	TDKDAKRLDA	WS	563

Conservation

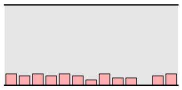

Supplement: Figure S1 — Global alignment of poly(A) polymerases. Sequences used for this alignment are given in File S1. Alignments were executed using the current version of the CLC Workbench suite of sequence analysis tools. In the display, deeper shades of red indicate more dissimilarity, and deeper shades of blue greater sequence similarity. A graphical depiction of sequence conservation is shown on the last line of the alignments. (4.72 MB PDF) [file pone.0008082.s001.pdf]
