# Supplementary material for: Characterization of Genes Encoding Poly(A) Polymerases in Plants: Evidence for Duplication and Functional Specialization
Source: PLoS One. 2009 Nov 26;4(11):e8082. doi: 10.1371/journal.pone.0008082 (PMC2778134; doi:10.1371/journal.pone.0008082)
Supplement: Figure S2 — Intron-exon organization of plant and mammalian poly(A) polymerase genes. The conserved “core” PAP sequences (File S2) were aligned and the alignment saved in the CLUSTAL format. Individual amino acid sequences were aligned to genome nucleotide databases and the output used to determine the positions of introns. These were added to the CLUSTAL alignment in the form of shading of the two amino acids that bound the intron positions. Green shading denotes introns in plant genes and blue shading the positions of introns in mammalian genes. (0.10 MB DOC) [file pone.0008082.s002.doc]

CLUSTAL 2.0.5 multiple sequence alignment

MusPAPa --------------------------------------------MPFPVTTQGS-----Q 11

humanPAPa --------------------------------------------MPFPVTTQGS-----Q 11

MusPAPb -------------------------------------------MMPFAVTTQGA-----Q 12

humanPAPb --------------------------------------------MPFPVTTQGP-----P 11

musPAPg -------------------------------------------MKEMSANTMLD-----S 12

humanPAPg -------------------------------------------MKEMSANTVLD-----S 12

ChlamydomonasPAP ----------------------------------------------MAVAD--------- 5

Os04g49870 ----------------------------------------------MASQSPQS------ 8

Sb06g026810 --------------------------------------------MAMASSQPKQ------ 10

Os06g36360 ----------------------------------------------MAACNAAA---AAA 11

Sb10g022090 ----------------------------------------------------------MA 2

Os02g13400 ----------------------------------------------MAGSFVAV---RGK 11

Sb04g008100 ----------------------------------------------MAGSVGKG---RAA 11

At2g25850 ----------------------------------------------MVSTQQRTDDDSSQ 14

At4g32850 ---------------------------------------------MMVGTQNLG---GSL 12

PoplarXVIII ----------------------------------------------MVGSQSSN------ 8

PoplarVI ----------------------------------------------MVGSQSPN------ 8

GSVIVT00034292001 ----------------------------------------------MVSSKGLG----DS 10

GSVIVT00016654001 ----------------------------------------------MENVKVRA------ 8

mossPAP1 --------------------------------------------------MEGS------ 4

mossPAP2 --------------------------------------------------MDGD------ 4

SelaginellaPAP1 -----------------------------------------------MAQSNGK------ 7

GSVIVT00017746001 -----------------------------------------MSNLGLNNRNNSG------ 13

At1g17980 ----------------------------------------------MASVQQNG------ 8

GSVIVT00030424001 ------------------------------------------MASVSASNQVNT------ 12

Os06g21470 ----------------------------------------------MAKSNNGN------ 8

Sb01g012650 ----------------------------------------------MSKAKTNN------ 8

Os03g19920 MSRSSRGGRQ-------SASSATRMASRARPGFPVAPPPPMGPPPPPPMPPVPVMYLRGV 53

Sb01g037200 MALDLAVG----------GSAARRAETQT-----LAPVLLMGPPPPPPIPPTTGVYLPG- 44

Sb02g043400 MAYMAAVAPVPWWPPPPELAPVGFPDASSPAGYPKPQTLPFLLAPTPPPPPPPPPLPAGY 60

GSVIVT00033174001 ---------------MAHAHRSQNSVALVHSQPLIHPQPPVVNAPVGFVPRPVGVINPSF 45

MusPAPa QTQPPQRHYG---ITSPISLAAPKETDCLLTQKLIETLKPFGVFEEEEELQRRILILGKL 68

humanPAPa QTQPPQKHYG---ITSPISLAAPKETDCVLTQKLIETLKPFGVFEEEEELQRRILILGKL 68

MusPAPb QPAPAPKQFG---ISSPISLAAPKDTDRELTQKLIETLQPFGVFEEEEELQRRILILQKL 69

humanPAPb QPAPPPNRYG---VSSPISLAVPKETDCLLTQRLIETLRPFGVFEEEEELQRRILVLEKL 68

musPAPg QRQ--QKHYG---ITSPISLACPKEIDHIYTQKLIDAMKPFGVFEDEEELNHRLVVLGKL 67

humanPAPg QRQ--QKHYG---ITSPISLASPKEIDHIYTQKLIDAMKPFGVFEDEEELNHRLVVLGKL 67

ChlamydomonasPAP -----PNDVY---LLRPLNNSLPSAEDKRHSAELEQFLRDAGLYEPDEDAYLRQEVLGLF 57

Os04g49870 ------RGVA-----EPISLVGPTPADLESTARLERLLREEGLYESAEETAAREEVLRGL 57

Sb06g026810 ------QMFG-----EPISLVGPTPADLEATAELEKVLREAGMYESPQESAVRAEVLRDL 59

Os06g36360 VAEQPQKQFG---ITKPISLAEPAEVDLQKTAELEKFLVEAGLYESPEESARREEVLGEL 68

Sb10g022090 SGSDPPKQYG---ITKPLSLLGPVEADLQRTAELEKFLVEAGLYESPDESARREEVLGKL 59

Os02g13400 PRSSPKRSGGGGGADPPLSLAMPTVADLHKTAELEKFLVEAGLYEGEEESAKREEVLREI 71

Sb04g008100 LRSSPKRYSG---TDPPLSLAGPTLADLQRTAELEKFLVEAGLYEGKEESAKREDVLCEI 68

At2g25850 PVKASLKSYG---ITEPLSIAGPSAADVKRNLELEKFLVDEGLYESKEETMRREEVVVRI 71

At4g32850 PPLNSPKSYG---ITKPLSLAGPSSADIKRNVELEKYLVDEGLYESKDDTMRREEVLGRI 69

PoplarXVIII --GTAAKRYG---ITKPISVAGPTEPDLHRNAELEKFLVDSGLNESKDETIKREEVLGRI 63

PoplarVI --GTAAKRYG---IMKPISVAGPTEPDLHRNAELEKQ--RRGYEERRCSGPYRS------ 55

GSVIVT00034292001 PPRQSVKQYG---VTKPISVAGPTEVDIQRSLELEKFLVDAGLYESKEEAIKRAEVLDRL 67

GSVIVT00016654001 ------KQFG---LTKPISYVKPTDFHIRRSFELEKVLWDGGVYQVEEEARKREEIIEKL 59

mossPAP1 ------RYLG---VTEPISTADPTEVDYVNTKQLEECVKGLALQGSREEEVRREEVLGRL 55

mossPAP2 ------RYLG---VTEPISTADPTEVDYANTKQLEEFVKGLALQGSREEEVRREEVLGLL 55

SelaginellaPAP1 ------KYLG---VTEPISTAGPTEADFARTRDLEKVLTEAGLYESPEEAVQREEVLGRL 58

SelaginellaPAP2 --------------MEPLSTAGPTPADLGRTRDLEKLLSNAGLNESREEAMKREGVLGRL 46

GSVIVT00017746001 ------QRLG---ITEPISLGGPNELDVTKTQELEKFLAAAGLYESQEEAVSREEVLGRL 64

PoplarXV ---------G---ITEPISLGGPTEYDVTKTRELEKFLQDAGLYESQEEAVSREEVLGRL 48

At1g17980 ------QRFG---VSEPISMGGPTEFDVIKTRELEKHLQDVGLYESKEEAVRREEVLGIL 59

GSVIVT00030424001 ------LCLG---VSEPISTAGPTEFDLIKTRELEKFLADSGLYETREEAIRREEVLGRV 63

Os06g21470 ------GYLG---VTEPISLSGPTEKDVVRTQEVEKCLADAGLYESQEEAVSREEVLGKL 59

Sb01g012650 ------GYLG---VTEPISLSGPTDKDLMQTTEVEKYLSDAGLYESQDEAVLREEVLGKL 59

Os03g19920 PPPPPWLPQHLIICGLDP--AAAERTDAFRSKSLLNFISRTGVLPSPEEELKRQVVVREL 111

Sb01g037200 PPPPGALLSRPIPMALPR--EVIVYMDECRSRSLLKFISDAGIVPSLEDERRRERVVREL 102

Os07g48890 --------MAPILLHLHP--AFLAQMDSRRTTSLLQDE---GGIPSPEADKKREQVIRKL 47

Sb02g043400 PLLP---PPAPIIIQLQPDPSFVAEVDQRRSSSLVQFLKDEGAVPSPEDEKKREKVIREL 117

GSVIVT00033174001 GPVPPFDPASLPQPGFVLNPAVLVRMEHRRSISLLQFMSNEGLIPSPEEELKRKNVIEKL 105

PoplarVIII -------------------------------------MVNEGLFPSPEEDEKRKIIVEKL 23

At3g06560 -----MKKGGGRNKGFPQ--------DDESSISLRQLMVNEGLIPSLEDEVKRRGVINQL 47

MusPAPa NNLVKEWIREISESKNLPQSVIENVGGKIFTFGSYRLGVHTKGADIDALCVAPRHVDR-S 127

humanPAPa NNLVKEWIREISESKNLPQSVIENVGGKIFTFGSYRLGVHTKGADIDALCVAPRHVDR-S 127

MusPAPb NNLVKEWIREISESRNLPQAVIENVGGKIFTFGSYRLGVHTKGADIDALCVAPRHVDR-N 128

humanPAPb NNLVKEWIREISESKSLPQSVIENVGGKIFTFGSYRLGVHTKGADIDALCVAPSHVDR-S 127

musPAPg NNLVKEWISDISESKNLPPSVVATVGGKIFTFGSYRLGVHTKGADIDALCVAPRHVER-S 126

humanPAPg NNLVKEWISDVSESKNLPPSVVATVGGKIFTFGSYRLGVHTKGADIDALCVAPRHVER-S 126

ChlamydomonasPAP YELTQTWVKGVCRKKNLN---VEDARAHVYTFGSYRLGVHGPGADMDTLVVGPRYVLRDS 114

Os04g49870 RGVVDRWVKRLTRQRGYPDGMADRATALVLPFGSYRLGVHGRGSDIDALVVGPSYVDCDR 117

Sb06g026810 QGIVDRWVKQLTLKHGYPDAMVDEATALLLPFGSYRLGVHGGGSDIDALVVGPSFVDRDQ 119

Os06g36360 DKIVKDWVKQLTSQRGYTDQMVEEANAVLFTFGSYRLGVHGPGADIDTLCVGPSYVNREE 128

Sb10g022090 DQIVKDWVKQLTSQRGYTDQMVEEANAVLFTFGSYRLGVHGPGADIDTLCVGPSYVNREE 119

Os02g13400 DQIVKEWVKKVTIQKGYSEQMVKEANAVLFTFGSYRLGVHGPGADIDALCIGPSYVKREE 131

Sb04g008100 GQIVKEWVKQLTSKKGYADQLVEQANAVLFTFGSYRLGVHGPEADIDTLCVGPSYVNREE 128

At2g25850 DQIVKHWVKQLTRQRGYTDQMVEDANAVIFTFGSYRLGVHGPMADIDTLCVGPSYVNREE 131

At4g32850 DQIVKHWVKQLTQQRGYTDQMVEDANAVIFTFGSYRLGVHGPGADIDTLCVGPSYVNREE 129

PoplarXVIII DQIVKDWVKQLTRQRGYTDQMVEEANAVIFTFGSYRLGVHGPGADIDTLCVGPSYVNREE 123

PoplarVI ----KDWVKRLTRQRGYTDQMVEEANAVIFTFGSYRLGVHGPGADIDTLCVGPSYVNRED 110

GSVIVT00034292001 GQIVKDWVKQLTRLRGYTDQMVEDANAVLFTFGSYRLGVHGPGTDIDTLCIGPSYVSREE 127

GSVIVT00016654001 RVVVKSWVKQVTRWKGYTDKMVENANALIVTFGSYRLGVHGPGSDIDTLCIGPSYVNREE 119

mossPAP1 DELVNVWVKSMSRKKGLNDEYVREARCKIFTFGSYRLGVHGPGADIDTLCVGPCYVTREE 115

mossPAP2 DELVNVWVKMVSRMKGLNDEYVREARCKIFTFGSYRLGVHGPGADIDTLCVGPCYVTREE 115

SelaginellaPAP1 DQLVKEWVKNICLRKGYSEQLTQEANAKIFTFGSYRLGVHGPGTDIDTLCVGPRHVSREE 118

SelaginellaPAP2 DQIVKSWVRQICVNKGFSNEVVQEANAKIFTFGSYRLGVHGPGSDIDTLCVGPSHATREE 106

GSVIVT00017746001 DQIVKIWVKAISRAKGLNEQLVQEANAKIFTFGSYRLGVHGPGADIDTLCVGPRHATREE 124

PoplarXV DQIVKNWVKVISRAKRLNEQLVQEANAKIFTFGSYRLGVHGPGADIDTLCVGPRHATREE 108

At1g17980 DQIVKTWIKTISRAKGLNDQLLHEANAKIFTFGSYRLGVHGPGADIDTLCVGPRHATREG 119

GSVIVT00030424001 DQIVKVWVKTVSRAKGFNEQLVHEANAKIFTFGSYRLGVHGPGADIDTLCVGPRHATRDE 123

Os06g21470 DQIVKAWIKKATRASGFGDQFVQEANAKIFTFGSYRLGVHGPGADIDTLCVGPRHATRTE 119

Sb01g012650 DQTVKAWIKKATRISGYGEQFVHEANAKIFTFGSYRLGVHGPGADIDTLCVGPRHATRNE 119

Os03g19920 DKIVMGWAKRVAYDQREQYW---NTTATVLTFGSYALGAYGPESDIDAVCVGPCIASLQH 168

Sb01g037200 GKIVMEWAKRVAYEQGKQHW---ITSATVLTFGSYALGAYGPESDIDVLCIGPYIATLQH 159

Os07g48890 NKIVMDWAKVVAYEQRVPPR---RATATVLTYGSYTLGAHGPESDIDALCVGPCIATLQY 104

Sb02g043400 KKIVMHWANAVAYEQSVPQG---LATATVLTYGSYTLGAHGPESDIDVLCVGPCIATLQY 174

GSVIVT00033174001 KEIVLTWVKRVAWQRQRPKQQIAVTSATILTYGSYGLGVHGPESDIDALCVGPFFASMAD 165

PoplarVIII KTIVVAWAKKVAWQRCLPKQQIAATSATILTYGSYGLGFHDPESDIDALCVGPFFATIAE 83

At3g06560 RKIVVRWVKNVAWQHRLPQNQIDATNATILPYGSYGLGVYGSESDIDALCIGPFFASIAD 107

MusPAPa DFFTS----FYDKLKLQ-EEVKDLRAVEEAFVPVIKLCFDGIEIDILFARLALQTIPEDL 182

humanPAPa DFFTS----FYDKLKLQ-EEVKDLRAVEEAFVPVIKLCFDGIEIDILFARLALQTIPEDL 182

MusPAPb DFFTS----FYDKLKLQ-EEVKDLRAVEEAFVPVIKLCFDGIEIDILFARLALQTIPEDL 183

humanPAPb DFFTS----FYAKLKLQ-EEVKDLRAVEEAFVPVIKLCFDGIEIDILFARLALQTIPEDL 182

musPAPg DFFQS----FFEKLKHQ-DGIRNLRAVEDAFVPVIKFEFDGIEIDLVFARLAIQTISDNL 181

humanPAPg DFFQS----FFEKLKHQ-DGIRNLRAVEDAFVPVIKFEFDGIEIDLVFARLAIQTISDNL 181

ChlamydomonasPAP DFFGSEKHCLEYMLSQT-PDITDIQPVPDAFVPMIGIKYKGVQIDILYASLAMQTLPEQL 173

Os04g49870 DFFGA----LATALAET-AAVAELQPVPGAHVPVIKMRFHGVQVDLVYAGVCLPVVPGDL 172

Sb06g026810 DFFGV----LAGALAEATEAVTDLQPVPGAHVPVMKLRFRGVQVDLVYASVNLPVVPRDL 175

Os06g36360 DFFIV----LHDILAQT-EEVTELQPVPDAHVPVMKFKFHGISIDLLYASVSLLVVPPDL 183

Sb10g022090 DFFIV----LHGILAQT-EDVTELQPVPDAHVPVMKFKFHGISIDLLYASVSLLVVPADL 174

Os02g13400 EFFVT----LYGALSEM-EEVTELQPVPDAHVPVMKFKFRGLPIDLLYASVSLPVIPPDF 186

Sb04g008100 DFFVT----LHGILAEK-EEVTELQPVPDAHVPVLKFKFRGISIDLLYASLSLSVIPADF 183

At2g25850 DFFIF----FRDILAEM-EEVTELQPVTDAHVPVMKFKFQGISIDLLYASISLLVIPQDL 186

At4g32850 DFFII----LHDILAEM-EEVTELHPVPDAHVPVMKFKFQGIPIDLLYASISLLVVPQDL 184

PoplarXVIII DFFIT----LHDKLAET-EEVTELQPVPDAHVPVMKFKFQGISIDLLYASISLLVVPQDL 178

PoplarVI -FFIV----LHDKLAEM-EEVTELQPVPDAHVPVMKFKFQGISIDLLYASISLLVPEQDL 165

GSVIVT00034292001 DFFFI----LHNILADM-EEVTELQPVPDAHVPVMKFKFDGISIDLLYASISLLVVPEDL 182

GSVIVT00016654001 DFFIR----LHNILIGM-EGVSELLPIPHAHVPVMKFKFEGVSIDLLYASVSHLVVPDDL 174

mossPAP1 DFFVE----LHDLLQKT-EGVTELHTVPDAHVPVMSFEFNGIPIDLLYARLPLWVIPEEL 170

mossPAP2 DFFVE----LHDLLQKT-DGVTELHTVPDAHVPVMSFEFNGIPIDLLYARLPLWVIPEEL 170

SelaginellaPAP1 DFFGV----FHGMLEAM-SEVTELHPVPDAHVPVMRFKFSGISIDLLYAPLAVWTIPEDL 173

SelaginellaPAP2 DFFVE----LHNILAET-ENVTELNPVPEAHVPVMKFKFDGISIDLLYARLSSWTIPEDL 161

GSVIVT00017746001 DFFGE----LHKMLSEM-PEVTELHPVPDAHVPVMRFKFSGVSIDLLYAKLSLWVIPEDL 179

PoplarXV DFFGE----LHRMLSEM-PEVTELHPVPDAHVPVMRFKFKGVSIDLLYAKLSLWVIPQDL 163

At1g17980 DFFGE----LQRMLSEM-PEVTELHPVPDAHVPLMGFKLNGVSIDLLYAQLPLWVIPEDL 174

GSVIVT00030424001 DFFGE----LHRMLAET-PEVQELHPVPDAHVPVMKFKFNGVSIDLLYARLSLWVIPEDL 178

Os06g21470 YFFQA----LYDMLVDM-PEVTELHPVPDAHVPVLKFKLNGVSIDLLYANLTHVVIPEDL 174

Sb01g012650 YFFRW----LHDMLAEM-PEVSELHPVPDAHVPVLGFKINGVSIDLLYANLAHAVIPEDL 174

Os03g19920 HFFIVLR----QMLEER-PEVSDLHSIENAKVPLMRFKFNGMLVDFPYVQLPVINAAEAI 223

Sb01g037200 HFFVVLR----QMLEGR-PEVSELQSIEGAKVPLMRFKFNGILVDFPYVQLPFINAAEAM 214

Os07g48890 HFFIVLR----QILEDR-PEVSELQTVESAKVPLMRFRFSGISVDFTYAQLPAI------ 153

Sb02g043400 HFFVVLR----QLLEGR-PEVSELQTIEKAKVPLMRFRFTGIAVDFTYAQLP-------- 221

GSVIVT00033174001 DFFIVLR----NMLESR-PEVSEIHCVKDAKVPLMRFKFDGISVDLPYAQLKLLYVPENL 220

**PoplarVIII** DFFIVLH----NILKSR-PEISEIHCVKDSKVPLMRFTFDGISVDLPYAQLKVLNVPENV 138

At3g06560 -FFISLR----DMLKSR-REVSELHCVKDAKVPLIRFKFDGILVDLPYAQLRVLSIPNNV 161

MusPAPa DLRDDSLLKNLDIRCIRSLNGCRVTDEILHLVPNIDNFRLTLRAIKLWAKRHNIYSNILG 242

humanPAPa DLRDDSLLKNLDIRCIRSLNGCRVTDEILHLVPNIDNFRLTLRAIKLWAKRHNIYSNILG 242

MusPAPb DLRDDSLLKNLDIRCIRSLNGCRVTDEILHLVPNIDSFRLTLRAIKLWAKCHNIYSNILG 243

humanPAPb DLRDDSLLKNLDIRCIRSLNGCRVTDEILHLVPNIDNFRLTLRAIKLWAKCHNIYSNILG 242

musPAPg DLRDDSRLRSLDIRCIRSLNGCRVTDEILHLVPNKETFRLTLRAVKLWAKRRGIYSNMLG 241

humanPAPg DLRDDSRLRSLDIRCIRSLNGCRVTDEILHLVPNKETFRLTLRAVKLWAKRRGIYSNMLG 241

ChlamydomonasPAP DLSNHAVLRGCDEPTVRALNGCRVTDTMLKLVPRQEVFRTALRAVKHWASLRGISSNVTG 233

Os04g49870 DLSGRSVLRGLDLATARSLNGVRVADEILRLVPDATAFRTTLRCVKHWAKARGVYSNVAG 232

Sb06g026810 DLSDRAVLRGLDHVTARSMNGVRVADEILRLVPDAAAFRTALRCVKLWAKARGVYSNVSG 235

Os06g36360 DISQGSVLYDVDEVTVRSLNGCRVADQILRLVPNVENFRTTLRCLKYWAKKRGVYSNVTG 243

Sb10g022090 DISQGSVLYDVDEATVRSLNGCRVADQIIRLVPNIENFRTTLRCLKYWAKRRGVYSNVTG 234

Os02g13400 DISQGSVLCDVDEATVRSLNGCRVADQILRLVPNAEIFRKTLRCLKYWAQRRGVYSNVTG 246

Sb04g008100 DISQGSVLCDVDEATVRSLNGCRVADQILRLVPNVENFRTTLRCLKYWAKRRGVYSNITG 243

At2g25850 DISNSSVLCDVDEQTVRSLNGCRVADQILKLVPNSEHFRTTLRCLKYWAKKRGVYSNVTG 246

At4g32850 DISSSSVLCEVDEPTVRSLNGCRVADQILKLVPNFEHFRTTLRCLKYWAKKRGVYSNVTG 244

PoplarXVIII DISNGSVLYEVDEQTVRSLNGCRVADQILKLVPNVEHFRTTLRCLKFWAKRRGVYSNVTG 238

PoplarVI DISNGSVLYEVDEQTVRSLNGCRVADQILKLVPNVEHFRATLRCLKFWAKRRGVYSNVTG 225

GSVIVT00034292001 DISDLSVLYNIDEPTVRSLNGCRVADQILKLVPNVEHFCTTLRCLKFWAKRRGVYSNVTG 242

GSVIVT00016654001 DISNESILYEADEPTVRSLSGCRVADQILRLVPNVEHFRTTLRCLKFWAKRRGVYSNVTG 234

mossPAP1 DILQDTILQNVDEQSVRSLNGCRVTDRILRLVPNMEHFRTTLRYVKLWAKRRGVYSNVIG 230

mossPAP2 DILQDSILQNVDEQSVRSLNGCRVTDRILRLVPNIEHFRTTLRYVKLWAKRRGVYSNVIG 230

SelaginellaPAP1 DISQESILRNLDEPSVRSLNGCRVTDQILRLVPNIEHFRTTLRCMKLWARKRGVYSNVTG 233

SelaginellaPAP2 DISDESIMQNLDEQSVLSLNGCRVTDQILRLVPNIQHFRTTLRCMKYWAKRRGVYSNVTG 221

GSVIVT00017746001 DVSQDSILQNADEQTVRSLNGCRVTDQILRLVPNIQNFRTTLRFMRFWAKRRGVYSNVAG 239

PoplarXV DVSQDSMLHNADEQTVRSLNGCRVTDQILRLVPNIQNFRTTLRCMRFWAKRRGVYSNVSG 223

At1g17980 DLSQDSILQNADEQTVRSLNGCRVTDQILRLVPNIQNFRTTLRCMRFWAKRRGVYSNVSG 234

GSVIVT00030424001 DISQETILQNVDEQTVRSLNGCRVTDQILRLVPNIQNFRTTLRCMRFWAKRRGVYSNVSG 238

Os06g21470 DLSHDSILHNVDEQTVRSLNGCRVTDKILRLVPNILTFRTTLRSLRFWAKRRGVYSNVIG 234

Sb01g012650 DLSQDSILNNVDEQTVRSLNGCRVTDQILRLVPNILSFRTTLRFIRYWGKRRGVYSNVMG 234

Os03g19920 HAFDPRLLAVVNEPSWRCLSGVRVNRQIMQLLPNIKKFQILLRCLKLWARKRGLHCHLLG 283

Sb01g037200 HAFDPHVLENVDGPSWRCLSGVRANRQIIQLVPNMK---------------------LLG 253

Os07g48890 ITSNPHLLQKLDSLSWRSLSGVRVNEQIVQLVPNAQKFQILLRCIKLWAKRRGIHCHLLG 213

Sb02g043400 ------------------------------VIDALKKFQALLRCIKLWARKRGLHCHYLG 251

GSVIVT00033174001 NVLNPYFLRNIDETSWKSLSGVRANECILQLVPNVENFQSILRCIKLWAKRRGVYGNLFG 280

PoplarVIII DILNLSLLTNIDETSWKSLSGVRANQRILLLVPNLMNFQSMLRCLKLWAKRRGVYGNLNG 198

At3g06560 DVLNPFFLRDIDETSWKILSGVRANKCILQLVPSLELFQSLLRCVKLWAKRRGVYGNLNG 221

MusPAPa FLGGVSWAMLVARTCQLYPNAIASTLVHKFFLVFSKWEWPNPVLLKQPEECN----LN-L 297

humanPAPa FLGGVSWAMLVARTCQLYPNAIASTLVHKFFLVFSKWEWPNPVLLKQPEECN----LN-L 297

MusPAPb FLGGVSWAMLVARTCQLYPNAIASTLVRKFFLVFSEWEWPNPVLLKEPEERN----LN-L 298

humanPAPb FLGGVSWAMLVARTCQLYPNAVASTLVRKFFLVFSEWEWPNPVLLKEPEERN----LN-L 297

musPAPg FLGGVSWAMLVARTCQLYPNAAASTLVHKFFLVFSKWEWPNPVLLKQPEESN----LN-L 296

humanPAPg FLGGVSWAMLVARTCQLYPNAAASTLVHKFFLVFSKWEWPNPVLLKQPEESN----LN-L 296

ChlamydomonasPAP YLGGVNLAIMVAKICQLYPRAEASTVLLKFFILLKAWPWPRAIHLRIPEEHS----LG-L 288

Os04g49870 FLG----RRLTG------QSSWRAMLLPRFFRVFARWKWPSPVMLRAIEHDDGELGLS-L 281

Sb06g026810 FLGGVAWAILVARVCQLYPNAAPSMLVSRFFKVLSQWKWPTPVMLCDIEHDD---ELG-L 291

Os06g36360 FLGGVNWALLVARVCQLYPNAVPSMLVSRFFRVFTQWQWPNPVMLCAIEEDE----LG-F 298

Sb10g022090 FLGGVNWALLVARVCQLYPNAVPSMLVSRFFRVFTQWQWPNPVMLCSIEEDE----VG-F 289

Os02g13400 LLGGVSWALLVARVCQLYPNAVPSMLVSRFFRVFTQWQWPNPVMLCAIENDDN---LG-F 302

Sb04g008100 FLGGVNWALLVARVCQLYPNAVPSMLVSRFFRVFTQWQWPNPVMLCAIENND----LG-F 298

At2g25850 FLGGVNWALLVARLCQFYPNAIPSMLVSRFFRVYTQWRWPNPVMLCAIEEDD----LS-F 301

At4g32850 FLGGVNWALLVARVCQLYPNAIPSMLVSRFFRVYTQWRWPNPVMLCAIEEDE----LG-F 299

PoplarXVIII FLGGVNWALLVARVCQLYPNAIPSMLVSRFFRVYTQWRWPNPVMLCSIEEDD----LG-F 293

PoplarVI FLGGVNWALLVARVCQLYPNAIPSMLVSRFFRVYTQWRWPNPVMLCSIEEDA----LG-F 280

GSVIVT00034292001 FLGGVNWALLVARVCQLYPNAVPSMLVSRFFRVYTQWRWPNPVMLCAIEEDE----LG-F 297

GSVIVT00016654001 FLGGVNLALLVARVCQLYPNANPSMLVSRFFRVYTQWHWPNPVMLCPIEDKE----LG-F 289

mossPAP1 FLGGVNWALLVARICQLYPNAVPSVLLSRFFRVYKQWRWPNPVMLCAIEE-GP---LG-L 285

mossPAP2 FLGGVNWALLVARICQLYPNAVPSVLLSRFFRVYKQWRWPNPVMLCAIEE-GS---LG-L 285

SelaginellaPAP1 FLGGVNWALLVARICQLYPNALPSMLVSRFFRVYTQWRWPNPVMLCEIEE-GS---LG-L 288

SelaginellaPAP2 FLGGINWALLVARICQLYPNAVPSTLVSRFFRVYTQWRWPNPVMLCPIEERSS---LGLL 278

GSVIVT00017746001 FLGGINWALLVARICQLYPNALPSMLVSRFFRVYTQWRWPNPVMLCAIEE-GT---LG-L 294

PoplarXV FLGGINWALLVARICQLFPNALPNMLVSRFFRVYTQWRWPNPVMLCAIEE-GS---LG-L 278

At1g17980 FLGGINWALLVARICQLYPNALPNILVSRFFRVFYQWNWPNAIFLCSPDE-GS---LG-L 289

GSVIVT00030424001 FLGGINWALLVARICQLYPNAVPSTLVSRFFRVYTQWRWPNPVMLCPIEE-KC---LG-L 293

Os06g21470 FLGGINWALLVARICQLYPNASPSMLISRFFKVYSKWKWPNPVMLCHIEE-GS---LG-L 289

Sb01g012650 FLGGINWAILVGRICQLYPNASPSMLISRFFRVYSKWKWPNPVMLCHIEE-GY---LG-L 289

Os03g19920 FFAGIHLAILAAFVCIMHPHATLSSLFNSFFDIFSHWHWPLPVSLLDQPTPW-------- 335

Sb01g037200 FFAGIHLAILAAYVCRRHPNASINTLLSLFFDIFAHWPWPLPVSLLDPPVLC-------- 305

Os07g48890 FFAGIHLAILAAYVCQRYPYGTINGLFTIFFDIFAHWNWQIPVSLHGQPTNC-------- 265

Sb02g043400 FFAGIHLAILAAYVCRKFPDASVNGLFAVFFQTFAHWPWQVPDSLR-------------- 297

GSVIVT00033174001 YFGGVHLAILAAFVCQKNPHANLNVLMSSFFKTFSGWPWPTPVALEDGRLP--------- 331

PoplarVIII FLGGVHLAVLAAFVCQNQPNASVIALISNFFSTYAMWPWPTPVMLQDGMSSN-------- 250

At3g06560 FLGGVHMAILAAFVCGYQPNATLSSLLANFFYTFAHWQWPTPVVLLEDTYP--------- 272

MusPAPa PVWDPRVNPSDRYHLMPIITPAYPQQNSTYNVSVSTRMVMVEEFKQGLAITDEILLSKA- 356

humanPAPa PVWDPRVNPSDRYHLMPIITPAYPQQNSTYNVSVSTRMVMVEEFKQGLAITDEILLSKA- 356

MusPAPb PVWDPRVNPSDRYHLMPIITPAYPQQNSTYNVSVSTRMVMIEEFKQGLAITHEILLNKA- 357

humanPAPb PVWDPRVNPSDRYHLMPIITPAYPQQNSTYNVSISTRMVMIEEFKQGLAITHEILLSKA- 356

musPAPg PVWDPRVNPSDRYHLMPIITPAYPQQNSTYNVSTSTRTVMVEEFKQGLAVTDEILQGKS- 355

humanPAPg PVWDPRVNPSDRYHLMPIITPAYPQQNSTYNVSTSTRTVMVEEFKQGLAVTDEILQGKS- 355

ChlamydomonasPAP PVWDPRPGTRDSLALMPVITPAYPAMNSLYNVQRSTLEVMTEEFAAAADVCTSFLHCPPG 348

Os04g49870 PVWDPRRNPRDKIHLMPIVTPAYPCMNSGYNVSHATLRVITEQLAVGDAVCQEIVKAGSG 341

Sb06g026810 PVWDGRRNPRDRTHLMPVITPAYPCMNCTYNVSQATQRIIKEQIQAGHVACQEIAAGGDR 351

Os06g36360 PVWDPRKYHRDRSHHMPIITPAYPCMNSSYNVSTSTLRVMMEQFQFGNKICQEIDISKAN 358

Sb10g022090 PVWDPRKNPRDRCHHMPIITPAYPCMNSSYNVSTSTLRVMVEQFQFGNKICQEIEMNKAS 349

Os02g13400 AVWDPRKNPRDRSHVMPIITPAYPCMNSSYNVSTSTLRVIMEQFQFGNKICQEIELNKAS 362

Sb04g008100 SIWDPRKNPRDRNHLMPIITPAYPCMNSSYNVSSSTLRVIMEQFQFGNKICQEIELNKAN 358

At2g25850 PVWDPRKNHRDRYHLMPIITPAYPCMNSSYNVSQSTLRVMTEQFQFGNTICQEIELNKQH 361

At4g32850 PVWDRRKNHRDRYHLMPIITPAYPCMNSSYNVSQSTLRVMTEQFQFGNNILQEIELNKQH 359

PoplarXVIII PVWDPRKNPRDRFHLMPIITPAYPCMNSSYNVSTSTLRVMTEQFQSGNRILQEIELNKAQ 353

PoplarVI PVWDPRKNPRDRFHHMPIITPAYPCMNSSYNVSTSTLRVMTEQFQSGNRILQKIELNKEQ 340

GSVIVT00034292001 SVWDPRKNPRDRTHHMPIITPAYPCMNSSYNVSISTLRVMMEQFQYGNKICEGIELSNAQ 357

GSVIVT00016654001 PVWDPRRNPLDRNHHMPIITPAYPNMNSSYSVSTSTLEAMMKQFHTANKICNDIELNKSS 349

mossPAP1 PVWDPRRNPRDRSHLMPIITPTYPCQNSSFNVSNSTLRVMTEEFKRGDSICDSLDSKVAD 345

mossPAP2 PIWDPRKNPRDRSHLMPIITPTYPCQNSSFNVSNSTLRVMTEEFKRGDGVCDSLDSKVAD 345

SelaginellaPAP1 SVWDPRKNPRDRTHQMPIITPAYPCMNSSYNVSSSTLRVMVEEFSRANGICEVIEMNKAE 348

SelaginellaPAP2 QVWDPRKNPRDKSHLMPIITPAYPCMNSSYNVSTSTLRIMTQEFNRGNEVCEQLEMSRAT 338

GSVIVT00017746001 QVWDPRKYPKDRFHLMPIITPAYPCMNSSYNVSSSTLRIMSEEFKRGNEISEVMEANKAD 354

PoplarXV SVWDPRRNPKDRYHLMPIITPAYPSMNSSYNVSSSTLRIMTEEFQRGNEILQAMEVSKAE 338

At1g17980 QVWDPRINPKDRLHIMPIITPAYPCMNSSYNVSESTLRIMKGEFQRGNEICEAMESNKAD 349

GSVIVT00030424001 PVWDPRRNIKDRNHLMPIITPAYPSMNSSYNVSWSTLRIMEEELQRGNEIVKEMETENTG 353

Os06g21470 LVWDPRRNFRDRGHHMPIITPAYPSMNSSYNVSISTRHVMVQEFTRASDICQAIDEREAD 349

Sb01g012650 PVWDPRRNYRDRGHQMPIITPAYPCMNSSYNVSVSTRYVMTQEFTRAFEICQAIDEGKAD 349

Os03g19920 -----R-PHCCS--FMPIVMPCSPPEFCASSITRSTFNKIKEELQRGFALTKGDRNGDIN 387

Sb01g037200 -----RGPDGCS--LMPIMLPCNPPEFCSSSTTESTFSKIKEELRRGYALTKDTRSTDFD 358

Os07g48890 -----RRPDGS---FMPILLPCTPPEFCTSNMTKGTFKKIREELMRGYALTKEPWRHDFE 317

Sb02g043400 --------------------------------------------------------HDFQ 301

GSVIVT00033174001 -----TGGTRETRALMPIQLPCSPYGYCHSNITKSTFYRITTELTLGHALTRDLLRLDFD 386

PoplarVIII -----VEDVIETRFYMPIRLPCSPYEYCHSNVTKSTFTKIRAEFLRGHSMTRDLLKLKLD 305

At3g06560 -------STGAPPGLMPIQLPCGSHQYCNSTITRSTFYKIVAEFLLGHNLTKDYLKLNFS 325

MusPAPa ----EWSKLFEAPNFFQKYKHYIVLLASAP--TEKQRLEWVGLVESKIRILVGSLEKN-E 409

humanPAPa ----EWSKLFEAPNFFQKYKHYIVLLASAP--TEKQRLEWVGLVESKIRILVGSLEKN-E 409

MusPAPb ----EWSKLFEAPSFFQKYKHYIVLLASAP--TEKQHLEWVGLVESKIRILVGSLEKN-E 410

humanPAPb ----EWSKLFEAPSFFQKYKHYIVLLASAS--TEKQHLEWVGLVESKIRILVGSLEKN-E 409

musPAPg ----DWSKLLEPPNFFQKYRHYIVLTASAS--TEENHLEWVGLVESKIRVLVGNLERN-E 408

humanPAPg ----DWSKLLEPPNFFQKYRHYIVLTASAS--TEENHLEWVGLVESKIRVLVGNLERN-E 408

ChlamydomonasPAP KPI-EWSRLFTPVPFFTQHSFYIQLEVSAD--SEGDLVLWDGWVSSRIRRLVRNLED--- 402

Os04g49870 GG--GWDKLFQPFNFFGAYKSYLQVDVTVTGGEEDDLREWKGWVESRLRLLSARVEADTS 399

Sb06g026810 ----GWGALFQPFPFFRTHKSYLQVDATVAGGEEE-LREWKGWVESRLRQLVAKVERDTF 406

Os06g36360 -----WSALFEPFQFFEAYKNYLQVDIIAE--DGEDLRLWKGWVESRLRQLTLKIERDTY 411

Sb10g022090 -----WSALFEPFQFFEAYKNYLQVDIIAE--DDEDLRLWKGWVESRLRQLTLKIERDTY 402

Os02g13400 -----WSSLFEPFQFFEAYTRYLVVDIVAD--DDDDLRLWKGWIESRLRQLTLKIERDTK 415

Sb04g008100 -----WNALFEPFHFFEAYRKFLVVDIVAE--NDDDLRLWKGWIESRLRQLTLKIDRDTK 411

At2g25850 -----WSSLFQQYMFFEAYKNYLQVDVLAA--DAEDLLAWKGWVESRFRQLTLKIERDTN 414

At4g32850 -----WSSLFEQYMFFEAYKNYLQVDIVAA--DAEDLLAWKGWVESRFRQLTLKIERDTN 412

PoplarXVIII -----WSALFEPYLFFEAYKNYLQVDIVAA--VAADLLVWKGWVESRLRQLTLKIERDTN 406

PoplarVI -----WSALFEPYLFFEAYKNYLQVDIVAA--DAVDLLAWKGWVESRLRQLTLKIERDTD 393

GSVIVT00034292001 -----WGALFEPYLFFESYKNYLQVDIVAV--DIDDLRAWKGWVESRLRQLTLMIERDTF 410

GSVIVT00016654001 -----WGALFEPFLFFRSYQNYLQVDITAT--DADDLRAWKGWVESRLRQLTLKVERCTI 402

mossPAP1 -----WSKLFEPYPFFESYKNYLQIDISAG--DEEDLRIWKGWVESRLRQLILKVEKDTF 398

mossPAP2 -----WSKLFEPYPFFESYKNYLQIEITAG--DEEDLRIWKGWVESRLRQLILKVEKDTF 398

SelaginellaPAP1 -----WSALFEPYAFFDAYKNYLQIDVFAA--DNDDLRRWKGWVESRLRQLTLKIEKHTY 401

SelaginellaPAP2 -----WDLLFESFSFFEAYRNYLQIDVVAI--DDCDHRCWKGWVESRLRQLTLKVEKDTY 391

GSVIVT00017746001 -----WATLCEPYPFFEAYKNYLQIEIAAE--NADDLRKWKGWVESRLRQLTLKIERHTY 407

PoplarXV -----WDTLFEPFSFFEAYKNYLQIDISAE--NEDDLRQWKGWVESRLRQLTLKVKHQLL 391

At1g17980 -----WDTLFEPFAFFEAYKNYLQIDISAA--NVDDLRKWKGWVESRLRQLTLKIERH-F 401

GSVIVT00030424001 -----WITLFEPFLFFEAYKNYLQIDITAE--NDVDLRNWKGWVESRLRLLTRKVILLSL 406

Os06g21470 -----WDALFEPYPFFESYRNYLKIEITAR--NEDDLRNWKGWVESRLRTLVLKIERFTR 402

Sb01g012650 -----WDALFEPYPFFESYKNYLEVNITAR--NEDELRSWKGWVESRLRTLVLKIERYSH 402

Os03g19920 WTE-----LFAPFPYTVRYKHFLRIVLSAP--VAEELRDWVGWVKSRFRNLLLKLESIG- 439

Sb01g037200 WSW-----LFASFPYGARYKCFLRIVLSAP--LDEELRDWVGWVKSRFRNLLLKLESLG- 410

Os07g48890 WVW-----LFAPFPYATKYEEFLRIALCAP--TSEELRDWAGWVKSRFN-LILKLESIG- 368

Sb02g043400 WTW-----LFEPFPYDKKYQQFLRIALCAP--TFAELRDWAGWVKSRFRLLILKLERAG- 353

GSVIVT00033174001 WND-----IFEPFCYSKKYSRFIKIYLSSS--NQDELGDWVGWVKSRFRFLLAKVEEVQ- 438

PoplarVIII SDVG---RIFEPFPYSTNYTRFVKIYLSAP--DQDELGDWVGWVKSHFRCLLLKLEAVQ- 359

At3g06560 WKD-----LFELYPYANTYTWFTKIHLSAA--NQEDLSDWVGWVKSRFRCLLIKIEEVY- 377

MusPAPa FITLAHVNPQSFPAPKESP-DREEFRTMWVIGLVFKKTE-------NSENLSVDLTYDIQ 461

humanPAPa FITLAHVNPQSFPAPKENP-DKEEFRTMWVIGLVFKKTE-------NSENLSVDLTYDIQ 461

MusPAPb FITLAHVNPQSFPAPKETA-DKEEFRTMWVIGLVLKKPE-------NSEILSIDLTYDIQ 462

humanPAPb FITLAHVNPQSFPAPKENP-DMEEFRTMWVIGLGLKKPD-------NSEILSIDLTYDIQ 461

musPAPg FITLAHVNPQSFPGNKEHH-KANNYVSMWFLGIIFRRVE-------NAESVNIDLTYDIQ 460

humanPAPg FITLAHVNPQSFPGNKEHH-KDNNYVSMWFLGIIFRRVE-------NAESVNIDLTYDIQ 460

ChlamydomonasPAP -----HVRVR-------------------------------------------------- 407

Os04g49870 GMLLCHLHPQPYAAEPHNEPRRRRRTSSFFVGLSKPPAQ-----PQQQQHQLFDLRATTE 454

Sb06g026810 GELLCHQNPRAYDAEPHG----LRCASSFFVGLSKPQQQRQQPSPPQGQQPQFDLRATAD 462

Os06g36360 GMLQCHPYPHEYADPSRQC-----AHCAFFMGLSRKEGA------KIQEGQQFDIRGTVD 460

Sb10g022090 GMLQCHPYPHEYADPSRQC-----AHCAFFMGLSRKEGV------KIQEG---------- 441

Os02g13400 GMLQCHPNPCEYADPSIQC-----AHCAFYMGLSRKEGM------KIRGQK-FDIRGTVD 463

Sb04g008100 GILQCHPYPCEYSDPTIEC-----AHCAFYMGLSRKEGS------KKRGQQ-FDIRGTVD 459

At2g25850 GMLMCHPQPNEYVDTSKQF-----RHCAFFMGLQRADGF------GGQECQQFDIRGTVD 463

At4g32850 GMLMCHPQPNEYVDTARQF-----LHCAFFMGLQRAEGV------GGQECQQFDIRGTVD 461

PoplarXVIII GMLQCHPYPNEYIDASKQC-----PHCAFFMGLQRKEGV------TGQEGQQFDIRGTVD 455

PoplarVI GMLQCHPYPNEYIDPSKQC-----AHCAFFMGLQRKEGV------TGQEGQQFDIRGTVD 442

GSVIVT00034292001 GKLQCHPYPHEYVDTSKQC-----SHCAFFMGLQRKQGE------IIQEGQQFDIRGTVD 459

GSVIVT00016654001 GKLLCVPCPREYVDTSRQC-----CHCTYFMGLRKKPG--------VEVGEVIDIRVATQ 449

mossPAP1 GALQCHPHPSAFHDTSKRV-----QVCSFFVALQRKQGA------P-PSSTPFDMCHTIA 446

mossPAP2 GMLQCHPHPNAFHDTSKKV-----QVCSFFVALQRKQGA------Q-HSSTPFDMCHTIA 446

SelaginellaPAP1 GMLQCHPHPCDFVDESKEG-----KHCAFFMGLQKRQGL------PSQEGQQFDIRLTVE 450

SelaginellaPAP2 GMLQCHPHPSDFVDMARDG-----YHCAYFMGLQRKLGA------PLHEGQQFDIRTTVE 440

GSVIVT00017746001 NMLQCHPHPGDFSDKSRP------FHCCYFMGLQRKQGV------PASEGEQFDIRLTVD 455

PoplarXV FLFGC-------------------LHCSYFMGLQRKQGV------PVNEGEQFDIRITVD 426

At1g17980 KMLHCHPHPHDFQDTSRP------LHCSYFMGLQRKQGV------PAAEGEQFDIRRTVE 449

GSVIVT00030424001 LI---------------------------------------------------------- 408

Os06g21470 EMLLSHPNPRDFIDSSRP------LHCFYFMGLWKKQIS------QAQEAEQYDIRAIVN 450

Sb01g012650 EMILAHPYPKDFSDKSRP------LHCFYFMGLWRKQTT------QTQEAEQFDIRGIVN 450

Os03g19920 --VDCDPDPSEQADHSMIE-----PNVVFFWGLMYRTST------------NICIDSVKE 480

Sb01g037200 --VYCDPDSSEQVDHTITE-----PNVVFFWGLVFTRNI------------QICTSSLKE 451

Os07g48890 --VECDPDSTEEVDHTVFE-----PSIVCHWGLIYKTST------------HIDISSLGE 409

Sb02g043400 --IECDPCPSEEVDHTDND-----PNVVFYWGLIPERII------------QVDTSSLKE 394

GSVIVT00033174001 --GLCDPNPTEFIDPDAGG-----PNVVFFWGVQPGRIN------------FSDIDVVED 479

PoplarVIII --GFCDPNPMEYVDMDASE-----PNVVFYWGLNRSRCN------------FVYIEPVEE 400

At3g06560 --GICDPNPTEYVETYTKQ-----PNIVFYWGLQLRTIN------------VSDIESVKI 418

MusPAPa SFTDTVYRQAINSKMFELDMKIAAMHVKRKQLHQLLPSHVLQKRKKHSTEGV-------- 513

humanPAPa SFTDTVYRQAINSKMFEVDMKIAAMHVKRKQLHQLLPNHVLQKKKKHSTEGV-------- 513

MusPAPb SFTDTVYRQAINSKMFEMDMKIAAMHLRRKELHQLLPNHVLQKKETHLTESV-------- 514

humanPAPb SFTDTVYRQAVNSKMFEMGMKITAMHLRRKELHQLLPHHVLQDKKAHSTEGR-------- 513

musPAPg SFTDTVYRQANNINMLKDGMKIEATHVKKKQLHHYLPAEILQKKKK-SLSDVSRSSGGLQ 519

humanPAPg SFTDTVYRQANNINMLKEGMKIEATHVKKKQLHHYLPAEILQKKKKQSLSDVNRSSGGLQ 520

Os04g49870 GFKEEVY----MYDYWRPGMEVAVAHVRRKDLPSYVLRQLLRSPGRHDQLKRKRAD---- 506

Sb06g026810 EFLQDVY----TYRFWRPGLELAVKHVRRKDLPPYVMHK-IRGPNIH-ELKRKRDD---- 512

Os06g36360 EFRHDIG----MYGYWRPGMELAVSHVRRKQIPSYVFPEGYKRPRP--SRHINHPQQSNK 514

Sb10g022090 --------------------------------HSSIFVELWMS----------------- 452

Os02g13400 EFMHEIG----MYTQWKSGMDLAVTHVRKKEIPLYVFEQGCQKTRPPTPICAEQQDRSGK 519

Sb04g008100 EFMREIG----MYSLWMPGMDLAVTHVQREQVPSYVFEQGYKKPCP--TMHANQQEQSDG 513

At2g25850 EFRQEVN----MYMFWRPGMDVHVSHVRRRQLPSFVFPNGYKRSRQ--SRHQSQQCREPG 517

At4g32850 EFRQEVN----MYMFWKPGMDVFVSHVRRRQLPPFVFPNGYRRPRQ--SRHQNLPGGKSG 515

PoplarXVIII EFRQEIN----MYMFWKPGMEIYVSHVRRRQLPGFVFPDGYKRSRS--SRHINQHTSKTG 509

PoplarVI EFRQDIN----MYLPWKPGMDIYVSHVRRRQLPGFVFPDGYKRSRP--SRHVNQQTNRTS 496

GSVIVT00034292001 EFRHSIN----MYMFWKPGMEIYVSHVRRKQIPSYVFPEGYKRSRP--QRPVNQQ---QG 510

GSVIVT00016654001 EFKEEIVN---MFSFWTPGMEIHVSHVLKNQLPSYVFPDEYRKRSQ-SSKSINQQHQNK- 504

mossPAP1 EFKHSVN----QYLLWKPTMKIHVSHVRPKQIPTYVFPNGIRPVRPPRPT---------- 492

mossPAP2 EFKHSVN----QYLLWKPTMKIGVSHVRPKQIPTYVFPNGIRPVRPPR------------ 490

SelaginellaPAP1 EFRQSVT----GYQLWKEGMDIAVSHVRRRQIPAYVFPGGTKPARPPKVTGTGR------ 500

SelaginellaPAP2 QFKLNVA----AYTSWKPGMEIYVSHVRRKQIPLFVYPGGVKPARP-------------- 482

GSVIVT00017746001 EFKHSVG----MYTLWKPGMEIHVIHVRRRNIPNFVFPGGVRPSRPTKVASERRRVLEP- 510

PoplarXV EFKNSVN----MYTLWKPGMEIRVTHVKKRNIPNFVFPSGVRPSRPSK------------ 470

At1g17980 EFKHTVN----AYTLWIPGMEISVGHIKRRSLPNFVFPGGVRPSHTSKGTWDSNRRSEH- 504

Os06g21470 EFKSNIH----AYQHWREGMEIEVSHVKRKDIPSFVFPGRIRPSRPSRTVGKEARAVSRS 506

Sb01g012650 EFKNTIC----AYQQWKEGMDIEVSHVKRKEIPLFVFPGGVRPSRSSRTAHKNSRTVPTC 506

Os03g19920 DFMKSVTNDI-YGKEKCTHSDITMSIVWPTHLPKCVYAHS-------------------- 519

Sb01g037200 DFMKSVCNNI-YGKEKCAHSDITMAIVGPPQLPKSIFDLS-------------------- 490

Os07g48890 DFMKDVINDV-YGKVKGTHSKLTMSIVRSSQLPKSLYSHS-------------------- 448

Sb02g043400 DFMESITNDV-YGTVKCTHSDVTISVVGLPQLPKSMRSH--------------------- 432

GSVIVT00033174001 DFMQNINNGG-YQGPPG---KMNLSVIPTSQLPGYAQLDTGSRNR--TKACWRMFNYHQP 533

PoplarVIII DFSRSIYCG--YYGIRG---KMELSIVQASELPKNARFDSGNGKK--MKACWKMLDYNQR 453

At3g06560 DFLKNVNSGS-FRGTVG---RIQLTLVKASQLPKNGECGSNNRSKKVTKTCWRIREDKQC 474
