# Supplementary material for: Characterization of Genes Encoding Poly(A) Polymerases in Plants: Evidence for Duplication and Functional Specialization
Source: PLoS One. 2009 Nov 26;4(11):e8082. doi: 10.1371/journal.pone.0008082 (PMC2778134; doi:10.1371/journal.pone.0008082)
Supplement: Table S1 — Primers and plasmids used in this study (0.10 MB DOC) [file pone.0008082.s003.doc]

Supplemental Table 1. Oligonucleotides and plasmids used in this research

| **primers** | **gene** | **sequence (5′->3′)** | **used for** |
| --- | --- | --- | --- |
| 7 5’ | Os07g48890 | **CCATGGAGGCATGATTTTGAGTGGG** | Cloning and sequencing rice PAP genes |
| 7 3’ | Os07g48890 | **CTAGCCAGCTGCGCCACTGTAGT** | Cloning and sequencing rice PAP genes |
| 6a5’1 | Os06g36360 | TATTCGGATCCGTGTGCACCGA | Cloning and sequencing rice PAP genes |
| 6a5’2 | Os06g36360 | CTGCAGAAGACAGCGGAGTTG | Cloning and sequencing rice PAP genes |
| 6a5’3 | Os06g36360 | CCATGGAATATCAATTGACCTTCT | Cloning and sequencing rice PAP genes |
| 6a5’4 | Os06g36360 | GGATCCACGCAAAATATCACCGTGAT | Cloning and sequencing rice PAP genes |
| 6a5’5 | Os06g36360 | GGTTTATCAAGGAAAGAAGG | Cloning and sequencing rice PAP genes |
| 6a3’1 | Os06g36360 | AGAACTTCTCCAACTCCGCTGTCTTCT | Cloning and sequencing rice PAP genes |
| 6a3’2 | Os06g36360 | GGATCCCACACAGGAAAACCAA | Cloning and sequencing rice PAP genes |
| 6a3’3 | Os06g36360 | AAGCTTTCACTTCCAACGAGCT | Cloning and sequencing rice PAP genes |
| 6a3’4 | Os06g36360 | AAGGACCAGTCTGCAAATATCTTATATTACAAGG | Cloning and sequencing rice PAP genes |
| 6a3’5 | Os06g36360 | CATTGCTTGACATGATGATC | Cloning and sequencing rice PAP genes |
| 6b5’1 | Os06g21470 | ATTCGGGTCGGATTGGAGCTGAA | Cloning and sequencing rice PAP genes |
| 6b5’2 | Os06g21470 | CTCGAGCTAGTGGTTTTGGCG | Cloning and sequencing rice PAP genes |
| 6b5’3 | Os06g21470 | CACGTGAGATGCTCCTCTCACAT | Cloning and sequencing rice PAP genes |
| 6b5’4 | Os06g21470 | AGCATGGCCCAGCCTATGCC | Cloning and sequencing rice PAP genes |
| 6b5’5 | Os06g21470 | GATAAAATTCTACGATTGGTTCC | Cloning and sequencing rice PAP genes |
| 6b3’1 | Os06g21470 | CGCCAAAACCACCACTAGCTCGAGTT | Cloning and sequencing rice PAP genes |
| 6b3’2 | Os06g21470 | CACGTGTAAATCGTTCAATCTTTAAT | Cloning and sequencing rice PAP genes |
| 6b3’3 | Os06g21470 | GGCAAAACACGATAATTTTATTAGAACTTGG | Cloning and sequencing rice PAP genes |
| 6b3’4 | Os06g21470 | CAGATGATTGTTATCAGGTG | Cloning and sequencing rice PAP genes |
| 6b3’5 | Os06g21470 | CTGCAGTTAAATCTTTCTGATTTACCT | Cloning and sequencing rice PAP genes |
| 35’1 | Os03g19920 | ATGGGTTGGGCGAAGAGGGTG | Cloning and sequencing rice PAP genes |
| 35’2 | Os03g19920 | CTGCAGAGGCAATACATGCATTTG | Cloning and sequencing rice PAP genes |
| 35’3 | Os03g19920 | CTTTCTGCTCCAGTGGCTGAAG | Cloning and sequencing rice PAP genes |
| 35’4 | Os03g19920 | TCTGATATAGACGCAGTCTG | Cloning and sequencing rice PAP genes |
| 35’5 | Os03g19920 | TCTGGAGTTCGTGTTAATAG | Cloning and sequencing rice PAP genes |
| 33’1 | Os03g19920 | GCTAGCAAGCGAGGGTCAAATGCAT | Cloning and sequencing rice PAP genes |
| 33’2 | Os03g19920 | CTGCAGCATTGATAACTGGCAA | Cloning and sequencing rice PAP genes |
| 33’3 | Os03g19920 | CTTTCTCTGGATGACTTGCCACATA | Cloning and sequencing rice PAP genes |
| 33’4 | Os03g19920 | GCTCGATTGAGCTTACTTTGC | Cloning and sequencing rice PAP genes |
| 33’5 | Os03g19920 | agagctgagagtagcatgtg | Cloning and sequencing rice PAP genes |
| 33’6 | Os03g19920 | ACTTCTCCTTTCCATAGATG | Cloning and sequencing rice PAP genes |
| 25’1 | Os02g13400 | ATGGCGGGATCTTTCGTCGCG | Cloning and sequencing rice PAP genes |
| 25’2 | Os02g13400 | GTGCCAAGTATGCTGGTTTCAAG | Cloning and sequencing rice PAP genes |
| 25’3 | Os02g13400 | GGGCCCTATTGGTTGCTCGTGTCT | Cloning and sequencing rice PAP genes |
| 25’4 | Os02g13400 | GATAGATCACATGTTATGCC | Cloning and sequencing rice PAP genes |
| 25’5 | Os02g13400 | GTGGTTGACATTGTTGCAGATG | Cloning and sequencing rice PAP genes |
| 25’6 | Os02g13400 | GTGATGTTGATGAAGCAACT | Cloning and sequencing rice PAP genes |
| 25’7 | Os02g13400 | TGGAGCAGACATTGATGCAC | Cloning and sequencing rice PAP genes |
| 25’8 | Os02g13400 | GCAGTCCTTTTCACTTTTGG | Cloning and sequencing rice PAP genes |
| 23’1 | Os02g13400 | CCTCTCGTTTCACATATGAGGGTCCAATA | Cloning and sequencing rice PAP genes |
| 23’2 | Os02g13400 | AGCATTGGGATAAAGTTGGCAG | Cloning and sequencing rice PAP genes |
| 23’3 | Os02g13400 | AAAACTACCAAATATACTTACCTAGACCTAACTAG | Cloning and sequencing rice PAP genes |
| 23’4 | Os02g13400 | AAGCTTTCAATTCCTTCTAGTTTTCTTTCATCC | Cloning and sequencing rice PAP genes |
| 23’5 | Os02g13400 | CGCTTGAGCTGACCCACCAG | Cloning and sequencing rice PAP genes |
| 23’6 | Os02g13400 | GGCGCCATCTGTTCAAGTCTC | Cloning and sequencing rice PAP genes |
| 23’7 | Os02g13400 | CACTTCATGTCCACTTAAGCG | Cloning and sequencing rice PAP genes |
| 23’8 | Os02g13400 | CAGCATTGGGATAAAGTTGG | Cloning and sequencing rice PAP genes |
| WiscDsLox4-13-4 16L14 5’ | At1g17980 | CTCATGTTCCATTGATGGGATTCAAACTTA | Genotyping Arabidopsis mutants |
| WiscDsLox4-13-4 16L14 3’ | At1g17980 | ATGGCGTTCGGCCAATTCCATTCATAGAAG | Genotyping Arabidopsis mutants |
| WiscDsLox441G5 5’ | At1g17980 | CGCTTACACATTGTGGATTCCAGGGATGGA | Genotyping Arabidopsis mutants |
| WiscDsLox441G5 3’ | At1g17980 | TTACTTGCCATTGGTTTTGCCTAGAGACGT | Genotyping Arabidopsis mutants |
| SALK_083263 5’ | At2g25850 | AGTGGTATCACTACTAGTGGGACTCCTC | Genotyping Arabidopsis mutants |
| SALK_083263 3’ | At2g25850 | TCATGATTTGCATAAACCAATAATGGGTT | Genotyping Arabidopsis mutants |
| SALK_129612 5’ | At3g06560 | GGTAATTTGAATGGATTTCTAGGGGGAGT | Genotyping Arabidopsis mutants |
| SALK_129612 3’ | At3g06560 | CTAACATTTAACCTCCATACCATCAGCTTC | Genotyping Arabidopsis mutants |
| SALK_133557 5’ | At3g06560 | GGTAATTTGAATGGATTTCTAGGGGGAGT | Genotyping Arabidopsis mutants |
| SALK_133557 3’ | At3g06560 | CACTGAGATCTTCCTGATTAGCAGCCGAG | Genotyping Arabidopsis mutants |
| SAL_ 063790 5’ | At4g32850 | TGTGCCACAGGATCTGGATATCTCCAGCTC | Genotyping Arabidopsis mutants |
| SALK_063790 3’ | At4g32850 | CCATACTATCTACTTCAAGAACTTGCTC | Genotyping Arabidopsis mutants |
| SALK_007979 5’ | At4g32850 | TTCATCATCTTGCATGATATATTGGCT | Genotyping Arabidopsis mutants |
| SALK_0079793’ | At4g32850 | ACGGTCCCAGACAGGAAATCCAAGCTCA | Genotyping Arabidopsis mutants |
| Lba1 | SALK T-DNA | ATGGTTCACGTAGTGGGCCATCG | Genotyping Arabidopsis mutants |
| P475 | WiscDsLox T-DNA | AACGTCCGCAATGTGTTATTAAGTTGTC | Genotyping Arabidopsis mutants |
| PI5’1SalI | At1g17980 | GTCGACGGGGTTTATTCAGATGCATTATTAG | Cloning promoters |
| PI3’1NcoI | At1g17980 | CCATGGCTATCCTACTTACTTTGC TCTTTTC | Cloning promoters |
| PII5’1SalI | At2g25850 | GTCGACTTCACGCGCATGAGCAGACCA | Cloning promoters |
| PII3’1NcoI | At2g25850 | CCATGGGACAAGACGCGTAAACACTAGAAAA | Cloning promoters |
| PIII5’1SalI | At3g06560 | GTCGACTTCCGATCAGAGAAGCAACGATCAA | Cloning promoters |
| PIII3’1NcoI | At3g05650 | CCATGGCAGCTACGAAATTTTAAAGAAAGT | Cloning promoters |
| PIV5’1SalI | At4g32850 | GTCGACTGGATTCTAAAATAATAAAGTTTCCTCTCC | Cloning promoters |
| PIV3’1Nco | At4g32850 | CCATGGCATAACACGATTGATAATCCTTAAG | Cloning promoters |
| 5’Sal I | At1g17980 | GTCGACATGGCTAGTGTCCAGCAAAATGGGCAACGG | PCR amplification for GFP fusions |
| 3’APA I | At1g17980 | GGGCCCTTACTTGCCATTGGTTTTGCCTAGAGACGT | PCR amplification for GFP fusions |
| 5’16 Bgl II | At2g25850 | AGATCTATGGTGAGTACTCAACAACGCACGGACG | PCR amplification for GFP fusions |
| 3’11 Bgl II | At2g25850 | AGATCTTCTGATTTGCATAAACCAATAATGGGTT | PCR amplification for GFP fusions |
| 5’7 Sal I | At3g06560 | GTCGACATGGCTAGTGTCCAGCAAAATGGGCAACGG | PCR amplification for GFP fusions |
| 3’6 Bam HI | At3g05650 | GGATTCACATTTAACCTCCATACCATCAGCTTCTCT | PCR amplification for GFP fusions |
|  |  |  |  |
| **plasmid** | **plasmid** | **source** | **reference** |
| pGDR-PAPS4 | At4g32850 | K. P. Forbes | {Forbes, 2004 #257} |
